# Metabolic reprogramming in saliva of mice treated with the environmental and tobacco carcinogen dibenzo[*def*,* p*]chrysene

**DOI:** 10.1038/s41598-024-80921-1

**Published:** 2024-11-27

**Authors:** Yuan-Wan Sun, Kun-Ming Chen, Cesar Aliaga, Karam El-Bayoumy

**Affiliations:** Department of Biochemistry and Molecular Biology, Pennsylvania State University College of Medicine, 500 University Drive, Hershey, PA 17033 USA

**Keywords:** Tobacco and Environmental Carcinogens, Oral squamous cell carcinoma, Mouse model, Saliva, Metabolic profiles, Oral cancer, Cancer, Biomarkers

## Abstract

The goal of this study is to develop a non-invasive approach for early detection of oral squamous cell carcinoma (OSCC) using our established mouse model that faithfully recapitulates the human disease. We present for the first time a comparative metabolomic profiling of saliva samples of the tobacco smoke constituent, dibenzo[*def*,* p*]pyrene, (DB[*a*,* l*]P) vs. DMSO (control)-treated mice using an established and highly sensitive LC-MS/MS approach. DB[*a*,* l*]P was administered by topical application into the mouse oral cavity (25 µmol, 3x week for 6 weeks) and saliva was collected 24 h after the last dose of carcinogen administration. Using an untargeted metabolomics approach (negative and positive modes), we found that DB[*a*,* l*]P differentially altered several metabolites known to be involved in the carcinogenesis process when compared to DMSO. Of particular significance, we found that DB[*a*,* l*]P significantly enriched the levels of phosphatidic acid, known to bind and activate mTORC which can enhance proliferation and promote carcinogenesis. Pathway enrichment analysis revealed that DB[*a*,* l*]P altered two major lipid metabolism pathways (phospholipid biosynthesis and glycerolipid metabolism). Collectively, our results using saliva as a safe and non-invasive approach, provide additional mechanistic insights on DB[*a*,* l*]P-induced OSCC and potential biomarkers for early detection and an opportunity for cancer interception *via* reprogramming lipid metabolism.

**Supplementary Information:**

The online version contains supplementary material available at 10.1038/s41598-024-80921-1.

## Introduction

Tobacco smoke is an established etiological factor for the development of oral squamous cell carcinoma (OSCC)^1^. Overall, the 5-year survival rate of OSCC remains ~ 60% and is improved if diagnosed at early stage^2^. The current gold standard for diagnosis of OSCC is a scalpel-obtained biopsy followed by histological interpretation; however, this approach does not capture the heterogeneous nature of this disease. Despite some advances in diagnostic tools, the percentage of OSCC diagnosed at early stages has not improved significantly since most of the patients are asymptomatic and mucosal changes may not be detected by visual inspection of the oral cavity^3^. Clearly there is an urgent need to develop a non-invasive approach for early detection of the disease. Previous studies aimed at identifying biomarkers for early detection have primarily focused on molecular targets that arise at end stages of disease (OSCC) even though these changes can be considered as a consequence of cancer development. Thus, our approach to develop biomarkers for early detection and effective strategies for cancer interception/prevention is to utilize a mouse model of oral cancer induced by dibenzo[*def*,* p*]chrysene (also known as dibenzo[*a*,* l*]pyrene, DB[*a*,* l*]P), an environmental pollutant and tobacco smoke constituent. We have shown that the oral cancer induced by DB[*a, l]*P progresses from hyperplasia, through dysplasia and carcinoma *in situ* to OSCC, exhibit heterogeneity and reflect the cellular and molecular targets (e.g. COX-2) observed in humans^1,4–6^.

We recently reported the detection of DNA adducts derived from DB[*a*,* l*]P in buccal cells of smokers by isotope dilution LC-MS/MS, suggesting the potential contribution of this carcinogen in the development of OSCC^7^. Moreover, we found that, prior to the detection of any histological abnormality in the mouse oral cavity, DB[*a*,* l*]P-induced epigenetic changes such as hypomethylation of *Fgf3*, which is known to be involved in epithelial-mesenchymal transition pathway; in fact our results revealed that hypomethylation of *Fgf3* is a plausible biomarker for early detection of OSCC^8^. Since the histological and molecular changes identified in the mouse oral cavity following DB[*a*,* l*]P treatment mimic those of human OSCC, our animal model is utilized in the present study as a realistic platform to further examine the metabolic changes in the early stage of oral carcinogenesis.

Metabolomic studies can assist in identifying clinically relevant biomarkers for early detection, improve diagnosis and prognosis and in the development of preventative and treatment strategies^9^. Different types of biological samples from animals and humans, including saliva, serum, blood, urine and tissues have been used in metabolomic profiling and the advantages and disadvantages have been discussed^10^. Saliva is readily available and the collection process is simple, inexpensive and non-invasive; it is an important biological fluid required for multiple basic functions (speech, taste, digestion of foods, antiviral and antibacterial protection) to maintain adequate oral health. Saliva contains a variety of metabolites, some of which might be associated with oral or systemic diseases and thus metabolomic analysis of saliva could be useful for identifying disease-specific biomarkers. The metabolites are the final products of cellular biochemical processes, including gene transcription, mRNA translation, protein synthesis, and metabolic enzymatic reactions^11^. The metabolome is a complex mixture of lipids, steroids, peptides, oligonucleotides, sugars, aldehydes, ketones, amino acids, alkaloids, and metabolomic profiles represent phenotypic responses for endogenous processes and exogenous exposure. However, at present a common metabolic signature has yet to be identified to be implemented in the clinic. Therefore, as an initial investigation to provide insights in the metabolic changes using our well-established oral cancer animal model, we examined for the first time, the effect of the oral carcinogen, DB[*a*,* l*]P, on saliva metabolic profiles using untargeted metabolomics in the negative and positive modes at an early time point prior to the detection of any morphological changes, but under the experimental conditions, established in our laboratory, known to induce maximum DNA damage which is essential for the development of OSCC in this animal model^5,12^.

## Materials and methods

### Animal treatment, saliva sample collection and ethical principles

Our animal studies were conducted according to the ARRIVE guidelines 2.0 and the NIH, USA and AAALAC International Regulations. The Institutional Animal Care and Use Committee (IACUC) of Penn State College of Medicine reviewed and approved all experiments prior to their initiation (IACUC Protocol No. PROTO201900755; Approval Date (7/25/23); Renewed Date: 7/25/24). In this study we used our established murine model utilizing a relevant tobacco smoke constituent DB[*a*,* l*]P to induce OSCC^5^. Briefly, five week old B6C3F1 female mice were divided into two groups (*n* = 3/group) and treated with DMSO (vehicle) or DB[*a*,* l*]P (24 nmol dissolved in DMSO). The sample size (*n* = 3 per group) was employed based on extensive previous studies^8,13^ performed in our laboratory demonstrating that *n* = 3 was sufficient for DB[*a*,* l*]P to observe significant DNA damage; a prerequisite step in the multi-step carcinogenesis process, as well as epigenetic alterations in the mouse oral cavity^8,12,14,15^. The treatments were applied 3 times per week *via* topical application into the oral cavity for a period of 6 weeks as described previously^5^. Body weight were recorded weekly; examinations of oral morphological abnormalities including the mouth and nose area were also performed weekly. Twenty-four hrs. after the last dose of carcinogen treatment, mice were anesthetized using ketamine/xylazine followed by intraperitoneal injection of carbachol (0.1 mg/kg) in normal saline, an established method to induce saliva secretion which were collected using a capillary tube attached to a sterile microcentrifuge vial; approximately 100 µL/mouse of saliva were collected^16^. After saliva collection, mice were euthanized by CO_2_ followed by cervical dislocation. Samples were stored at -80 °C until processing.

### Sample preparation for metabolome analysis

After thawing saliva samples on ice, 200 µL of 80% methanol was added to each sample followed by vortexing for 30 s. and sonication for 30 min at 4 °C. The samples were then kept at -20 °C for 1 h, then vortexed for 30 s. and kept at 4 °C for 30 min before centrifugation at 12,000 rpm at 4 °C for 15 min. The supernatant was transferred to a new tube and incubated at -20 °C for 1 h before centrifugation at 12,000 rpm and 4 °C for 15 min. The supernatant ( 200 µL) was added to DL-o-Chlorophenylalanine (0.14 mg/mL) and transferred to a vial for LC-MS analysis. The same amount of supernatant was obtained from each sample and mixed as quality control (QC) samples which were used to evaluate the methodology described in this study.

## UPLC-ESI-MS untargeted metabolomic profiling

**Fig. 1:**
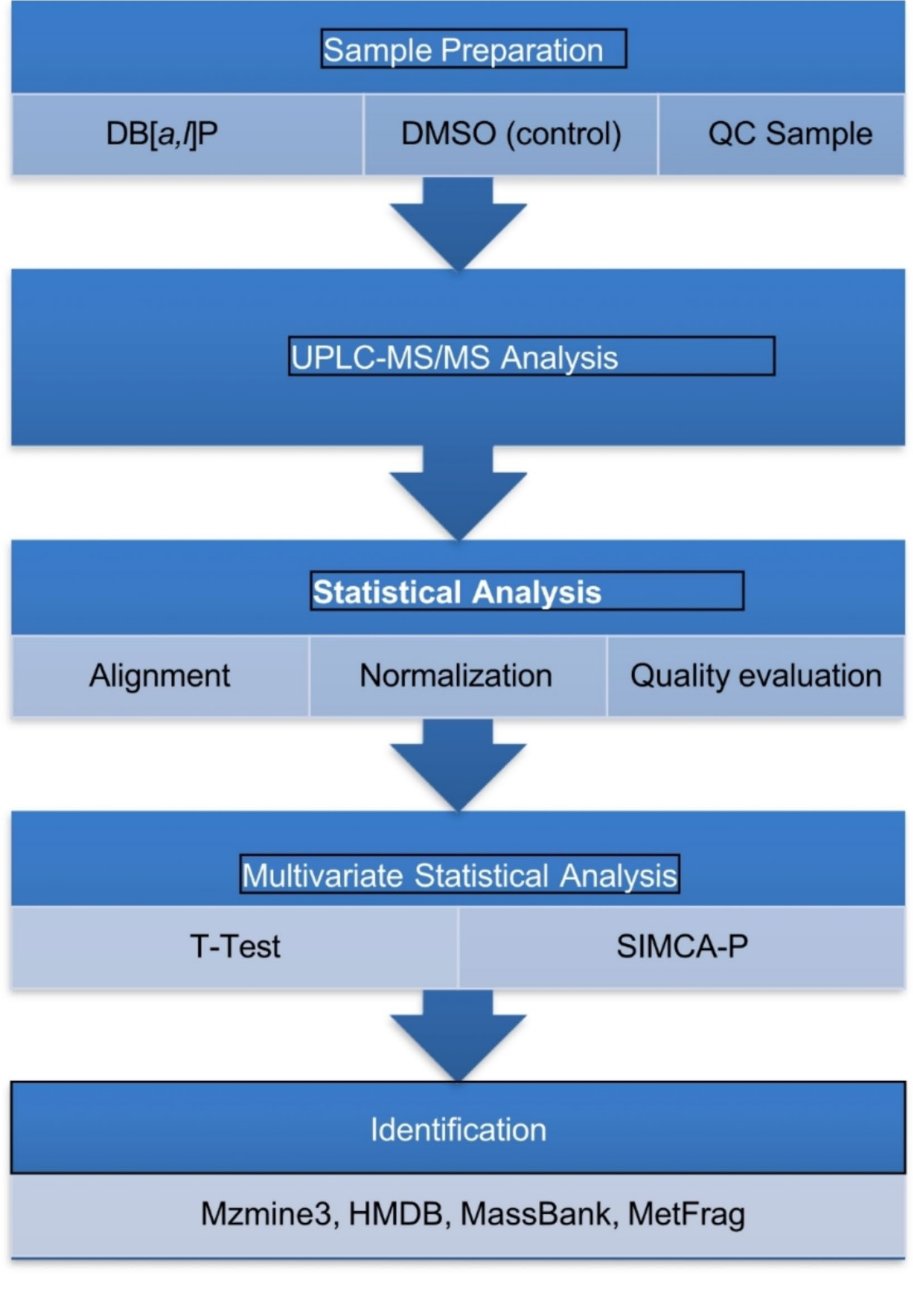
Workflow of untargeted metabolomics.

Metabolite separation was performed by Vanquish Flex UPLC equipped with Q Exactive plus (Thermo). The LC system contains a waters T3 column (100 × 2.1 mm×1.8 μm) with the mobile phase composed of solvent A (0.05% formic acid water) and solvent B (acetonitrile) using a gradient elution (0–1 min, 5% B; 1–12 min, 5-95% B; 12–13.5 min, 95% B; 13.5–13.6 min, 95%-5% B; 13.6–16 min, 5% B) at a flow rate of 0.3 mL/min. The column temperature and sample manager temperature were maintained at 40 °C and 4 °C, respectively. Both positive and negative ESI-MS modes were used to screen the metabolites. Mass scan mode: Full Scan (m/z 70 ~ 1050, Resolution: 70,000) and dd-MS2 (TopN = 10, Resolution: 17,500); Collision mode: HCD; mass spectrometry parameters are listed as follows: ESI positive: Heater Temp 300 °C; Sheath Gas Flow rate, 45 arb; Aux Gas Flow Rate, 15 arb; Sweep Gas Flow Rate, 1 arb; spray voltage, 3.0 kV; Capillary Temp, 350 °C; S-Lens RF Level, 30%. ESI negative: Heater Temp 300 °C, Sheath Gas Flow rate, 45 arb; Aux Gas Flow Rate, 15arb; Sweep Gas Flow Rate, 1 arb; spray voltage, 3.2 kV; Capillary Temp, 350 °C; S-Lens RF Level, 60%. Workflow of untargeted metabolomics is depicted in Fig. 1.

## Metabolite data processing and analysis

The raw data are acquired and aligned using the Compound Discoverer (3.0, Thermo) based on the m/z value and the retention time of the ion signals. Ions from both ESI negative or ESI positive are merged and imported into the SIMCA-P program (version 14.1) for multivariate analysis. A Principal Components Analysis (PCA) is first used as an unsupervised method for data visualization and outlier identification. Supervised regression modeling is then performed on the data set by use of Partial Least Squares Discriminant Analysis (PLS-DA) or Orthogonal Partial Least Squares Discriminant Analysis (OPLS-DA) to identify the potential biomarkers. The biomarkers are filtered and confirmed by combining the results of the Variable Importance in Projection (VIP) values (VIP > 1.5) and t-test (*p* < 0.05). The quality of the fitting model can be explained by R^2^ and Q^2^ values; R^2^ displays the variance explained in the model and indicates the quality of the fit and Q^2^ displays the variance in the data, indicating the model’s predictability. All significant metabolites are imported to obtain the categorical annotations, including pathways, enzyme interactions and other biological annotations. Pathway enrichment analysis was performed using MetaboAnalyst under the condition of *p* < 0.05.

## Results

### Analysis of saliva metabolite profile change induced by DB[*a*,* l*]P

**Fig. 2:**
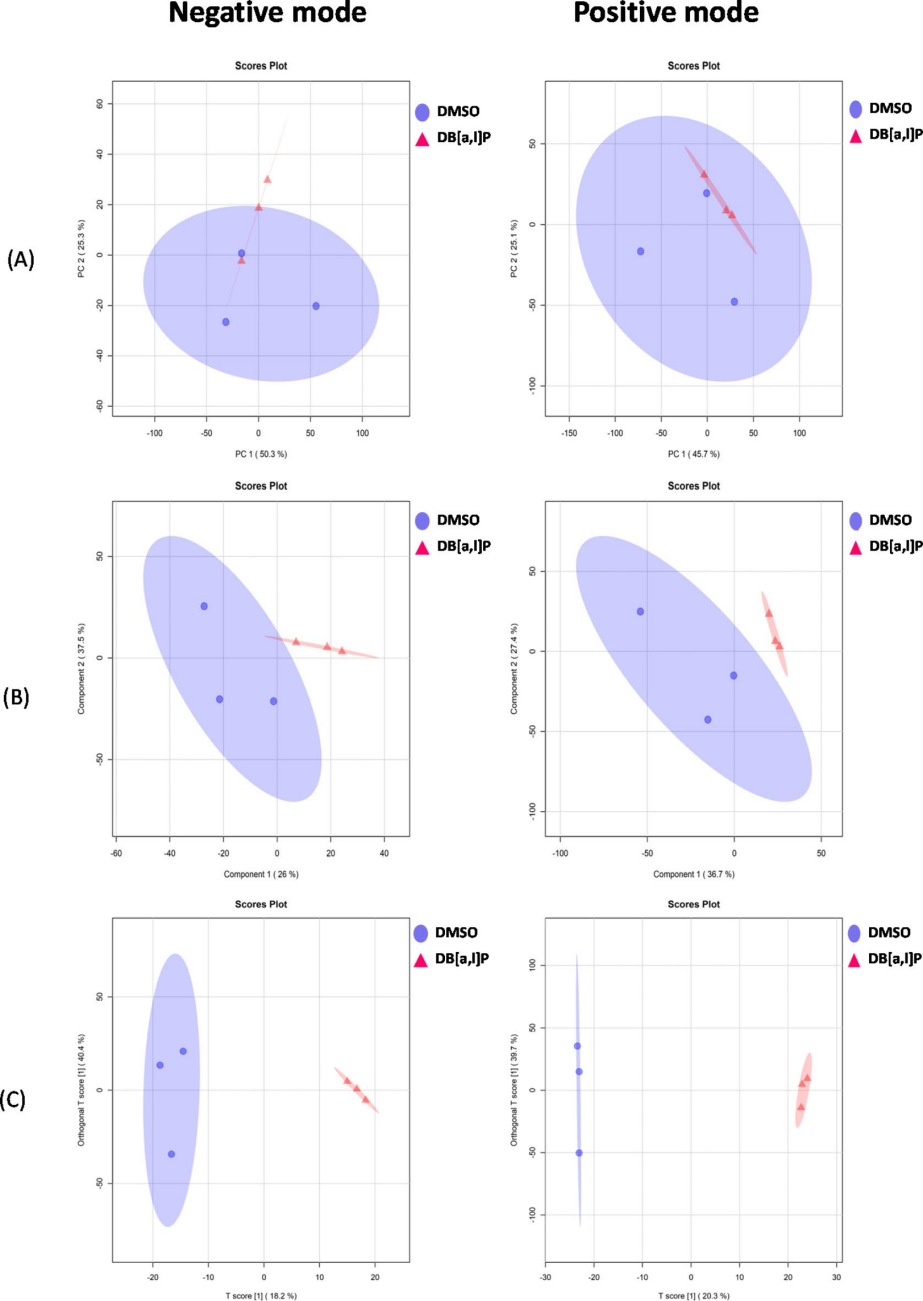
The score scatters plots of (**A**) PCA (**B**) PLS-DA and (**C**) OPLSD-DA models for both negative and positive es.

**Fig. 3:**
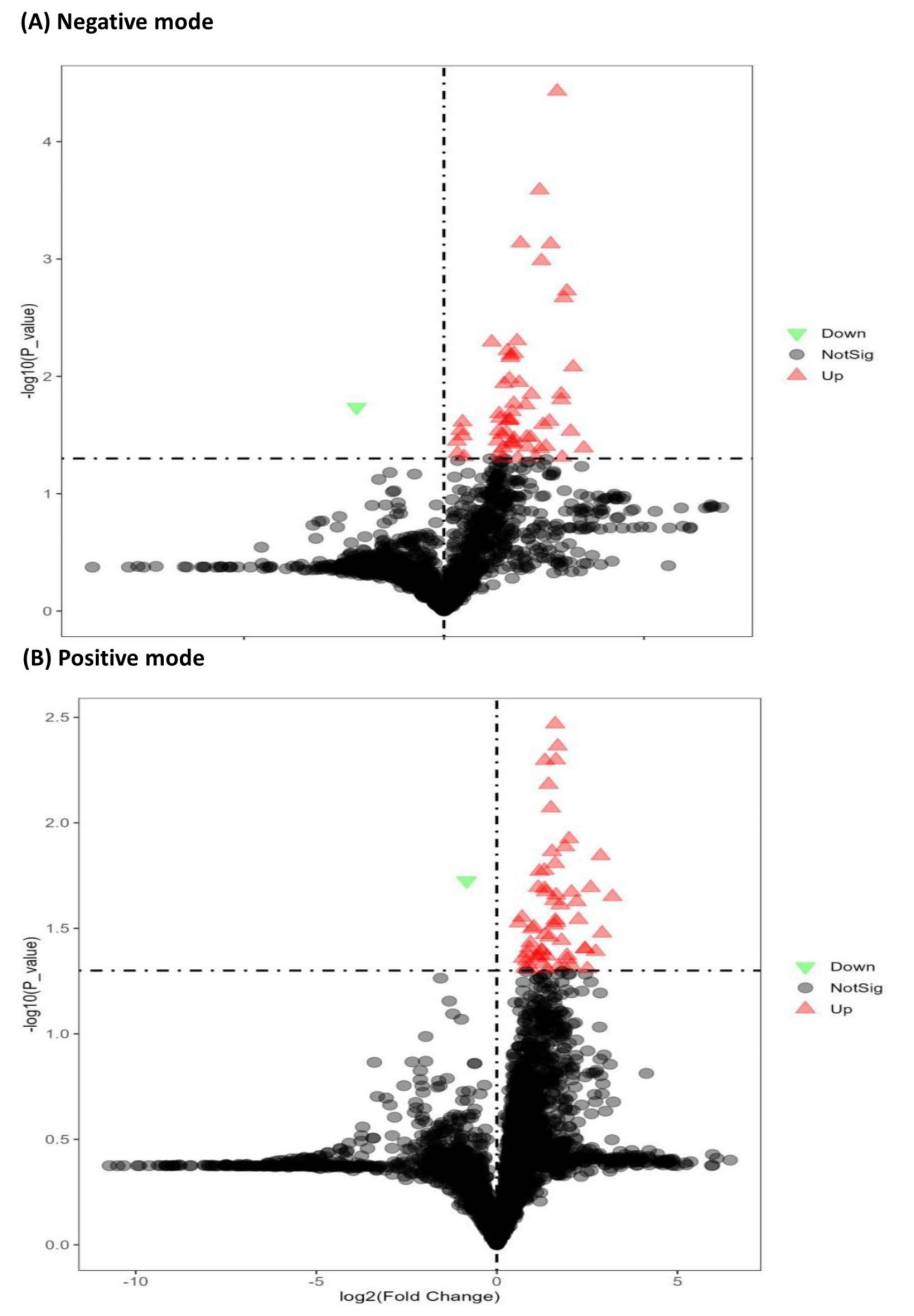
Volcano plot for saliva metabolome changes. Based on the fold change (X-axis) and P-value (Y-axis). The range of Y > 1.30 and X > 0 were significant increase (red); The range of Y > 1.30 and X < 0 were significant decrease (green).

Representative total ions chromatographs (TIC) of the sample are shown in Supplementary Figures S1A (ESI negative mode) and S1B (ESI positive mode). QC samples are used to demonstrate the stability of the LC-MS system. The QC samples run in positive and negative mode at regular intervals throughout the entire sequence. The ion features of the QC samples were used to calculate the relative standard deviation (RSD). The percent RSD distribution showed an overwhelming majority of the RSD was less than 30% (data not shown) which indicated the analysis procedure was robust and thus used for subsequent sample analysis. **I**ons from both ESI negative and ESI positive modes were merged and imported into the SIMCA-P program (Version 14.1) for multivariate analysis. To investigate the global metabolism variations, a PCA was first used as an unsupervised method for data visualization and outlier identification in both ion modes (ESI negative and ESI positive). Figure 2 A shows the PCA plot which exhibits an unclear grouping trend between control and DB[*a*,* l*]P treated groups. Supervised regression modeling was then conducted on the dataset using PLS-DA and OPLS-DA to identify the metabolites with potential significant differences. The score plots for PLS-DA and OPLS-DA are shown in Fig. 2B and C, respectively; a clear separation between groups were observed in both plots. Significantly changed metabolites were then filtered out based on VIP values (VIP > 1.5) and *P* < 0.05. The distribution of VIP values of all the metabolites are shown in Supplementary Figure S2A and S2B. Univariate analysis including fold change (FC) analysis and t-test were performed on volcano plot as shown in Fig. 3A (negative mode) and Fig. 3B (positive mode). The range of Y > 1.30 and X > 0 showed significant increase; the range of Y > 1.3 and X < 0 showed significant decrease.

### Identification of potential biomarkers

The chemical structures of metabolites were identified according to online databases such as the Human Metabolome Database (www.hmdb.ca) using the data of accurate masses and MS/MS fragments. Further confirmation was acquired, if necessary, through comparisons with authentic standards using both retention times and MS/MS fragmentation patterns. Table 1 shows the list of metabolites with significant changes. All the metabolites identified under ESI negative and ESI positive scans are provided in Supplementary Table 1.

**Table 1:** Metabolites that showed significant changes in the saliva of mice treated with DB[a, l]P.

Metabolite name	Molecular mass	Mode	Fold change	*p*-value
PA(15:0/LTE4)	817.4563703	−	8.412731001	0.001878945
PS(20:5-3OH(5,6,15)/18:4)	849.4428283	−	9.356787294	0.008332525
Tyr-Gly-Gly-Trp-Leu	594.2801976	−	4.543251788	0.014238094
Leucylleucine	244.1786926	−	0.220194702	0.018320118
Menatetrenone	444.3028305	−	6.222874447	0.024158261
8-Amino-7-oxononanoic acid	187.1208434	+	3.113703995	0.005037935
LysoPA(20:3/0:0)	460.2589907	+	0.560439281	0.01880815
Icosa-5,8,11,14-tetraene	274.2661	+	6.050328801	0.020286987
bemegride	155.0946287	+	2.498457784	0.020505175
Propylthiouracil	170.0513836	+	2.56445509	0.021224649
Cannabinol	310.1932801	+	3.123272011	0.022001077
Phenylalanyllysine	293.1739416	+	3.003967599	0.023421789
Gluten exorphin A5	599.2591278	+	4.632279463	0.023686368
5-Phenyl-1,3-pentadiyne	140.0626003	+	2.032096372	0.031203546
Dehydronorketamine	221.0607417	+	2.386247402	0.040503006
Butyrylcarnitine	231.1470582	+	1.803483307	0.04148744
Thioridazine	370.1537402	+	3.254745238	0.048111134
Tert-Butylbicyclophosphorothionate	222.0479525	+	1.94797632	0.049545995
Lysylglycine	203.1269914	+	2.645247003	0.049673499

**Fig. 4:**
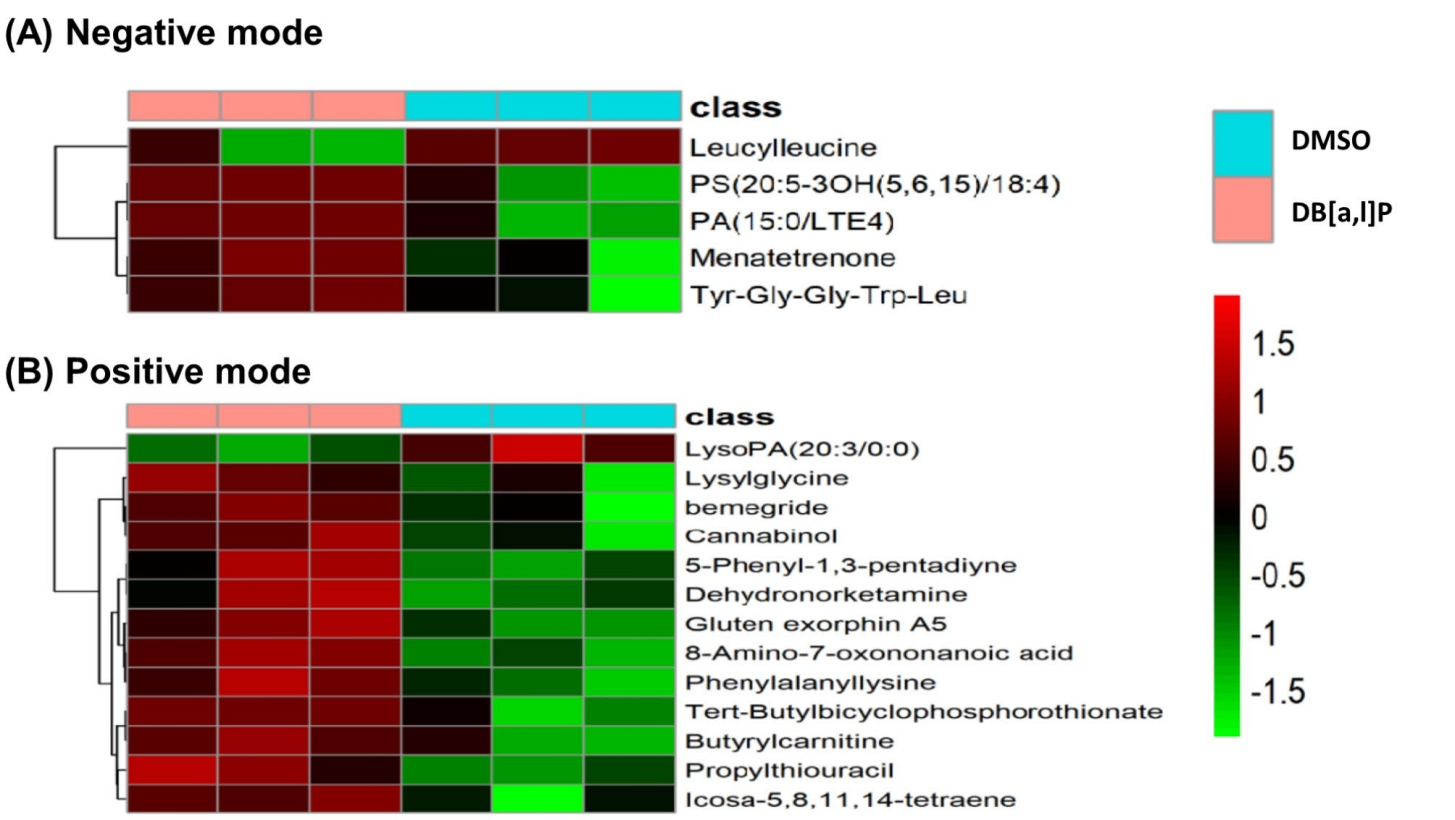
Hierarchical cluster analysis of metabolome data from significant metabolites.

Hierarchical Cluster Analysis (HCA) of metabolome data was also performed. Mean values of metabolite contents from biological replicates are used to calculate the metabolite ratio. After log transformation of the data, median centered ratios are normalized. HCA is performed using the complete linkage algorithm of the program Cluster 3.0 (Stanford University) and the results are visualized using heatmap 1.0.12 (Raivo Kolde). Metabolite ratios from two independent experiments of significant metabolites are used for HCA. Color intensity correlates with degree of increase (red) and decrease (green) relative to the mean metabolite ratio. We found DB[*a*,* l*]P as compared to DMSO significantly (VIP > 1.5, *p* < 0.05) decreased the levels of Leucylleucine but increased the levels of phosphatidylserine (PS) (20:5–3OH (5, 6,15)/18:4), phosphatidic acid (PA) (15:0/LTE4), Menatetrenone (isoform of Vitamin K2), and Tyr-Gly-Gly-Trp-Leu in ESI negative mode **(**Fig. 4A**)**. In the ESI positive mode, HCA of metabolome data analysis (Fig. 4B), DB[*a*,* l*]P altered several metabolites associated with lipid metabolism (e.g. LysoPA 20:3/0:0), amino acid derivatives and purine metabolism.

### Pathway enrichment analysis

For pathway enrichment analysis, only well-annotated HMDB compounds were applied to MetaboAnalyst. Under the condition of *p* < 0.05, two pathways namely glycerolipid metabolism and phospholipid biosynthesis were identified using data set in ESI negative mode; however, in ESI positive mode, no pathways were identified.

## Discussion

In the current study, we present for the first time a comparative metabolomic profiling of saliva samples of DB[*a*,* l*]P vs. DMSO-treated mice using an established and highly sensitive LC-MS based approach^17,18^. Clearly DB[*a*,* l*]P significantly impacted lipid metabolism (glycerolipid metabolism, phospholipid biosynthesis) as the primary metabolic pathways. Fatty acids are the main building blocks of several lipid species including phospholipids, sphingolipids and triglycerides and can be funneled into various metabolic pathways to synthesize more complex lipid species including diacylglycerides and triacylglycerides. Fatty acids can also be converted into phosphoglycerides such as PA, PS and phosphatidylethanolamine (PE)^19^. Aside from their principal role as structural components of the membrane matrix, fatty acids are important secondary messengers and can serve as fuel source for energy production.

Since Otto Warburg formulated the original hypothesis that the metabolism differs between normal and cancer cells, the field of metabolomics has rapidly increased^9,20^. There is an increased reliance of cancer cells on *de novo* biosynthesis and exogenous fatty acids uptake to sustain their proliferative rate. In the present study, we used LC-MS/MS based untargeted metabolomics approach to better understand the complex metabolic changes and their association with pathways relevant to carcinogenesis at early time point in the development of OSCC-induced by DB[*a*,* l*]P, an environmental pollutant and a tobacco smoke constituent. Metabolites are the final embodiment of life activities, and the small changes in phenotypes will be amplified exponentially at the metabolic level. Previous studies have been heavily focused on identifying tumor-specific biomarkers at end stage of disease (OSCC) using multiple omics technology including metabolomics;^11^ however, these biomarkers could be considered a consequence of cancer development and cannot be utilized as early biomarkers for disease development. Therefore, our established preclinical oral cancer mouse model was utilized as a reliable platform to collect saliva samples prior to the detection of any morphological changes in the oral cavity with the ultimate goal of discovering biomarkers for early detection of this disease. This is the first study reporting on the effects of DB[*a*,* l*]P on mouse saliva metabolomics in an established mouse model of OSCC.

Both stimulated and unstimulated saliva have been used for metabolomic studies. In order to collect an ample amount of mouse saliva, in this study the muscarinic agonist carbachol was used to stimulate secretion of saliva^16^. Using untargeted metabolomics approach, we found that DB[*a*,* l*]P, significantly enriched several metabolites (Table 1) when compared to DMSO treated mice including several PA and PS which have been reported to be associated with glycerophospholipid metabolism^18^. The lipid metabolism pathway is linked to several health disorders including cancer development^21–23^. PA is known to bind and activate the mTORC^19^, which can enhance proliferation and promote the carcinogenesis. PS has been reported to possess beneficial effects in humans (e.g. improve memory, learning mood, stress management)^24^. However, oxidized phosphatidylserine (OXO-PS) in which a phosphorylserine moiety occupies a glycerol substitution site with at least one of the fatty acyl chains being oxidized. Although OXO-PS has been shown to possess immunosuppressive effects, future studies are required to fully understand its biological activities^25^. Glycerophospholipids are important cell membrane constituents that maintain structure integrity and play an important role as signaling molecules^26^. Although lipids can be oxidized by free radicals, several enzymes including COX, LOX, PGE2 and CYP450;^27^ are known to catalyze the biosynthesis of oxidized lipids. PGE2 is known to activate downstream signals (PI3K/AKT/mTORC) and can act as a tumor promotor^28^. Of particular relevance, we previously showed that DB[*a*,* l*]P-enhanced COX-2 protein expression in the mouse oral cavity^5^. It is likely that elevated expression of COX-2 can enhance the lipid oxidation during the development of OSCC induced by DB[a, l]P. On the other hand, we found the level of lysophosphatidic acid (LysoPA, 20:3 [8Z,11Z, 14Z]/0:0) was decreased by DB[*a*,* l*]P treatment. Although present at very low levels in animal tissues, LysoPA is important biologically in influencing many biochemical processes; certain LysoPA analogs have been reported to act as endogenous PPAR_γ_ ligands^29^. Activation of PPAR_γ_ can block NF-κB translocation to the nucleus and exert anti-inflammatory signaling and in fact, literature data supports that PPAR_γ_ and NF-κB are interdependent^30–32^.

At present, literature data remain to lack coherency regarding the nature of specific lipids which could be due to the variations in populations (smokers vs. nonsmokers, OSCC patient’s vs. healthy subjects), analytical methods, and/or biological fluids (saliva, serum, tissues). Previous studies found that metabolomic profiles are different between serum and tissues in HNSCC patients and lipids were significantly overexpressed in tumor tissues from the HNSCC tissue samples as compared to serum^33,34^. However, the metabolomic profiles of saliva vs. oral tissues in HNSCC patients remain undefined. In healthy subjects, Hsu et al. showed that serum metabolites were affected by acute cigarette smoking^35^, and reported the reduction of 13 glycerophospholipids including 11 LysoPA; however, these results were inconsistent with those reported that showed changes in certain members of phospholipids were either higher or lower in smokers than nonsmokers^36–38^. Clearly a common metabolic signature remains largely undefined and thus studies using animal models that mimic human disease as employed in the present study can avoid the possible variations observed across various human studies. Using ^1^HNMR, Kong et al. reported the changes of serum metabolic profiles during different stages of oral carcinogenesis (oral leukoplakia and OSCC) induced by synthetic carcinogen (4-nitroquinoline-N-oxide) to induce OSCC in the rat^39^. However, to mimic human exposure, animal models, that employ tobacco/environmental agents rather than synthetic agents capable of inducing OSCC and saliva, a relevant biological fluid to the disease for routine examination could provide a more realistic platforms to better understand the mechanisms of carcinogenesis and discover biomarkers for early detection. Nevertheless, whether mice exposed to whole tobacco smoke will lead to the same changes of lipid metabolites identified in this study remains to be determined.

**Fig. 5:**
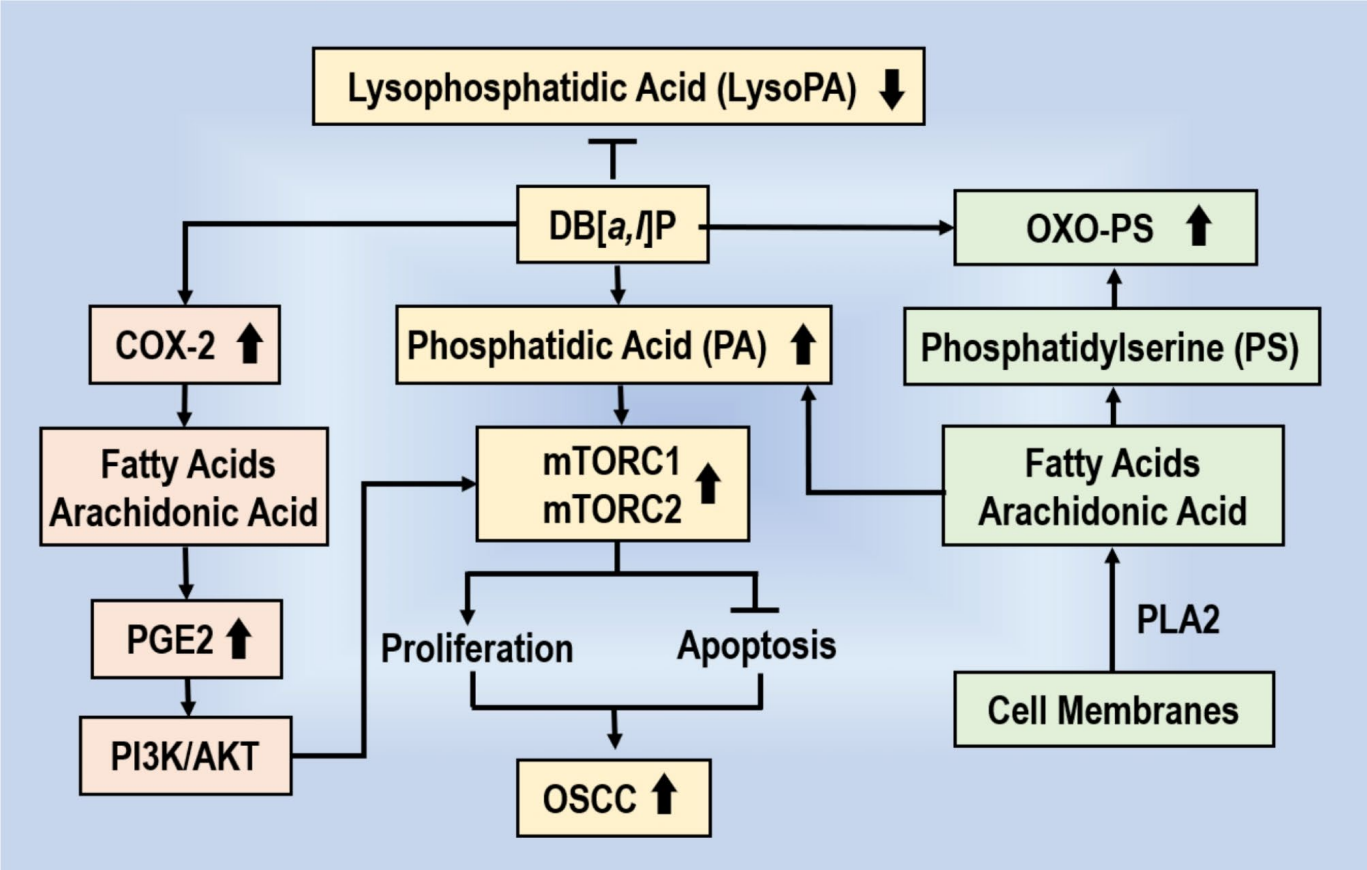
Proposed Mechanism on the Role of DB[*a*,* l*]P on Lipid Metabolism Leading to the Induction of OSCC.

In summary, our untargeted metabolomic study explored for the first time, the differences in saliva metabolomic profiles at early stage prior to the development of OSCC-induced by the tobacco carcinogen DB[*a*,* l*]P in an established mouse model of OSCC. This experimental approach resulted in the identification of several saliva lipid metabolites that can potentially be used as early biomarkers of OSCC; some of the identified metabolites can be highly predictive of carcinogenesis-induced by DB[*a*,* l*]P. Collectively, these identified metabolites and associated metabolic pathways provide strong foundation in the design of future interception/prevention of OSCC. On the basis of our metabolomics data, and those reported previously by us and others, we propose a mechanism (Fig. 5) that can contribute to the development of OSCC-induced by DB[*a*,* l*]P in mice.

However, there are several limitations in the present study including the lack of saliva metabolic profiles at different stages of disease progression as well as the comparative metabolic profiles between saliva and oral tissues. Furthermore, in our animal model, we examined the effect of a single carcinogen and not tobacco smoke as a whole on saliva metabolome. The effect of other etiological agents (betel quid chewing, alcohol consumption) on saliva metabolome remains undefined which constitutes another limitation of this study. In addition, our animal study cannot provide information on racial differences with regard to lipid metabolism as shown previously following acute cigarette smoking^35^. Future studies aimed at investigating the functional roles of other metabolites identified in saliva and the impact of microbiome on metabolomic profiles are needed.

## Electronic supplementary material

Below is the link to the electronic supplementary material.

Supplementary Material 1

## Data Availability

The datasets generated and/or analyzed during the current study are available from the corresponding author on reasonable request.
