# Supplementary material for: Metabolic reprogramming in saliva of mice treated with the environmental and tobacco carcinogen dibenzo[def, p]chrysene
Source: Sci Rep. 2024 Nov 27;14:29517. doi: 10.1038/s41598-024-80921-1 (PMC11603290; doi:10.1038/s41598-024-80921-1)
Supplement: Supplementary file 1 — Supplementary Material 1 [file 41598_2024_80921_MOESM1_ESM.pdf]

# Supplementary information

**Metabolic Reprogramming in Saliva of Mice Treated with the Environmental and Tobacco Carcinogen Dibenzo[*def,p*]chrysene**

Yuan-Wan Sun[1,] Kun-Ming Chen[1], Cesar Aliaga[1] Karam El-Bayoumy[1,*]

[1]Department of Biochemistry and Molecular Biology, Pennsylvania State University College of Medicine Hershey, PA 17033, US

*To whom correspondence should be addressed: Karam El-Bayoumy kee2@psu.edu; 500 University Drive, Hershey, PA 17033, US; Tel: 717-531-1079, Fax: 717-531-0002

**Supplementary Figure S1.** Metabolic profiling shows the total ions chromatograph (TIC) chromatograms of the sample in (A) ESI negative and (B) ESI positive modes.

**Figure S1A. TIC obtained from sample in ESI negative**

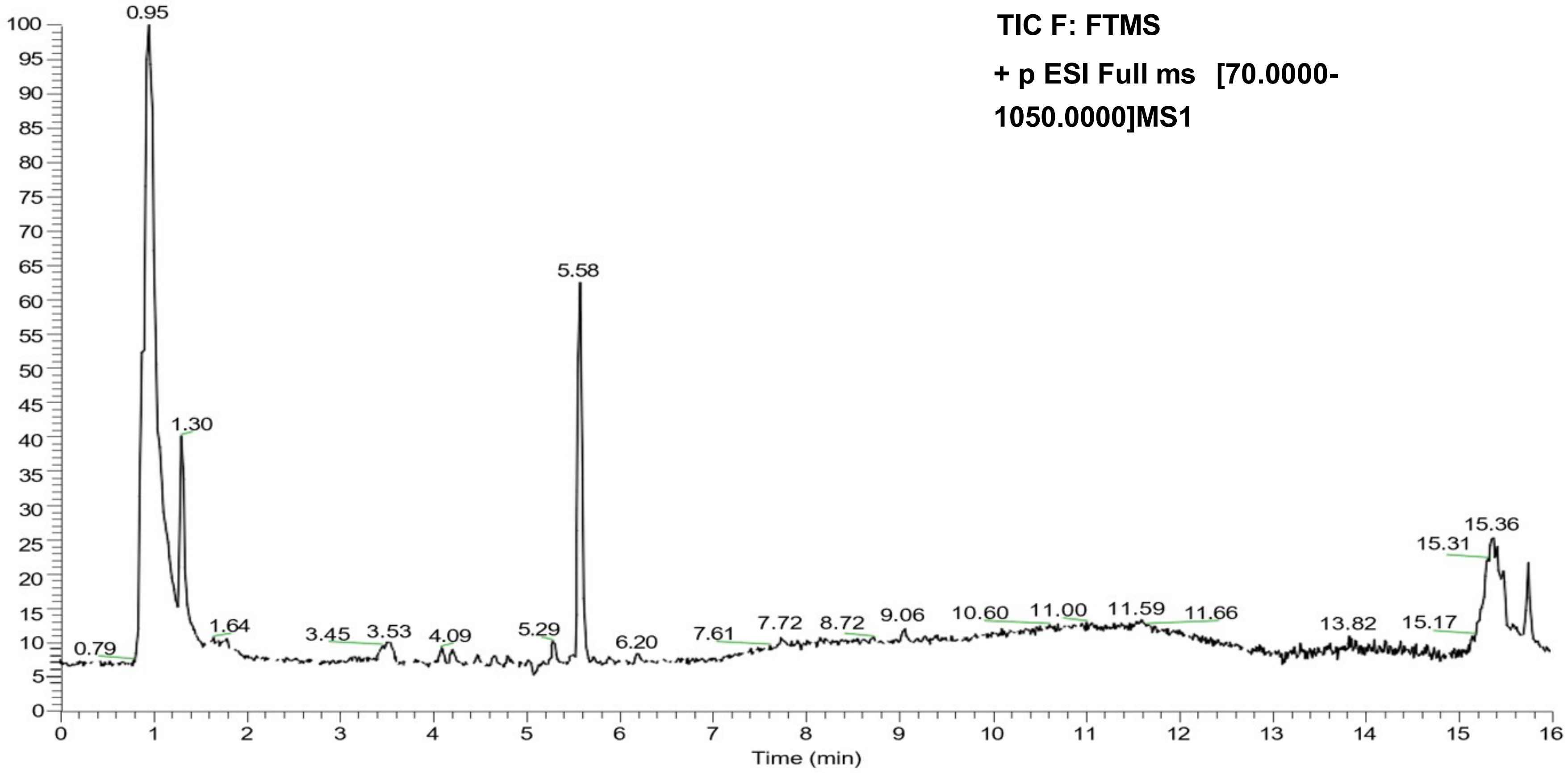

**Figure S1B. TIC obtained from sample in ESI positive mode.**

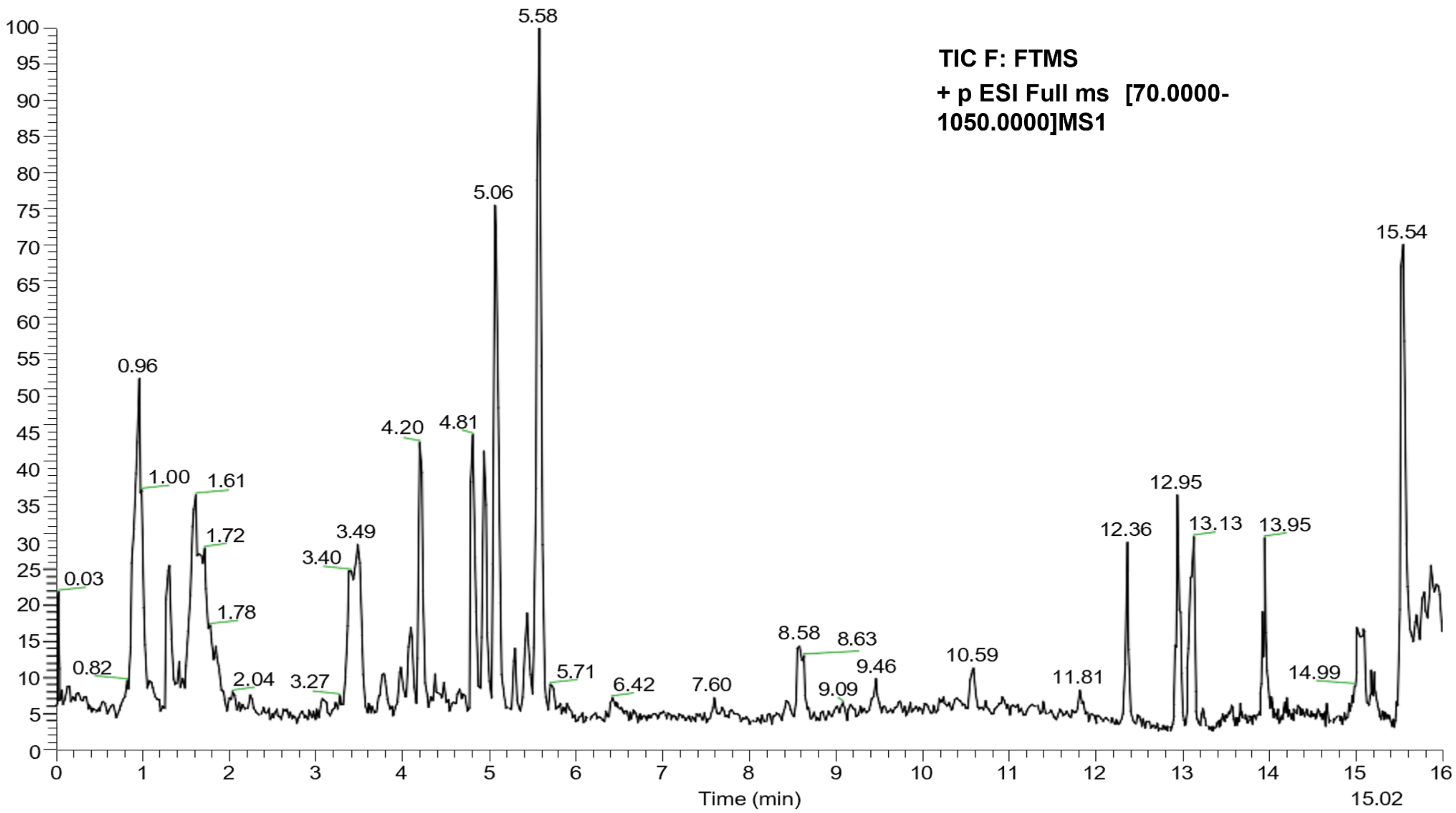

**Supplementary Figure S2.** (A) The distribution of numerical VIP values (B) The loading plot of PLS-DA model. The metabolites with red box were labeled as significant compounds (VIP > 1.5).

**(A)**

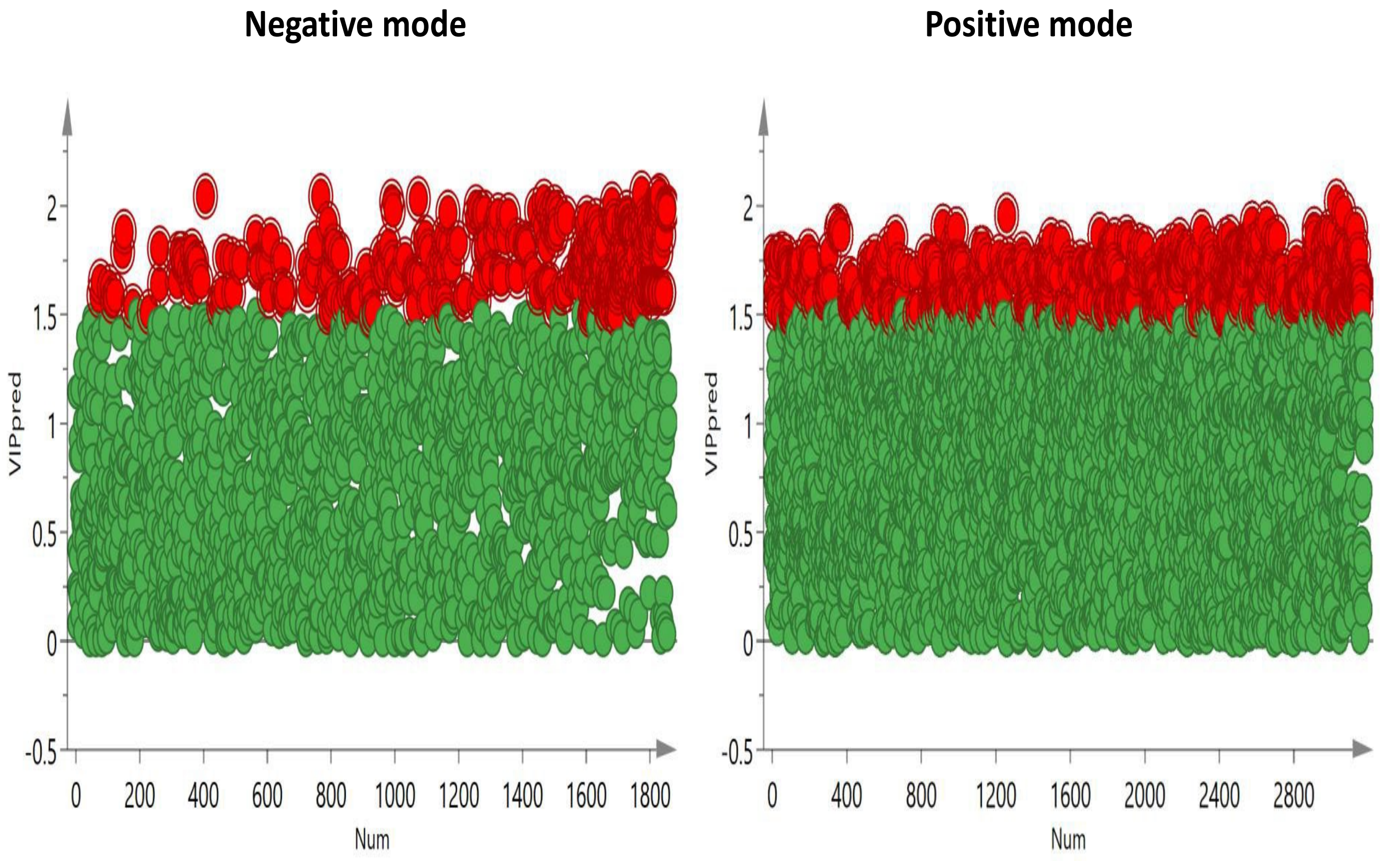

(B)

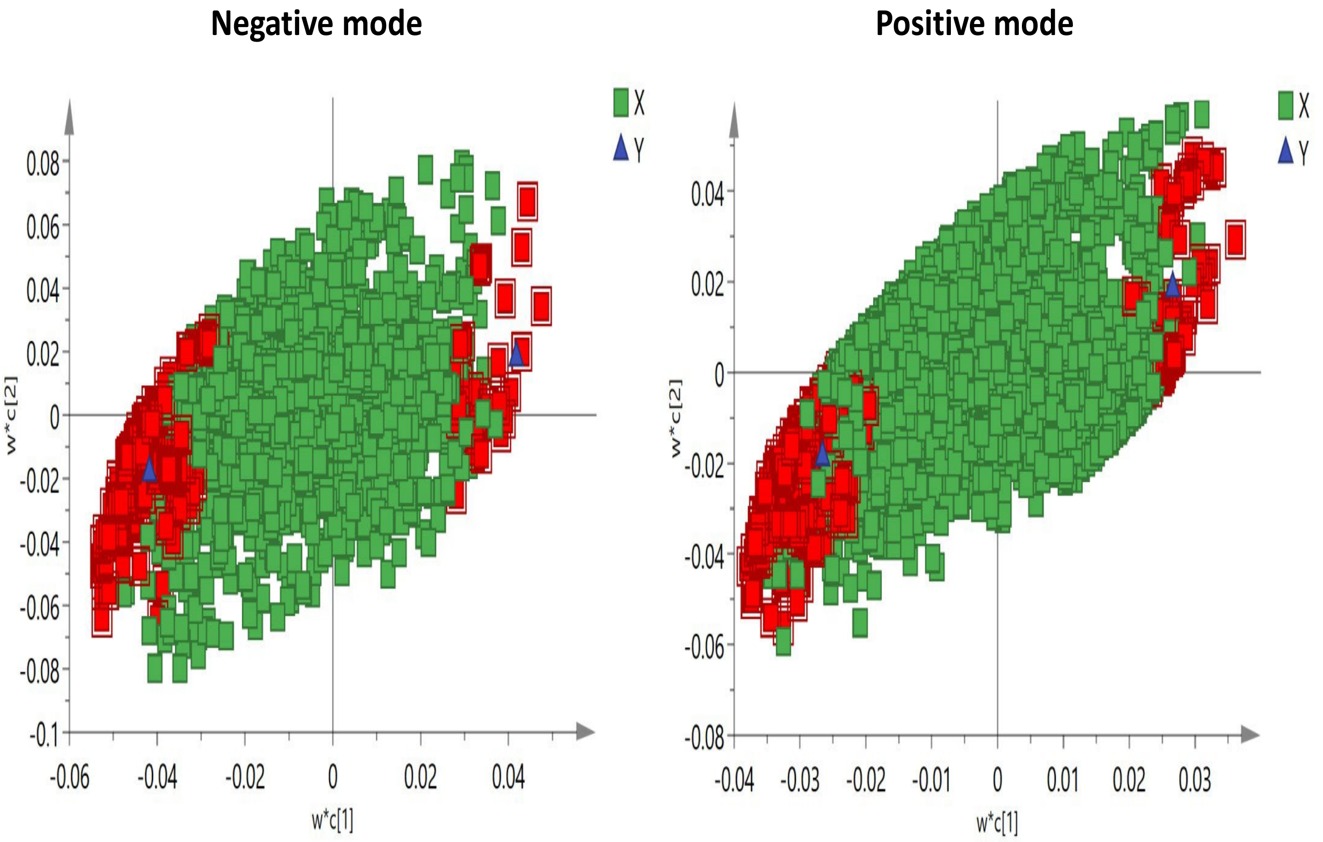
Negative mode
Positive mode
X
Y
w*c[2]
w*c[1]
0.08
0.06
0.04
0.02
0
-0.02
-0.04
-0.06
-0.08
-0.1
-0.06
-0.04
-0.02
0
0.02
0.04
-0.03
-0.01
0.01
0.03

## *Supplementary Table 1*
## Negative Mode

| HMDB_ID | Compound_name | DB]a,l]P-#1 | DB]a,l]P-#2 | DB]a,l]P-#3 | DMSO-#1 | DMSO-#2 | DMSO-#3 | AVE (DB[a,l]P) | AVE (DMSO) | FC (DB[a,l]P /DMSO) | Log2(FC) | T-Test | Log10 (P_value) | VIP |
|---|---|---|---|---|---|---|---|---|---|---|---|---|---|---|
| HMDB0263196 | PA(15:0/LTE4) | 67.91405957 | 80.68642102 | 83.49185928 | 25.24118075 | 0.933573192 | 1.413473932 | 77.36411329 | 9.196075956 | 8.412731001 | 3.072574215 | 0.001878945 | 2.726086033 | 2.036643695 |
| HMDB0282012 | PS(20:5-3OH(5,6,15)/18:4) | 205.7664443 | 218.1718914 | 224.0300343 | 65.06311702 | 2.798720282 | 1.389322277 | 215.9894567 | 23.08371986 | 9.356787294 | 3.226013257 | 0.008332525 | 2.079223386 | 2.012454056 |
| HMDB0259342 | Tyr-Gly-Gly-Trp-Leu | 53.49192355 | 87.79954294 | 92.17995501 | 26.89886271 | 23.23042377 | 1.259329507 | 77.82380717 | 17.12953866 | 4.543251788 | 2.183725262 | 0.014238094 | 1.846548148 | 1.675637933 |
| HMDB0028933 | Leucylleucine | 17.88271353 | 1.214894654 | 1.008554439 | 26.19512847 | 29.94559121 | 35.17011758 | 6.702054206 | 30.43694575 | 0.220194702 | -2.183148336 | 0.018320118 | 1.737071736 | 1.771359982 |
| HMDB0030017 | Menatetrenone | 60.36352448 | 138.0039438 | 120.9798348 | 16.52086846 | 33.28905261 | 1.508373355 | 106.449101 | 17.10609814 | 6.222874447 | 2.637581139 | 0.024158261 | 1.616934336 | 1.768184371 |
| HMDB0059915 | 4-Dodecylbenzenesulfonic Acid | 1110.775967 | 1243.501689 | 1397.525104 | 906.5723372 | 114.7104438 | 665.6322413 | 1250.60092 | 562.3050074 | 2.224061503 | 1.153196684 | 0.050382061 | 1.29772407 | 1.628401759 |
| HMDB0032960 | 1-Octen-3-yl primeveroside | 42.19955553 | 106.0863298 | 130.4080876 | 34.13417728 | 2.323943902 | 10.99624292 | 92.89799095 | 15.81812137 | 5.872883941 | 2.554069128 | 0.051040798 | 1.292082544 | 1.900987012 |
| HMDB0015448 | Troleandomycin | 71.45781139 | 182.5169878 | 231.2645929 | 56.41832816 | 2.519263747 | 36.13737271 | 161.746464 | 31.69165487 | 5.103755695 | 2.351559272 | 0.05942324 | 1.22604367 | 1.746098936 |
| HMDB0000872 | Tetradecanedioic acid | 74.26062868 | 167.3692794 | 3.271407419 | 146.7787643 | 225.781909 | 224.379147 | 81.63377182 | 198.9799401 | 0.410261315 | -1.285384972 | 0.096395879 | 1.01594153 | 1.294496227 |
| HMDB0262697 | PA(10:0/18:1-2OH(9,10)) | 107.2151386 | 42.54025516 | 46.58451621 | 29.36896516 | 23.7964679 | 1.401370753 | 65.44663665 | 18.1889346 | 3.598156686 | 1.847258012 | 0.104657672 | 0.980228929 | 1.570960649 |
| HMDB0038535 | Physagulin C | 22.27904021 | 165.7464085 | 252.0238218 | 17.61842818 | 0.728701293 | 1.114302523 | 146.6830902 | 6.487144 | 22.61135103 | 4.498975291 | 0.105401433 | 0.977153483 | 1.964956535 |
| HMDB0244621 | Cyclo(Tyr-Arg-Gly-Asp-Cys(carboxymethyl)-OH) sulfoxide | 28.37698557 | 43.25405524 | 78.12893368 | 28.62851935 | 15.583229 | 1.50113472 | 49.9199915 | 15.23762769 | 3.276099962 | 1.711979378 | 0.106339277 | 0.973306299 | 1.532297183 |
| HMDB0014681 | Vincristine | 1017.021306 | 1803.414282 | 985.2625405 | 1083.94487 | 259.0759164 | 0.804159916 | 1268.566043 | 447.9416489 | 2.831989493 | 1.501815913 | 0.123875483 | 0.907014638 | 1.338623487 |
| HMDB0304906 | 4-Hydroxyphenylacetic acid sulfate | 1.829513681 | 1.629274485 | 2.006473754 | 2.194241064 | 1.942434164 | 2.09174209 | 1.821753973 | 2.076139106 | 0.877472019 | -0.188574974 | 0.124557073 | 0.904631606 | 1.589476322 |
| HMDB0000207 | Oleic acid | 55.988267 | 38.94272825 | 13.51024171 | 47.61937855 | 98.23657077 | 152.1659854 | 36.14707899 | 99.3406449 | 0.363869985 | -1.458505044 | 0.124689619 | 0.904169704 | 1.605102222 |
| HMDB0015478 | Bambuterol | 38.68444722 | 72.14138614 | 62.83465493 | 28.99627123 | 47.27388538 | 1.557598311 | 57.88682943 | 25.94258497 | 2.231343927 | 1.1579129 | 0.12680699 | 0.896856806 | 1.281688001 |
| HMDB0032549 | N-Undecylbenzenesulfonic acid | 4956.517768 | 4766.468999 | 1467.43739 | 1346.266776 | 388.0861787 | 2247.874367 | 3730.141386 | 1327.409107 | 2.810091753 | 1.490617237 | 0.127741091 | 0.893669377 | 1.647703179 |
| HMDB0304797 | Asn-Arg-Ala-Ile | 72.11220624 | 56.45105151 | 26.70072913 | 41.6254589 | 8.4118946 | 1.248184221 | 51.7546623 | 17.09517924 | 3.027441922 | 1.598099284 | 0.129942805 | 0.886247761 | 1.618103779 |
| HMDB0032823 | Corchorusoside A | 5.920942876 | 435.6954453 | 277.720677 | 3.774568745 | 1.061155178 | 1.039077187 | 239.7790217 | 1.958267037 | 122.4444967 | 6.935984123 | 0.131018486 | 0.882667424 | 1.871000908 |
| HMDB0030148 | Prehumulinic acid | 45.25797563 | 94.32257736 | 2.216501515 | 77.09835026 | 120.7334036 | 112.0171036 | 47.26568484 | 103.2829525 | 0.457632975 | -1.127737084 | 0.132935594 | 0.87635872 | 1.241118753 |
| HMDB0060518 | 4-Carboxymethylenebut-2-en-4-olide | 136.6058921 | 177.8458729 | 181.2574892 | 134.9857394 | 147.7065916 | 127.4677869 | 165.2364181 | 136.7200393 | 1.208574975 | 0.273306974 | 0.13996647 | 0.853975989 | 1.460806693 |
| HMDB0260666 | PE(14:0/PGF1alpha) | 142.696133 | 137.6666527 | 47.06653387 | 89.34498118 | 43.28573321 | 1.327674021 | 109.1431066 | 44.65279613 | 2.444261413 | 1.289398589 | 0.18344457 | 0.73649514 | 1.31227101 |
| HMDB0029095 | Tryptophyl-Tyrosine | 26.08729923 | 1.013656034 | 1.374114849 | 96.40363075 | 177.3267898 | 1.478265153 | 9.491690038 | 91.73622857 | 0.103467193 | -3.272754703 | 0.185438734 | 0.731799547 | 1.15930625 |
| HMDB0253033 | Val-Gly-Val-Ala-Pro-Gly | 323.0149413 | 280.8994042 | 90.9529733 | 189.7453944 | 78.5873902 | 1.202235621 | 231.6224396 | 89.84500674 | 2.578022397 | 1.366264797 | 0.190064641 | 0.72109867 | 1.301078613 |
| HMDB0014586 | Betamethasone | 218.4538337 | 258.4387087 | 192.5480257 | 232.9162552 | 108.6391628 | 1.0564789 | 223.146856 | 114.2039656 | 1.95393264 | 0.966380732 | 0.1929694 | 0.714511553 | 1.215005236 |
| HMDB0000208 | Oxoglutaric acid | 750.3201081 | 284.9634256 | 73.7826543 | 49.81374854 | 156.6989861 | 4.803202722 | 369.6887294 | 70.43864578 | 5.248379284 | 2.391871983 | 0.217865912 | 0.661810715 | 1.505349186 |
| HMDB0304793 | 4-Oxoproline | 1700.748345 | 1990.57381 | 1114.071879 | 1967.364574 | 1785.873071 | 2428.058121 | 1601.798011 | 2060.431922 | 0.77740885 | -0.363254563 | 0.226199077 | 0.645509171 | 1.243920927 |
| HMDB0000258 | Sucrose | 745.3260047 | 645.238263 | 415.4804527 | 586.0544385 | 1515.66713 | 931.4117573 | 602.0149068 | 1011.044442 | 0.59543862 | -0.747975298 | 0.229003884 | 0.640157151 | 1.432464702 |
| HMDB0028941 | Leucyl-Tyrosine | 13.69752372 | 5.862604749 | 5.624443791 | 36.79253687 | 160.7097035 | 34.21951469 | 8.394857418 | 77.24075 | 0.10868454 | -3.201781364 | 0.240514594 | 0.618858567 | 2.061432986 |
| HMDB0031513 | 3-Hydroxynonanoic acid | 3840.367083 | 3619.878673 | 2364.659512 | 1622.04283 | 35460.97877 | 12004.23764 | 3274.968423 | 16362.41975 | 0.20015184 | -2.320833215 | 0.261516362 | 0.582501135 | 1.257830013 |
| HMDB0029051 | Seryltyrosine | 45.4198317 | 7.966145524 | 7.818364155 | 83.52282756 | 70.72006774 | 8.694122425 | 20.40144713 | 54.31233924 | 0.375631899 | -1.412608512 | 0.266390081 | 0.57448195 | 1.002819821 |
| HMDB0002486 | Taurochenodeoxycholate-3-sulfate | 103.5637989 | 27.23824073 | 49.92814118 | 34.42882353 | 44.81114743 | 1.44488234 | 60.24339361 | 26.8949511 | 2.239951781 | 1.163467676 | 0.270992124 | 0.567043331 | 1.200305199 |
| HMDB0029084 | Tryptophylhydroxyproline | 19.66051068 | 32.4523179 | 102.731491 | 25.59454494 | 27.57108256 | 1.139788178 | 51.61477319 | 18.10180522 | 2.851360544 | 1.511650474 | 0.285186298 | 0.544871344 | 1.20154809 |
| HMDB0000888 | Undecanedioic acid | 7.983581334 | 11.08820564 | 12.39166655 | 45.35863389 | 39.7006019 | 2.848539218 | 10.48781784 | 29.30259167 | 0.357914343 | -1.482313736 | 0.293030725 | 0.533086841 | 0.644599233 |
| HMDB0035881 | 11-Hydroxy-9-tridecenoic acid | 54.69888358 | 101.0219039 | 1.384803749 | 1.789999629 | 40.95632331 | 2.6885751 | 52.36853041 | 15.14496601 | 3.457817625 | 1.789861779 | 0.303417496 | 0.51795938 | 0.801942947 |
| HMDB0000044 | Ascorbic acid | 8702.383295 | 1896.33273 | 1144.516312 | 289.0509602 | 1112.320621 | 484.4096478 | 3914.410779 | 628.593743 | 6.227250625 | 2.638595344 | 0.304554495 | 0.516334986 | 1.742336205 |
| HMDB0001142 | FMNH2 | 25.39434688 | 26.94723178 | 17.62105991 | 29.7267665 | 6.287731631 | 1.576834888 | 23.32087952 | 12.53044434 | 1.861137474 | 0.896184625 | 0.304583338 | 0.516293858 | 1.452348533 |
| HMDB0241295 | 3,8-Dihydroxydodecanoylcarnitine | 26.98475654 | 32.22610561 | 1.379352886 | 25.30215427 | 28.51917975 | 46.10588704 | 20.19673835 | 33.30907369 | 0.606343441 | -0.721792909 | 0.31842139 | 0.496997766 | 1.004971187 |
| HMDB0029154 | gamma-Glutamyllysine | 92.50207749 | 5.638544985 | 2.794037317 | 112.6937375 | 396.1131598 | 2.428488516 | 33.6448866 | 170.4117953 | 0.197432851 | -2.340566033 | 0.321157409 | 0.493282054 | 0.801625661 |
| HMDB0062283 | 11-Hydroxy-D4-neuroprostane | 354.6621358 | 83.64095664 | 100.5971376 | 157.9438631 | 48.07546406 | 1.137306854 | 179.63341 | 69.05221134 | 2.6014143 | 1.379296181 | 0.327446168 | 0.484860088 | 1.299639579 |
| HMDB0000243 | Pyruvic acid | 1753.947923 | 1257.598521 | 626.7805361 | 829.0108539 | 1096.670269 | 480.4881404 | 1212.77566 | 802.056421 | 1.512082727 | 0.596537072 | 0.331220985 | 0.479882156 | 1.098064632 |
| HMDB0040615 | 4,5-Dihydrovomifoliol | 25.43438836 | 33.92219837 | 109.5011995 | 39.0874784 | 32.11197587 | 3.750376235 | 56.28592874 | 24.98327684 | 2.252944204 | 1.171811584 | 0.33852684 | 0.470406893 | 1.114549177 |
| HMDB0303682 | Citrusin III | 363.5764526 | 160.4176315 | 93.33386342 | 575.6376709 | 1036.703036 | 1.289851826 | 205.7759825 | 537.876853 | 0.382570808 | -1.3862013 | 0.344810406 | 0.462419637 | 0.358434118 |
| HMDB0013267 | N-Decanoylglycine | 1994.321276 | 173.5199378 | 396.9809194 | 192.9289738 | 234.0270367 | 55.56831842 | 854.940711 | 160.841443 | 5.315425522 | 2.41018519 | 0.349514187 | 0.456535192 | 1.493543352 |
| HMDB0000701 | Hexanoylglycine | 816.496621 | 88.47693168 | 121.0244366 | 46.20166371 | 81.53622471 | 40.83593001 | 341.9993298 | 56.19127281 | 6.086342463 | 2.605575513 | 0.351776758 | 0.453732857 | 1.633481295 |
| HMDB0240259 | Stercobilin | 563.174907 | 16.09032253 | 13.13080576 | 642.6740687 | 1022.759484 | 2.540764677 | 197.4653451 | 555.9914392 | 0.355158967 | -1.493463185 | 0.362783811 | 0.440352101 | 0.390389978 |
| HMDB0013059 | (9-hydroxy-PGA1)-glutathione | 761.8645749 | 54.9960424 | 17.99483608 | 754.95643 | 2134.84856 | 1.271773217 | 278.2851511 | 963.6922544 | 0.288769729 | -1.792008579 | 0.364100795 | 0.438778373 | 0.113979836 |
| HMDB0242130 | 3-(3-Amino-3-carboxypropyl)uridine | 64.9577997 | 4.834922079 | 2.121116225 | 54.89622484 | 308.797581 | 2.054922785 | 23.97127933 | 121.9162429 | 0.196620883 | -2.346511537 | 0.369177627 | 0.432764626 | 0.780496138 |
| HMDB0028823 | Glutamylleucine | 611.6867085 | 46.90142724 | 15.99542537 | 531.6155912 | 2804.859799 | 6.201053541 | 224.861187 | 1114.225481 | 0.20180941 | -2.308934648 | 0.369537946 | 0.43234096 | 0.50889553 |
| HMDB0032958 | 3-Octanol glucoside | 189.227003 | 41.48713004 | 1.291545202 | 17.16656905 | 37.55029895 | 1.824775342 | 77.33522699 | 18.84721445 | 4.103270873 | 2.036774395 | 0.370791288 | 0.430870478 | 0.563263464 |
| HMDB0029140 | Valylvaline | 41.58419169 | 2.378416609 | 2.158609835 | 33.44038613 | 195.3896746 | 2.091246335 | 15.37373938 | 76.97376903 | 0.199726992 | -2.323898774 | 0.371892753 | 0.429582285 | 0.895044215 |
| HMDB0253924 | Tyrosine ethyl ester | 269.7465645 | 15.35470946 | 32.53962518 | 10.3993213 | 27.34895604 | 1.103005193 | 105.8802997 | 12.95042751 | 8.175815017 | 3.031362555 | 0.37498801 | 0.425982618 | 1.467659019 |
| HMDB0000832 | Capryloylglycine | 1650.884245 | 72.47099998 | 127.9690555 | 33.27112178 | 65.10012317 | 2.365231918 | 617.1081002 | 33.57882562 | 18.37789407 | 4.199899552 | 0.376301298 | 0.424464283 | 1.647989562 |
| HMDB0013286 | N-Undecanoylglycine | 5165.689064 | 1485.244098 | 475.2268799 | 829.7456496 | 1414.547334 | 602.0130027 | 2375.386681 | 948.7686621 | 2.503652129 | 1.32403412 | 0.37960195 | 0.420671565 | 0.902173074 |
| HMDB0253144 | Hexaminolevulinate | 1901.81085 | 102.7757576 | 191.4566181 | 82.09534368 | 138.1513139 | 27.50314986 | 732.0144086 | 82.58326913 | 8.863955329 | 3.147950611 | 0.382817877 | 0.417007789 | 1.499109255 |
| HMDB0000682 | Indoxyl sulfate | 286.8084625 | 189.5114823 | 62.78284632 | 208.0104992 | 70.55978325 | 3.300759929 | 179.7009304 | 93.95701414 | 1.912586644 | 0.935525107 | 0.387565871 | 0.411654474 | 1.181010696 |
| HMDB0001898 | Mesobilirubinogen | 56.4258762 | 1.674075514 | 2.69703617 | 38.55630974 | 206.6264114 | 3.517031534 | 20.26566263 | 82.89991755 | 0.244459382 | -2.032333319 | 0.391397515 | 0.407381937 | 1.000841272 |
| HMDB0060104 | Prostaglandin C1 | 1.453287524 | 7.382094601 | 1.729107457 | 1.699589338 | 17.528187 | 226.8938513 | 3.521496527 | 82.04054253 | 0.042923857 | -4.542076467 | 0.392368775 | 0.406305562 | 1.345003876 |
| HMDB0242403 | Chenodeoxycholylmethionine | 625.7303816 | 14.48421274 | 19.78217151 | 655.6720742 | 1065.904746 | 1.455695888 | 219.9989219 | 574.3441719 | 0.383043709 | -1.384419067 | 0.392970318 | 0.405640252 | 0.230682521 |
| HMDB0039051 | Notoginsenoside R10 | 439.4359199 | 123.9414505 | 85.12170682 | 462.5528475 | 1461.000821 | 1.177203467 | 216.166359 | 641.5769573 | 0.336929743 | -1.569480307 | 0.393397941 | 0.405167918 | 0.304914479 |
| HMDB0250680 | Cyclo(Arg-Gly-AspPhe-Val) | 85.82682496 | 11.23046294 | 2.716901142 | 68.38193565 | 288.909438 | 1.184227086 | 33.25806301 | 119.4918669 | 0.278329094 | -1.845136374 | 0.396144965 | 0.402145859 | 0.402045465 |
| HMDB0015520 | Oxprenolol | 345.9869588 | 74.21457556 | 1.267550373 | 43.73807578 | 73.44123248 | 1.790873955 | 140.4896949 | 39.65672741 | 3.542644694 | 1.824826779 | 0.39905867 | 0.398963249 | 0.422939537 |

## *Supplementary Table 1*
## Negative Mode

| HMDB_ID | Compound_name | DB]a,l]P-#1 | DB]a,l]P-#2 | DB]a,l]P-#3 | DMSO-#1 | DMSO-#2 | DMSO-#3 | AVE (DB[a,l]P) | AVE (DMSO) | FC (DB[a,l]P /DMSO) | Log2(FC) | T-Test | Log10 (P_value) | VIP |
|---|---|---|---|---|---|---|---|---|---|---|---|---|---|---|
| HMDB0028940 | Leucyl-Tryptophan | 16.03640108 | 7.093397375 | 7.292656936 | 24.01038816 | 112.877086 | 1.354147268 | 10.14081846 | 46.08054049 | 0.220067264 | -2.183983542 | 0.401719173 | 0.39607744 | 0.463144939 |
| HMDB0255727 | Norcholic acid | 1675.186823 | 263.3653017 | 243.9468612 | 1828.130206 | 9443.330656 | 1.209579686 | 727.499662 | 3757.556814 | 0.193609757 | -2.368776435 | 0.40496122 | 0.392586564 | 0.223640025 |
| HMDB0011561 | MG(14:0/0:0/0:0) | 243.1368703 | 666.6135839 | 13.70008879 | 167.1795449 | 6588.030764 | 613.1223999 | 307.8168477 | 2456.110903 | 0.125326933 | -2.996231604 | 0.408433232 | 0.388878929 | 1.238850216 |
| HMDB0060769 | 4-Hydroxyphenytoin glucuronide | 2.207379046 | 1.809915107 | 2.371759076 | 2.244945717 | 14.43035275 | 2.290939851 | 2.129684409 | 6.322079439 | 0.336864544 | -1.569759505 | 0.409760097 | 0.387470336 | 1.260139211 |
| HMDB0252831 | Glycyl-prolyl-arginyl-valyl-valyl-glutamic acid | 121.2509595 | 5.073103025 | 5.540374713 | 103.9230549 | 638.6551967 | 1.589766172 | 43.95481242 | 248.0560059 | 0.17719713 | -2.496572855 | 0.410719001 | 0.386455205 | 0.61155457 |
| HMDB0004160 | Urobilin | 36.02609587 | 1.847324626 | 2.482728344 | 29.13660821 | 181.1890996 | 2.116113492 | 13.45204961 | 70.81394043 | 0.189963297 | -2.396207392 | 0.412368247 | 0.384714783 | 0.931835454 |
| HMDB0247339 | 3-(Cyclohexylamino)-2-hydroxy-1-propanesulfonic acid | 68.59405612 | 58.86450983 | 114.375661 | 71.87317545 | 83.77062649 | 1.917228379 | 80.61140897 | 52.52034344 | 1.53486066 | 0.618107688 | 0.412487537 | 0.384589168 | 1.012226178 |
| HMDB0028980 | Methionyl-Phenylalanine | 23.7323693 | 10.81122903 | 8.27872692 | 32.13227646 | 320.0813126 | 1.35373989 | 14.27410842 | 117.8557763 | 0.121115051 | -3.045549929 | 0.414867052 | 0.382091055 | 0.487583545 |
| HMDB0029704 | 1-(2,6,6-Trimethyl-2-cyclohexen-1-yl)-1,6-heptadien-3-one | 6.760892885 | 2.896760925 | 5.149495057 | 3.6795622 | 2930.266357 | 39.42859923 | 4.935716289 | 991.1248394 | 0.004979914 | -7.649663508 | 0.416130703 | 0.38077024 | 1.478591985 |
| HMDB0001043 | Arachidonic acid | 23.82547899 | 44.09625079 | 1.423455143 | 34.0917933 | 322.4253755 | 14.46205449 | 23.11506164 | 123.6597411 | 0.186924713 | -2.419470775 | 0.419032385 | 0.377752411 | 1.129784317 |
| HMDB0010382 | LysoPC(16:0/0:0) | 22.44271921 | 27.19393839 | 4.090895047 | 28.64581268 | 1034.673474 | 13.42745849 | 17.90918422 | 358.9155818 | 0.04989804 | -4.32487303 | 0.419140823 | 0.377640038 | 1.22843078 |
| HMDB0260499 | MG(20:4-2OH/0:0/0:0) | 3.045217901 | 2.702268834 | 4.670272656 | 3.325152295 | 922.5065634 | 9.136499018 | 3.472586463 | 311.6560716 | 0.011142367 | -6.487800393 | 0.419195281 | 0.377583615 | 1.389361248 |
| HMDB0251779 | Succinic acid, sulfo-, 1,4-dioctyl ester | 31.68486171 | 65.75210912 | 5.236707044 | 20.59882882 | 6385.887291 | 74.56159037 | 34.22455929 | 2160.349237 | 0.015842142 | -5.980088779 | 0.420235548 | 0.376507213 | 1.294151 |
| HMDB0036690 | 3-Epinobilin | 3.647562406 | 3.086852521 | 5.487415845 | 3.921022875 | 426.524415 | 5.379396521 | 4.073943591 | 145.2749448 | 0.028042988 | -5.156216092 | 0.421077714 | 0.375637744 | 1.259503844 |
| HMDB0034086 | Dolicholide | 1.65629903 | 1.40940181 | 2.621046242 | 1.824705359 | 262.6664542 | 2.48982184 | 1.895582361 | 88.99366045 | 0.021300195 | -5.552989522 | 0.421492738 | 0.375209903 | 1.256716 |
| HMDB0000467 | Nutriacholic acid | 1.599885267 | 1.454712035 | 2.840605769 | 1.86348977 | 281.4373013 | 2.56584888 | 1.96506769 | 95.28887999 | 0.020622214 | -5.59965695 | 0.421620089 | 0.375078705 | 1.256718468 |
| HMDB0005886 | 19-Noretiocholanolone | 2.818935281 | 2.501469912 | 4.35284921 | 3.199673287 | 615.6263607 | 4.2075266 | 3.224418134 | 207.6778535 | 0.015526057 | -6.009164726 | 0.42174706 | 0.374947936 | 1.255219712 |
| HMDB0029115 | Tyrosyl-Threonine | 71.99654768 | 5.666613973 | 2.095034809 | 54.37146273 | 336.4130509 | 2.029655289 | 26.58606549 | 130.9380563 | 0.203043074 | -2.300142277 | 0.42193791 | 0.374751453 | 0.734618366 |
| HMDB0034750 | Isopentyl betaglucoside | 2.063336943 | 1.478222036 | 1.919211417 | 1.919800318 | 467.8122531 | 2.151799932 | 1.820256759 | 157.2946178 | 0.011572276 | -6.433183498 | 0.422115802 | 0.374568389 | 1.226709617 |
| HMDB0302324 | Bioside | 16801.34302 | 3395.303121 | 1757.134848 | 20471.67517 | 26369.7263 | 1.023654215 | 7317.926998 | 15614.14171 | 0.468673023 | -1.093346339 | 0.422916295 | 0.373745582 | 0.597877972 |
| HMDB0029131 | Valylleucine | 36.47994494 | 17.26452153 | 14.24348839 | 44.05787827 | 251.8470677 | 2.137286786 | 22.66265162 | 99.34741092 | 0.228115171 | -2.132165698 | 0.425375967 | 0.37122705 | 0.297700245 |
| HMDB0010320 | Cortolone-3-glucuronide | 58.62429101 | 0.941712992 | 1.160618396 | 37.91853979 | 486.6218876 | 1.252600482 | 20.24220747 | 175.2643426 | 0.115495298 | -3.114093978 | 0.425430915 | 0.371170955 | 0.945744094 |
| HMDB0041316 | Cinnamyl butyrate | 235.1605463 | 129.7922793 | 1.427449581 | 89.44857865 | 81.03182099 | 2.219266352 | 122.1267584 | 57.56655533 | 2.121488035 | 1.085076542 | 0.426843196 | 0.369731636 | 0.293671449 |
| HMDB0000751 | Xylulose | 270.1072476 | 188.8370843 | 146.7431498 | 350.9684926 | 597.4374949 | 70.931343 | 201.8958272 | 339.7791102 | 0.594197292 | -0.750986064 | 0.427623476 | 0.368938461 | 0.399271459 |
| HMDB0033373 | 2-Ethyl-1-hexanol sulfate | 8.161586271 | 11.93117454 | 1.173258455 | 8.900456863 | 430.4340905 | 1.560089752 | 7.088673088 | 146.964879 | 0.04823379 | -4.37381202 | 0.427779895 | 0.368779631 | 0.857661944 |
| HMDB0005096 | N-Arachidonoylglycine | 126.7929118 | 66.85759509 | 46.95316589 | 156.4020473 | 1033.357495 | 1.212559263 | 80.20122425 | 396.9907006 | 0.202022929 | -2.307409049 | 0.428027463 | 0.368345767 | 0.110321504 |
| HMDB0031181 | Homodihydrojasmone | 248.4790643 | 78.44273867 | 26.97506145 | 52.99492021 | 66.14959822 | 36.73373597 | 117.9656215 | 51.95941813 | 2.270341465 | 1.182909299 | 0.428287576 | 0.368264523 | 0.885257845 |
| HMDB0304536 | alphaxylose 1-phosphate | 1436.772451 | 2147.942171 | 2350.96104 | 2223.538552 | 1660.756944 | 630.4880744 | 1978.558554 | 1504.927857 | 1.314719869 | 0.394755433 | 0.431960217 | 0.364556249 | 0.97143086 |
| HMDB0011562 | MG(14:1/0:0/0:0) | 75.94593178 | 193.2808573 | 48.38571468 | 43.92631678 | 1317.634184 | 147.3593297 | 105.8708546 | 502.9734867 | 0.210489891 | -2.248177144 | 0.433704121 | 0.362806451 | 0.96695372 |
| HMDB0010380 | LysoPC(14:1/0:0) | 81.46231295 | 1.22295119 | 1.684978601 | 47.10890482 | 497.2196837 | 1.783664892 | 28.12341425 | 182.0374178 | 0.154492492 | -2.69439137 | 0.433745851 | 0.362764667 | 0.916367957 |
| HMDB0303182 | Annomuricatin B | 149.1454328 | 29.21879521 | 38.2119645 | 122.2000305 | 483.4710952 | 1.311828917 | 72.19206418 | 202.3276515 | 0.356807701 | -1.486781342 | 0.434252256 | 0.362257917 | 0.152738408 |
| HMDB0036968 | Araliasaponin II | 238.3668311 | 67.13150613 | 51.84212132 | 226.1857582 | 629.5451735 | 1.277389422 | 119.1134862 | 285.6694404 | 0.416962648 | -1.262009945 | 0.437404675 | 0.359116579 | 0.301992737 |
| HMDB0011635 | p-Cresol sulfate | 286.4956257 | 114.0733977 | 83.140301 | 181.223719 | 84.84893431 | 11.55680936 | 161.2364415 | 92.54315424 | 1.742283833 | 0.80097967 | 0.439458467 | 0.357082163 | 1.137416308 |
| HMDB0041623 | N6-Carbamoylthreonyladenosine | 18.01435266 | 3.476430137 | 1.396467487 | 8.007615451 | 185.7233673 | 1.580527224 | 7.629083429 | 65.10383667 | 0.117183315 | -3.093160922 | 0.441518496 | 0.355051099 | 0.795695602 |
| HMDB0266576 | PA(PGE1/2:0) | 12.26410254 | 1.062498618 | 1.350014344 | 5.801231643 | 68.16579334 | 1.595629405 | 4.892205169 | 25.18755146 | 0.194231074 | -2.364154067 | 0.446091566 | 0.350575988 | 0.969928811 |
| HMDB0028868 | Hydroxyprolyl-Lysine | 236.5691661 | 622.6520051 | 571.4784172 | 413.0260784 | 491.1154087 | 91.15236167 | 476.8998628 | 331.7646163 | 1.437464514 | 0.523526341 | 0.446674874 | 0.350004976 | 0.823044197 |
| HMDB0028738 | Asparaginyl-Phenylalanine | 857.5258135 | 3201.948047 | 4647.170896 | 2825.986561 | 2035.107664 | 606.4336913 | 2902.214919 | 1822.509305 | 1.592428039 | 0.671228179 | 0.446770879 | 0.349915143 | 0.628947432 |
| HMDB0259425 | Candidin | 139.028048 | 44.68457818 | 61.77524089 | 131.3502103 | 917.628413 | 1.307523578 | 81.82928903 | 350.0953823 | 0.233734271 | -2.097058811 | 0.447842518 | 0.348874677 | 0.143731197 |
| HMDB0013272 | N-Lauroylglycine | 438.916593 | 17.60505222 | 1.969756216 | 16.51452032 | 41.35572051 | 2.0975069 | 152.8304671 | 19.98924924 | 7.645633174 | 2.934635983 | 0.45144769 | 0.345392565 | 0.588601476 |
| HMDB0266244 | PA(20:5-3OH(5,6,15)/22:6) | 146.4776622 | 6.20196517 | 14.65134308 | 77.76578482 | 730.3100009 | 1.437896205 | 55.77699016 | 269.837894 | 0.20670555 | -2.274350973 | 0.45369466 | 0.343236332 | 0.351011552 |
| HMDB0245262 | 2-Nitrophenyl octyl ether | 149.582232 | 0.865750494 | 0.900352584 | 0.8648902 | 12.03164424 | 1.372412916 | 50.44944502 | 4.756315785 | 10.60683254 | 3.406921991 | 0.454061748 | 0.342885083 | 0.458676161 |
| HMDB0029109 | Tyrosyl-Leucine | 14.70273491 | 16.2211894 | 5.405695383 | 17.16201345 | 105.6126622 | 1.736951638 | 12.10987323 | 41.50387577 | 0.291776925 | -1.777062304 | 0.459922358 | 0.337315478 | 0.316842306 |
| HMDB0028874 | Hydroxyprolyl-Tryptophan | 500.9109414 | 307.4199514 | 220.6954428 | 884.788277 | 896.7679968 | 1.255437194 | 343.0087785 | 594.2705703 | 0.577192941 | -0.792874438 | 0.460248577 | 0.337007546 | 0.618197245 |
| HMDB0030951 | 2,4-Decadienoic isobutylamide | 513.845905 | 2.402814173 | 2.94237372 | 2.899768943 | 43.09038079 | 10.74065918 | 173.0636976 | 18.91026964 | 9.151836591 | 3.194061292 | 0.461260663 | 0.336053581 | 0.234096849 |
| HMDB0015125 | Exemestane | 2502.950749 | 1511.523376 | 1037.787575 | 4324.55363 | 4448.154888 | 1.25482804 | 1684.087233 | 2924.654449 | 0.575824345 | -0.796299309 | 0.461458577 | 0.335867277 | 0.705888538 |
| HMDB0000159 | Phenylalanine | 4634.055412 | 897.9855866 | 752.4608458 | 4423.369068 | 5279.44515 | 1019.292101 | 2094.833948 | 3574.03544 | 0.586125679 | -0.77071805 | 0.461590719 | 0.335742932 | 0.858620477 |
| HMDB0035701 | Cucurbitacin I 2-glucoside | 30.83238609 | 1.462357588 | 1.809966629 | 8.819861315 | 190.2657927 | 1.886884078 | 11.36823677 | 66.99084603 | 0.169698361 | -2.558955464 | 0.463118093 | 0.334308252 | 0.822987667 |
| HMDB0244560 | Desmethylmisonidazole | 1418.595516 | 265.722685 | 235.416561 | 1366.784384 | 1583.110229 | 312.0108892 | 639.9115873 | 1087.301834 | 0.588531691 | -0.764807991 | 0.463891272 | 0.333583798 | 0.859342047 |
| HMDB0241996 | N-Arachidonoyl Histidine | 457.3119895 | 111.8745574 | 64.51399442 | 379.8718665 | 1056.395423 | 2.991133713 | 211.2335138 | 479.7528076 | 0.440296566 | -1.183452502 | 0.464120758 | 0.333369007 | 0.220742013 |
| HMDB0116154 | CDP-DG(i-12:0/a-17:0) | 17.95229988 | 4.839775515 | 8.263746173 | 12.8898341 | 105.4399664 | 1.209691649 | 10.35194059 | 39.84649739 | 0.259795495 | -1.94455168 | 0.465859001 | 0.331745509 | 0.292302968 |
| HMDB0059978 | 4-Hydroxy-5-(dihydroxyphenyl)-valeric acid-O-sulphate | 2.677468903 | 2.139873201 | 2.889348047 | 2.442362568 | 2.913995259 | 3.042999758 | 2.568896717 | 2.799785862 | 0.917533284 | -0.124167601 | 0.467990789 | 0.329762695 | 0.964109754 |
| HMDB0251874 | Epothilone B | 194.0974671 | 48.78793952 | 63.53829319 | 108.2137786 | 63.05640629 | 1.819755669 | 102.1412333 | 57.69664685 | 1.770314894 | 0.824006002 | 0.468269941 | 0.32950372 | 1.0567494 |
| HMDB0255446 | 2-Naphthalenesulfonic acid | 7.585191356 | 161.4625702 | 44.7165577 | 64.37998845 | 28.49451288 | 1.728161435 | 71.25477309 | 31.53422092 | 2.259601506 | 1.176068368 | 0.46965547 | 0.328220615 | 0.730103677 |
| HMDB0036603 | Pteroside D | 76.43051724 | 14.3600667 | 19.60506433 | 59.6536281 | 193.0230112 | 1.21562118 | 36.79854942 | 84.6307535 | 0.434812972 | -1.201533114 | 0.470936756 | 0.327037413 | 0.100827697 |
| HMDB0303680 | Citrusin I | 6.344983961 | 27.41338862 | 17.1567556 | 3.289814411 | 23.76635279 | 1.670162683 | 16.97170939 | 9.575443293 | 1.772420229 | 0.825720698 | 0.47350945 | 0.324671349 | 1.03915439 |
| HMDB0256445 | Phenyl dihydrogen phosphate | 497.8149245 | 577.4505845 | 959.0730915 | 764.1812886 | 670.1983221 | 1017.444888 | 678.1128668 | 817.2748328 | 0.829724396 | -0.269295889 | 0.47363053 | 0.324560311 | 1.041768468 |
| HMDB0010386 | LysoPC(18:2/0:0) | 35.42893401 | 59.19979101 | 7.586345465 | 79.42925255 | 14.40305145 | 67.24097211 | 34.07169016 | 53.69109204 | 0.634587394 | -0.656109233 | 0.475043906 | 0.323266249 | 0.543377753 |
| HMDB0031876 | Prenyl glucoside | 5.216721443 | 16.73086195 | 4.822430207 | 12.91555582 | 39.49376394 | 2.146006847 | 8.923337865 | 18.18510887 | 0.490694773 | -1.027102192 | 0.475196986 | 0.323126323 | 0.441962035 |
| HMDB0000023 | 3-Hydroxyisobutyric acid | 290.5819689 | 216.1851664 | 908.266621 | 264.4795551 | 298.7678088 | 285.9230126 | 471.6779188 | 283.0567922 | 1.666372021 | 0.736710521 | 0.48052469 | 0.318284293 | 0.664917443 |
| HMDB0032365 | Maltyl isobutyrate | 17.43714247 | 34.77121843 | 32.46764677 | 59.99189362 | 78.05598103 | 1.261344095 | 28.22533589 | 46.43640625 | 0.607827741 | -0.718265576 | 0.486997229 | 0.31247351 | 0.31788931 |
| HMDB0242172 | Decadienedioic acid | 18.27197978 | 15.64749096 | 49.44815444 | 31.77240108 | 199.2898966 | 5.728528249 | 27.78920839 | 78.93027531 | 0.352072868 | -1.506054043 | 0.489234917 | 0.310482555 | 0.450995747 |
| HMDB0028735 | Asparaginyl-Leucine | 568.0800922 | 75.06072749 | 100.8371001 | 208.2772488 | 2717.590012 | 15.56906839 | 247.9926399 | 980.4787763 | 0.252930146 | -1.983189098 | 0.490067111 | 0.309744443 | 0.208454754 |
| HMDB0002655 | Isorhamnetin | 26.35219347 | 39.71794752 | 75.52945299 | 62.84821841 | 24.76164942 | 1.191184836 | 47.19986466 | 29.60035089 | 1.59457112 | 0.673168445 | 0.49031164 | 0.309527796 | 1.059678026 |
| HMDB0000687 | Leucine | 2196.377277 | 392.8184313 | 320.7836023 | 1690.182688 | 2714.189524 | 491.7031117 | 969.9931035 | 1632.025108 | 0.594349375 | -0.750616857 | 0.497467696 | 0.303235116 | 0.882826665 |

# *Supplementary Table 1*
## Negative Mode

| HMDB_ID | Compound_name | DB]a,l]P-#1 | DB]a,l]P-#2 | DB]a,l]P-#3 | DMSO-#1 | DMSO-#2 | DMSO-#3 | AVE (DB[a,l]P) | AVE (DMSO) | FC (DB[a,l]P /DMSO) | Log2(FC) | T-Test | Log10 (P_value) | VIP |
|---|---|---|---|---|---|---|---|---|---|---|---|---|---|---|
| HMDB0032616 | Sinapic acid | 32.54161446 | 48.25941647 | 70.66299818 | 40.08717874 | 522.450027 | 1.845889632 | 50.4880097 | 188.1276984 | 0.268370953 | -1.897699565 | 0.497831588 | 0.30291755 | 0.155344023 |
| HMDB0002183 | Docosahexaenoic acid | 7.371151808 | 25.16321867 | 1.491542894 | 11.55047365 | 74.04590578 | 1.404608801 | 11.34197112 | 29.00032941 | 0.39109801 | -1.354397899 | 0.499338493 | 0.301604954 | 0.417416453 |
| HMDB0031573 | Sulforaphene | 12621.18068 | 16296.40971 | 10161.05774 | 13143.07934 | 12462.42973 | 20048.22376 | 13026.21604 | 15217.91094 | 0.855979253 | -0.224352265 | 0.506631059 | 0.295308189 | 0.759962924 |
| HMDB0266683 | PA(8:0/PGF1alpha) | 22.59806438 | 8.185770563 | 11.6824439 | 11.31603757 | 120.3529269 | 1.90178766 | 14.15542628 | 44.52358404 | 0.31793097 | -1.653214535 | 0.508718206 | 0.293522719 | 0.129455035 |
| HMDB0240271 | Ergoloid | 108.0664954 | 2.489483351 | 1.214300143 | 110.2893315 | 108.6618583 | 1.509830683 | 37.25675963 | 73.48700682 | 0.506984312 | -0.979986988 | 0.512679587 | 0.290153974 | 0.660932661 |
| HMDB0015387 | Aliskiren | 146.1949521 | 66.20597803 | 85.06876776 | 74.19890556 | 814.5537229 | 2.029111769 | 99.15656597 | 296.9272467 | 0.333942294 | -1.582329271 | 0.526223443 | 0.278829808 | 0.362159741 |
| HMDB0013317 | Tridecanoylglycine | 716.6809288 | 44.48967597 | 1.98773473 | 30.10717983 | 60.16469129 | 143.6322142 | 254.3861132 | 77.96802843 | 3.262697779 | 1.706065358 | 0.526658132 | 0.278471206 | 0.200828263 |
| HMDB0001984 | Finasteride | 128.6586351 | 41.72370102 | 54.90016151 | 56.55432198 | 89.86106378 | 1.245896614 | 75.09416587 | 49.22042746 | 1.525670738 | 0.609443641 | 0.527231487 | 0.277998661 | 0.980366503 |
| HMDB0060732 | 25-O-Desacetyl rifabutin | 4377.003037 | 1585.256156 | 511.318017 | 5098.806194 | 5882.931442 | 1.025519244 | 2157.85907 | 3660.921052 | 0.589430649 | -0.762606017 | 0.527397373 | 0.277862038 | 0.636032917 |
| HMDB0000796 | N-Acetyl-4-O-acetylneuraminic acid | 26.12733696 | 26.00663854 | 55.08590502 | 66.62417052 | 101.6748158 | 1.359388841 | 35.73996017 | 56.55279171 | 0.63197517 | -0.662060217 | 0.538058399 | 0.269170585 | 0.351801629 |
| HMDB0242380 | Cholylvaline | 2020.624314 | 253.1515124 | 380.9105793 | 1286.001841 | 3992.660464 | 6.103579005 | 884.8954684 | 1761.588628 | 0.5023281 | -0.993298114 | 0.538716126 | 0.268640024 | 0.340325364 |
| HMDB0014469 | Fluorometholone | 26.46970011 | 3.439157002 | 1.345280087 | 10.51815479 | 54.18894287 | 1.935247049 | 10.41804573 | 22.2141149 | 0.468983157 | -1.092391984 | 0.549498324 | 0.260033628 | 0.606268738 |
| HMDB0304807 | Phe-Phe-Pro-Arg | 398.2836837 | 51.49399531 | 72.22894826 | 253.8727759 | 745.4899642 | 1.648378264 | 174.0022091 | 333.6703728 | 0.52147935 | -0.93931797 | 0.551043872 | 0.258813823 | 0.309548012 |
| HMDB0000943 | Threonic acid | 17430.76069 | 5572.490208 | 7658.865767 | 7695.122975 | 5173.50864 | 41264.80535 | 10220.70555 | 18044.47899 | 0.566417327 | -0.820062696 | 0.55603532 | 0.254897621 | 0.35366926 |
| HMDB0013302 | Phenylalanylphenylalanine | 147.4956064 | 135.1364686 | 319.4809841 | 372.8525286 | 565.1873858 | 1.34942723 | 200.704353 | 313.1297805 | 0.6409622 | -0.641688817 | 0.557393653 | 0.253837981 | 0.571716479 |
| HMDB0000115 | Glycolic acid | 1464.724659 | 811.7218623 | 1025.286655 | 919.7688284 | 844.1489653 | 3037.765063 | 1100.577725 | 1600.560952 | 0.687620002 | -0.540316583 | 0.563238967 | 0.249307306 | 0.521428458 |
| HMDB0249109 | beta-CCM | 132.9148641 | 23.35499968 | 30.26453376 | 91.83573125 | 214.8081433 | 10.01810918 | 62.1781325 | 105.5539946 | 0.589064703 | -0.763501985 | 0.565094928 | 0.24787859 | 0.256660708 |
| HMDB0000714 | Hippuric acid | 13.66175723 | 12.02556125 | 7.123150579 | 10.65664979 | 40.04982507 | 4.083028363 | 10.93682302 | 18.26316774 | 0.598845894 | -0.739743304 | 0.577631478 | 0.238349148 | 0.278654103 |
| HMDB0251960 | estradiol alone | 63.97932414 | 81.06013271 | 1.371873127 | 85.74027801 | 44.67316005 | 63.91917918 | 48.80377666 | 64.77753908 | 0.753405846 | -0.408500866 | 0.585547935 | 0.232437546 | 0.818252118 |
| HMDB0001858 | p-Cresol | 35.40738168 | 15.76690435 | 12.21813939 | 27.72291324 | 14.84595363 | 2.83423204 | 21.13080847 | 15.1343663 | 1.396213627 | 0.481519698 | 0.587515461 | 0.2309807 | 0.94068168 |
| HMDB0001127 | 6-Phosphonogluconolactone | 307.1975569 | 109.0380682 | 358.369587 | 381.4367665 | 325.4659792 | 221.0842611 | 258.2017374 | 309.3290023 | 0.834715579 | -0.260643397 | 0.597913777 | 0.22336144 | 0.748950733 |
| HMDB0040198 | 2,5-Dimethyl-3-(methylthio)furan | 12891.59864 | 16929.45217 | 10278.41792 | 15738.12469 | 14174.11425 | 13668.33626 | 13366.48958 | 14526.8584 | 0.920122521 | -0.120102116 | 0.598557553 | 0.222865063 | 0.661430835 |
| HMDB0000296 | Uridine | 268.4830908 | 180.0121351 | 141.9083737 | 324.7290018 | 492.3569819 | 17.9411847 | 196.8011998 | 278.3423895 | 0.707047174 | -0.500121621 | 0.601167737 | 0.221004335 | 0.323040157 |
| HMDB0000448 | Adipic acid | 67.94733518 | 152.965369 | 157.9947696 | 162.9576318 | 64.35762474 | 84.08040713 | 126.3024912 | 103.7985545 | 1.216803951 | 0.283096742 | 0.620190703 | 0.207474748 | 0.632742474 |
| HMDB0260477 | MG(6 keto-PGF1alpha/0:0/0:0) | 19.84272809 | 26.4624384 | 17.20461692 | 5.782330551 | 125.801212 | 1.628584984 | 21.1699278 | 44.40404253 | 0.476756768 | -1.068674676 | 0.626089695 | 0.203363444 | 0.483706365 |
| HMDB0011686 | p-Cresol glucuronide | 54.7442884 | 24.13078768 | 13.23380993 | 33.61620876 | 31.66794106 | 1.449618005 | 30.702962 | 22.24458928 | 1.38024405 | 0.464923382 | 0.629402991 | 0.201071198 | 0.845638999 |
| HMDB0000254 | Succinic acid | 1833.105451 | 2258.160873 | 2400.56596 | 2204.609076 | 2209.708323 | 1704.71377 | 2163.944095 | 2039.677057 | 1.06092486 | 0.08532248 | 0.630506238 | 0.200310612 | 0.486989524 |
| HMDB0029007 | Phenylalanyltyrosine | 19.32964053 | 33.71147302 | 22.11775711 | 29.99960241 | 79.0471154 | 1.484017769 | 25.05295688 | 36.84357853 | 0.679981638 | -0.556432306 | 0.636276865 | 0.196353867 | 0.405281827 |
| HMDB0029610 | Ascladiol | 9752.986002 | 15178.28628 | 9152.414083 | 10819.39118 | 10327.9579 | 17471.32968 | 11361.22879 | 12872.89292 | 0.882569975 | -0.180217426 | 0.640449535 | 0.193515085 | 0.572594905 |
| HMDB0000821 | Phenylacetylglycine | 59.3357109 | 37.08253586 | 15.10128633 | 46.88436251 | 36.03482098 | 1.824179987 | 37.1731777 | 28.24778783 | 1.315967747 | 0.39612413 | 0.657045608 | 0.182404483 | 0.839082188 |
| HMDB0000289 | Uric acid | 572.4574287 | 646.5182262 | 299.9160235 | 603.8653521 | 1208.311166 | 164.0506368 | 506.2972261 | 658.742385 | 0.768581524 | -0.379729799 | 0.659153942 | 0.181013147 | 0.058289466 |
| HMDB0000139 | Glyceric acid | 4353.672358 | 4244.730466 | 10499.41616 | 5361.743422 | 5789.345436 | 4755.123301 | 6365.939661 | 5302.07072 | 1.200651594 | 0.263817569 | 0.659327245 | 0.180898978 | 0.222008351 |
| HMDB0250384 | 6-Cyano-7-nitroquinoxaline-2,3-dione | 7.407213986 | 12.50728752 | 5.552370333 | 10.91527083 | 7.856723349 | 2.23931997 | 8.488957279 | 7.003771383 | 1.212055165 | 0.277455363 | 0.674438049 | 0.171057937 | 0.73357261 |
| HMDB0000893 | Suberic acid | 102.4809437 | 116.775884 | 127.6588526 | 175.1299093 | 182.3252139 | 50.87848473 | 115.6385601 | 136.1112026 | 0.84958885 | -0.235163263 | 0.680548161 | 0.167141135 | 0.07987441 |
| HMDB0242025 | N-Docosahexaenoyl Valine | 31.31450944 | 22.74539886 | 16.14165072 | 55.54337456 | 18.38006076 | 13.88534587 | 23.40051968 | 29.26959373 | 0.799482217 | -0.322862152 | 0.694812077 | 0.158132641 | 0.003590681 |
| HMDB0060649 | Ascorbic acid 2-sulfate | 76.48691933 | 82.1566506 | 102.3534746 | 61.98814257 | 151.8421774 | 82.13563229 | 86.99901485 | 98.65531742 | 0.881848208 | -0.181397748 | 0.701846831 | 0.153757656 | 0.451831002 |
| HMDB0015316 | Fluoxymesterone | 137.5882381 | 52.47601769 | 95.23247209 | 106.8943881 | 274.3564345 | 5.086309949 | 95.09890929 | 128.7790442 | 0.738465718 | -0.437397147 | 0.703174043 | 0.152937169 | 0.446744542 |
| HMDB0000195 | Inosine | 654.6432056 | 365.6865375 | 324.032655 | 723.6347636 | 925.875071 | 40.17106476 | 448.1207994 | 563.2269665 | 0.795630938 | -0.329828719 | 0.709274563 | 0.149185615 | 0.4097184 |
| HMDB0253400 | Ilmofosine | 6.677098131 | 54.96904723 | 46.15488621 | 47.7303997 | 34.9295133 | 1.364546427 | 35.93367719 | 28.00815314 | 1.282972033 | 0.359489722 | 0.715963165 | 0.145109321 | 0.532209605 |
| HMDB0030406 | Homomethionine | 477.1522843 | 447.4341883 | 483.8332121 | 453.3935398 | 391.6054552 | 668.3776428 | 469.4732282 | 504.4588793 | 0.93064717 | -0.103693783 | 0.718176304 | 0.143768929 | 0.293012587 |
| HMDB0000661 | Glutaric acid | 564.5722567 | 258.7582398 | 148.7773201 | 303.6017439 | 384.9574109 | 433.5141561 | 324.0359389 | 374.024437 | 0.866349647 | -0.206978701 | 0.720224992 | 0.142531812 | 0.682558947 |
| HMDB0258586 | Sulfisomidine | 255.0604996 | 418.8325979 | 238.0439644 | 257.6698545 | 245.0970327 | 536.8048503 | 303.9790206 | 346.5239125 | 0.877223792 | -0.188983153 | 0.721705551 | 0.141639954 | 0.339386521 |
| HMDB0014964 | Natamycin | 173.108897 | 152.8960399 | 58.16229362 | 193.7358721 | 113.8545234 | 3.438058472 | 128.0557435 | 103.6761513 | 1.235151401 | 0.304687893 | 0.728879694 | 0.137344149 | 0.885617488 |
| HMDB0000929 | Tryptophan | 656.8227833 | 194.4999686 | 150.9598937 | 304.3681374 | 1026.005823 | 44.07745193 | 334.0942152 | 458.1504709 | 0.729223773 | -0.455566499 | 0.7301853 | 0.136566914 | 0.126128766 |
| HMDB0006236 | Phenylacetaldehyde | 69.00452321 | 74.21998929 | 27.2379965 | 77.58650008 | 118.8399936 | 11.05466135 | 56.82083633 | 69.16038501 | 0.821580683 | -0.283525834 | 0.740387581 | 0.130540874 | 0.173722025 |
| HMDB0000251 | Taurine | 8166.533432 | 3785.614887 | 5392.051413 | 5107.790411 | 8405.839847 | 5577.480717 | 5781.39991 | 6363.703658 | 0.908496093 | -0.138447785 | 0.740867407 | 0.130259511 | 0.542793227 |
| HMDB0001218 | Coumarin | 629.8107317 | 597.9674142 | 643.3169105 | 600.4862836 | 512.4544975 | 886.1233912 | 623.6983521 | 666.3547241 | 0.935985489 | -0.095441932 | 0.742455794 | 0.129329399 | 0.23849644 |
| HMDB0041442 | Hispolone | 66.00808861 | 36.81835161 | 31.10460877 | 58.23290466 | 83.14925781 | 14.2944582 | 44.64368299 | 51.89220689 | 0.860315752 | -0.217061842 | 0.766908838 | 0.115256257 | 0.079378876 |
| HMDB0000114 | Glycerylphosphorylethanolamine | 14.15130685 | 8.42303594 | 2.818944521 | 12.23908326 | 7.524878825 | 1.621384251 | 8.464429104 | 7.128448778 | 1.187415294 | 0.247824601 | 0.780738054 | 0.107494652 | 0.513926528 |
| HMDB0000158 | Tyrosine | 253.7469255 | 37.44740656 | 49.1604754 | 138.0169079 | 204.2873563 | 67.91446773 | 113.4516025 | 136.7395773 | 0.829691043 | -0.269353884 | 0.786766025 | 0.104154403 | 0.705080601 |
| HMDB0062775 | 4-Vinylphenol sulfate | 40.04299361 | 57.80012955 | 26.14430955 | 57.38897637 | 47.29068415 | 3.061437538 | 41.32914424 | 35.91369935 | 1.150790506 | 0.202625224 | 0.790122262 | 0.102305702 | 0.779244809 |
| HMDB0242598 | Phycocyanobilin | 98.66025308 | 88.02293635 | 116.2278529 | 159.900587 | 102.427264 | 1.137369808 | 100.9703472 | 87.82174027 | 1.149719274 | 0.201281642 | 0.794094514 | 0.100127804 | 0.916176791 |
| HMDB0002012 | Ubiquinone-1 | 3.762816097 | 0.959155471 | 141.5770455 | 0.924269241 | 51.16391933 | 56.28531054 | 48.76633902 | 36.12449971 | 1.349951955 | 0.432908063 | 0.811606696 | 0.090654379 | 0.484606573 |
| HMDB0257413 | S-Nitrosocysteinylglycine | 205.1404263 | 136.5596718 | 163.0990353 | 169.1148745 | 196.1838099 | 156.3790196 | 168.2663778 | 173.892568 | 0.967645597 | -0.047449342 | 0.82000643 | 0.086182742 | 0.352764717 |
| HMDB0000190 | Lactic acid | 14169.03214 | 25894.00454 | 10121.85035 | 15235.83275 | 17885.14496 | 13945.12208 | 16728.29568 | 15688.69993 | 1.066263983 | 0.092564661 | 0.841385357 | 0.07500505 | 0.008614187 |
| HMDB0304804 | Leu-Leu-Tyr | 18.2273298 | 49.81566378 | 32.11362162 | 11.39889087 | 72.3573182 | 1.203894158 | 33.3855384 | 28.32003441 | 1.178866449 | 0.237400288 | 0.843293745 | 0.074021121 | 0.898472179 |
| HMDB0248230 | alpha-Methyl-5-HT | 59.00865987 | 33.70567713 | 62.80401582 | 55.49296681 | 119.0307797 | 2.428910783 | 51.83945094 | 58.9842191 | 0.878869836 | -0.186278583 | 0.847873879 | 0.071668744 | 0.605993479 |
| HMDB0260480 | MG(LTE4/0:0/0:0) | 82.90429874 | 24.25944407 | 16.51970616 | 44.39844827 | 97.05854659 | 1.382085674 | 41.22781633 | 47.61302685 | 0.865893623 | -0.207738297 | 0.862986936 | 0.063995778 | 0.488050361 |
| HMDB0000187 | Serine | 933.4157927 | 1839.656791 | 985.8042056 | 1177.591141 | 1191.506004 | 1221.603782 | 1252.95893 | 1196.900309 | 1.0468365 | 0.066036132 | 0.866331689 | 0.0623158 | 0.029817916 |
| HMDB0247233 | 7-Aminoheptanoic acid | 12.92795192 | 24.42230759 | 30.06942796 | 34.7988504 | 24.64189349 | 2.299531133 | 22.47322916 | 20.58009168 | 1.091988778 | 0.12695803 | 0.869882111 | 0.0605396 | 0.643743969 |
| HMDB0000126 | Glycerol 3-phosphate | 1705.03905 | 2481.603144 | 1204.218524 | 2084.32081 | 1851.411618 | 1222.834954 | 1796.953572 | 1719.522461 | 1.04503059 | 0.063545174 | 0.872292257 | 0.059337982 | 0.189900087 |
| HMDB0000996 | 3-Sulfinoalanine | 1754.073974 | 386.482728 | 1054.895036 | 718.2427566 | 940.5811654 | 1357.500373 | 1065.150579 | 1005.441432 | 1.059386003 | 0.083228352 | 0.897926805 | 0.046759064 | 0.191800217 |
| HMDB0010715 | 2-Phenylacetamide | 399.8445726 | 269.1329407 | 233.3590806 | 287.1326824 | 221.4506295 | 369.2215046 | 300.7788646 | 292.6016055 | 1.027946734 | 0.039765509 | 0.90770164 | 0.04205688 | 0.271282441 |
| HMDB0247415 | 8-AHA-cAMP | 16.47967031 | 27.73177622 | 24.66763048 | 25.35015831 | 47.12680176 | 1.31096426 | 22.95969233 | 24.59597478 | 0.933473568 | -0.099318923 | 0.910368257 | 0.040782894 | 0.622771729 |
| HMDB0028713 | Arginylleucine | 5.87589049 | 40.42505686 | 74.64852673 | 33.79389864 | 65.82991447 | 28.77166171 | 40.31649136 | 42.79849161 | 0.942007296 | -0.086189861 | 0.919250034 | 0.036566345 | 0.754306426 |
| HMDB0094648 | Leu-Leu-Leu | 130.0181498 | 21.02705359 | 34.35949197 | 123.6508304 | 48.79254611 | 1.494801327 | 61.80156511 | 57.97939261 | 1.065922948 | 0.092103155 | 0.942072156 | 0.025915832 | 0.67212938 |
| HMDB0003052 | Lactaldehyde | 532.980815 | 605.5355767 | 608.6433352 | 611.3479545 | 649.4577862 | 473.3999686 | 582.3865756 | 578.0685698 | 1.007469712 | 0.010736467 | 0.945092087 | 0.024525873 | 0.087445376 |

## *Supplementary Table 1*
## Negative Mode

| HMDB_ID | Compound_name | DB]a,l]P-#1 | DB]a,l]P-#2 | DB]a,l]P-#3 | DMSO-#1 | DMSO-#2 | DMSO-#3 | AVE (DB[a,l]P) | AVE (DMSO) | FC (DB[a,l]P /DMSO) | Log2(FC) | T-Test | Log10 (P_value) | VIP |
|---|---|---|---|---|---|---|---|---|---|---|---|---|---|---|
| HMDB0001448 | Sulfate | 65117.94627 | 68411.07559 | 76212.12364 | 73689.42354 | 57814.15796 | 79561.34119 | 69913.71517 | 70354.97423 | 0.993728104 | -0.009076927 | 0.954581418 | 0.020187024 | 0.058585432 |
| HMDB0000156 | Malic acid | 1486.561764 | 1863.129801 | 1205.068384 | 1766.95375 | 2291.492567 | 557.7231594 | 1518.253316 | 1538.723159 | 0.986696865 | -0.01932117 | 0.971971982 | 0.012346254 | 0.325499986 |
| HMDB0041708 | Caffeic acid 4-O-sulfate | 175.4110992 | 311.7484288 | 182.2128423 | 197.3034998 | 138.9678497 | 339.9864605 | 223.1241234 | 225.41927 | 0.989818321 | -0.014764348 | 0.976861885 | 0.010166835 | 0.131857888 |
| HMDB0038691 | Kiwiionoside | 1953.009266 | 1319.222899 | 586.3906635 | 1862.696259 | 2014.307255 | 1.272725702 | 1286.20761 | 1292.758747 | 0.994932437 | -0.007329536 | 0.993519488 | 0.00282361 | 0.864158427 |
| HMDB0039947 | Pisumionoside | 108.4493382 | 182.449828 | 138.669961 | 148.2615595 | 278.2637639 | 1.306679743 | 143.1897091 | 142.6106677 | 1.004060295 | 0.005845907 | 0.994757289 | 0.00228287 | 0.820254659 |
| HMDB0006492 | 4-Nitrophenyl sulfate | 1067.57068 | 236.0672016 | 654.031408 | 442.3259924 | 551.5376704 | 958.5436903 | 652.55643 | 650.802451 | 1.002695102 | 0.00388298 | 0.995414188 | 0.001996173 | 0.258787715 |

## *Supplementary Table 1*
## Positive Mode

| HMDB_ID | Compound_name | DB]a,l]P-#1 | DB]a,l]P-#2 | DB]a,l]P-#3 | DMSO-#1 | DMSO-#2 | DMSO-#3 | AVE (DB[a,l]P) | AVE (DMSO) | FC (DB[a,l]P/ DMSO) | Log2 (FC) | T-Test | Log10 (P_value) | VIP |
|---|---|---|---|---|---|---|---|---|---|---|---|---|---|---|
| HMDB0240687 | 8-Amino-7-oxononanoic acid | 49.09970568 | 72.53761092 | 60.85142718 | 18.84738676 | 25.00444362 | 14.75642014 | 60.82958126 | 19.53608351 | 3.113703995 | 1.638631801 | 0.005037935 | 2.297747471 | 2.110805872 |
| HMDB0114747 | LysoPA(20:3/0:0) | 9.548060724 | 8.093424094 | 10.16257476 | 14.62125227 | 20.1391347 | 14.85080292 | 9.268019859 | 16.5370633 | 0.560439281 | -0.83537002 | 0.01880815 | 1.725653923 | 2.028370888 |
| HMDB0260363 | Icosa-5,8,11,14-tetraene | 41.61128106 | 36.97214903 | 70.3429054 | 10.84538828 | 0.709739734 | 13.05945747 | 49.64211183 | 8.204861828 | 6.050328801 | 2.597013547 | 0.020286987 | 1.692782439 | 1.711284342 |
| HMDB0248936 | bemegride | 27.4062299 | 36.7996709 | 29.99199662 | 14.42195046 | 18.76962856 | 4.510838044 | 31.39929914 | 12.56747236 | 2.498457784 | 1.321037842 | 0.020505175 | 1.688136512 | 1.71046213 |
| HMDB0014690 | Propylthiouracil | 7.662339972 | 6.589751682 | 4.283359242 | 2.239295814 | 2.111902039 | 2.876634277 | 6.178483632 | 2.409277377 | 2.56445509 | 1.358652306 | 0.021224649 | 1.673159474 | 2.023107426 |
| HMDB0249590 | Cannabinol | 257.4578294 | 279.078916 | 416.9174227 | 111.6749213 | 151.7956284 | 41.8035875 | 317.818056 | 101.7580457 | 3.123272011 | 1.643058221 | 0.022001077 | 1.657556051 | 1.859341742 |
| HMDB0029000 | Phenylalanyllysine | 9.502427164 | 16.87904426 | 11.73934651 | 5.866154424 | 4.200591219 | 2.623410521 | 12.70693931 | 4.230052055 | 3.003967599 | 1.586869252 | 0.023421789 | 1.630379928 | 1.979571914 |
| HMDB0059793 | Gluten exorphin A5 | 32.46390065 | 56.58431794 | 72.08935476 | 17.04468624 | 8.984175827 | 8.756943634 | 53.71252445 | 11.59526857 | 4.632279463 | 2.211722293 | 0.023686368 | 1.625501521 | 2.05687913 |
| HMDB0032948 | 5-Phenyl-1,3-pentadiyne | 66.53140922 | 111.6609828 | 106.1952825 | 45.93428496 | 40.00489078 | 54.00875122 | 94.79589152 | 46.64930898 | 2.032096372 | 1.022968823 | 0.031203546 | 1.505796045 | 1.959819758 |
| HMDB0246857 | Dehydronorketamine | 358.1431666 | 650.8584215 | 705.3078907 | 193.6028539 | 237.0555095 | 287.7539342 | 571.4364929 | 239.4707659 | 2.386247402 | 1.254743627 | 0.040503006 | 1.392512741 | 1.931621642 |
| HMDB0002013 | Butyrylcarnitine | 57.59996971 | 68.59935128 | 54.45865604 | 49.339257 | 25.94457696 | 24.88785951 | 60.21932568 | 33.39056449 | 1.803483307 | 0.85078607 | 0.04148744 | 1.382083365 | 1.792842346 |
| HMDB0014817 | Thioridazine | 20.0710426 | 30.75355991 | 41.06715898 | 14.25514081 | 0.45117513 | 13.52684972 | 30.63058716 | 9.411055221 | 3.254745238 | 1.702544622 | 0.048111134 | 1.317754406 | 1.401922214 |
| HMDB0258754 | Tert-Butylbicyclophosphorothionate | 299.4965776 | 299.8859436 | 296.0355364 | 217.5839197 | 103.9741514 | 138.1077103 | 298.4726859 | 153.2219272 | 1.94797632 | 0.96197614 | 0.049545995 | 1.304991449 | 1.901171866 |
| HMDB0028951 | Lysylglycine | 20.77893266 | 15.52921915 | 11.31259654 | 5.521096872 | 10.12582517 | 2.355460452 | 15.87358278 | 6.000794165 | 2.645247003 | 1.403402442 | 0.049673499 | 1.303875247 | 1.707544334 |
| HMDB0248473 | Antiarrhythmic peptide | 116.6058658 | 170.872721 | 141.7465801 | 103.587051 | 89.89267719 | 50.52543571 | 143.0750557 | 81.33505463 | 1.759082308 | 0.814822989 | 0.050617677 | 1.295697786 | 1.722162743 |
| HMDB0240264 | 6-Hydroxypseudooxynicotine | 40.74858141 | 67.92306421 | 86.40181732 | 39.14692781 | 7.648586431 | 13.60314012 | 65.02448765 | 20.13288479 | 3.22976505 | 1.69142922 | 0.052094966 | 1.283204243 | 1.76234889 |
| HMDB0000038 | Dihydrobiopterin | 12.15449129 | 27.35853549 | 23.08017664 | 12.37602327 | 6.080469344 | 4.706914409 | 20.86440114 | 7.721135675 | 2.702245113 | 1.434158543 | 0.061692518 | 1.209767506 | 1.792712441 |
| HMDB0032033 | 2,4,12-Octadecatrienoic acid isobutylamide | 508.91864 | 377.4342982 | 381.5715742 | 289.3484345 | 161.6294351 | 319.0226923 | 422.6415041 | 256.666854 | 1.646654009 | 0.719537452 | 0.062443025 | 1.204516066 | 1.654638571 |
| HMDB0028994 | Phenylalanylglutamic acid | 7.432856468 | 6.440453586 | 6.399923314 | 3.397248778 | 1.500317593 | 5.756639124 | 6.757744456 | 3.551401832 | 1.90283859 | 0.928153189 | 0.065943013 | 1.180831214 | 1.553999851 |
| HMDB0253034 | Val-pro-pro | 53.49264192 | 90.0421171 | 121.7438087 | 55.52521233 | 13.78931229 | 17.21125661 | 88.42618925 | 28.84192708 | 3.065890466 | 1.616306155 | 0.066730072 | 1.175678408 | 1.760782628 |
| HMDB0013132 | Hydroxyvalerylcarnitine | 42.03787968 | 87.80707015 | 57.83898775 | 38.9817022 | 26.77864118 | 14.98044355 | 62.56131253 | 26.91359564 | 2.324524502 | 1.216935633 | 0.077659828 | 1.109803575 | 1.735354614 |
| HMDB0000101 | Deoxyadenosine | 588.6073026 | 1747.819844 | 1082.293564 | 390.5594054 | 224.7705573 | 399.4685478 | 1139.57357 | 338.2661702 | 3.368866503 | 1.752263261 | 0.078307034 | 1.106199227 | 1.87416291 |
| HMDB0000366 | 2-Deoxyribonic acid | 43.49824372 | 23.84507165 | 19.94534782 | 3.399187175 | 9.99824452 | 16.30948451 | 29.09622107 | 9.902305401 | 2.938327984 | 1.554995442 | 0.078974181 | 1.102514871 | 1.667236581 |
| HMDB0244865 | 1-Benzazepine | 370.5353855 | 564.1849946 | 596.5254868 | 421.7533717 | 150.5597405 | 96.64389479 | 510.415289 | 222.985669 | 2.2890049 | 1.194720551 | 0.079446777 | 1.099923718 | 1.644980301 |
| HMDB0015705 | Ivacaftor | 33738.03454 | 54689.0557 | 57737.06169 | 38820.01086 | 14797.42783 | 5502.074362 | 48721.38398 | 19706.50435 | 2.472350403 | 1.305883229 | 0.080490196 | 1.094257013 | 1.577549483 |
| HMDB0000828 | Ureidosuccinic acid | 436.2622583 | 733.1788227 | 825.2874409 | 273.1775163 | 282.8328613 | 274.6370535 | 664.9095073 | 276.882477 | 2.401414183 | 1.263884253 | 0.080498589 | 1.094211734 | 2.023137731 |
| HMDB0034244 | Isoquinoline | 4981.966404 | 8165.015514 | 8245.843736 | 5785.139034 | 2157.064204 | 981.6692167 | 7130.941885 | 2974.624152 | 2.397258114 | 1.261385253 | 0.082275564 | 1.084729134 | 1.598469673 |
| HMDB0001505 | Benzene | 4827.407116 | 7722.60886 | 8130.837595 | 5575.530081 | 2191.337446 | 965.5103808 | 6893.617857 | 2910.792636 | 2.36829576 | 1.24384926 | 0.082342837 | 1.084374175 | 1.593349044 |
| HMDB0000030 | Biotin | 129.6060753 | 203.4224099 | 243.053113 | 149.0758008 | 56.74475293 | 22.37981552 | 192.0271994 | 76.06678975 | 2.524455154 | 1.335972048 | 0.082693085 | 1.082530805 | 1.594354912 |
| HMDB0015535 | Cilastatin | 879.441895 | 1430.563252 | 1528.996258 | 1022.872524 | 390.1303532 | 148.3770465 | 1279.667135 | 520.4599746 | 2.458723432 | 1.297909464 | 0.08281193 | 1.081907094 | 1.576709145 |
| HMDB0029374 | Trichostachine | 202.0317936 | 364.8949465 | 346.5416099 | 239.6210265 | 96.32078008 | 38.75455546 | 304.48945 | 124.8987873 | 2.437889562 | 1.285632773 | 0.085036496 | 1.070394643 | 1.580850845 |
| HMDB0013113 | 1-Undecanol | 52.18953899 | 35.9144372 | 38.22559603 | 24.86084646 | 14.79217825 | 34.74198855 | 42.10985741 | 24.79833776 | 1.698091938 | 0.763914571 | 0.087303043 | 1.058970616 | 1.594948438 |
| HMDB0034276 | L,L-Cyclo(leucylprolyl) | 2.30133078 | 1.966266568 | 1.363717568 | 1.122732804 | 0.704356055 | 1.44804601 | 1.877104972 | 1.091711623 | 1.719414663 | 0.781917514 | 0.087404944 | 1.058463999 | 1.602644248 |
| HMDB0000738 | Indole | 305.8458782 | 491.9190767 | 502.03645 | 354.028797 | 139.2455708 | 194.639667 | 433.2671349 | 229.3046783 | 1.889482318 | 0.917991017 | 0.087577272 | 1.057608586 | 1.640643192 |
| HMDB0245680 | 2-Deoxy-2,3-dehydro-n-acetyl-neuraminic acid | 205.3728329 | 638.4290015 | 405.1852511 | 120.4659722 | 130.2420708 | 152.0499051 | 416.3290285 | 134.2526494 | 3.101086127 | 1.632773595 | 0.087849283 | 1.056261776 | 1.863914641 |
| HMDB0000933 | Traumatic acid | 84.81558985 | 90.20454782 | 51.22197639 | 61.46092372 | 24.14017287 | 13.545987 | 75.41403802 | 33.04902787 | 2.281883701 | 1.190225265 | 0.089297614 | 1.049160145 | 1.596543981 |
| HMDB0000951 | Taurochenodesoxycholic acid | 1250.051932 | 3967.729515 | 4025.023217 | 1209.474908 | 65.25561234 | 1338.748279 | 3080.934888 | 871.1595997 | 3.536590642 | 1.822359238 | 0.09188236 | 1.036767857 | 1.410886518 |
| HMDB0246904 | Ethyl butylacetylaminopropionate | 775.6388532 | 1278.291936 | 1313.676622 | 937.5095032 | 347.5437914 | 132.9630007 | 1122.535804 | 472.6720985 | 2.374872152 | 1.24784985 | 0.093677477 | 1.028364817 | 1.551273732 |
| HMDB0033944 | 2-Phenylethanol | 132.5387161 | 442.7653242 | 413.5596269 | 133.7973584 | 14.20401748 | 143.4558969 | 329.6212224 | 97.15242427 | 3.3928255 | 1.762487231 | 0.096142881 | 1.017082867 | 1.465627696 |
| HMDB0030776 | Maltol | 22.07315405 | 17.08095614 | 20.7320467 | 13.06975177 | 15.33993648 | 0.953994555 | 19.9620523 | 9.787894269 | 2.039463418 | 1.028189629 | 0.09676991 | 1.014259664 | 1.263338338 |
| HMDB0015095 | Pindolol | 202.0937073 | 319.9397987 | 362.144573 | 249.3875899 | 82.83283978 | 38.82250369 | 294.7260263 | 123.6809778 | 2.38295356 | 1.252750836 | 0.099407498 | 1.002580857 | 1.573761318 |
| HMDB0000206 | N6-Acetyllysine | 89.13241513 | 134.6888675 | 205.6497513 | 104.2119581 | 27.95831823 | 26.37508968 | 143.1570113 | 52.84845534 | 2.708821107 | 1.43766512 | 0.100970273 | 0.995806471 | 1.680145677 |
| HMDB0001080 | 4-Aminobutyraldehyde | 1004.138371 | 1185.383011 | 1116.777671 | 1060.604472 | 479.964554 | 399.8526042 | 1102.099685 | 646.80721 | 1.703907544 | 0.768847055 | 0.101371138 | 0.99408568 | 1.599626563 |
| HMDB0029109 | Tyrosyl-Leucine | 11.52424083 | 16.58011489 | 7.75154168 | 14.95175358 | 56.35298374 | 69.1390709 | 11.9519658 | 46.81460274 | 0.255304223 | -1.96971069 | 0.102927726 | 0.987467624 | 1.693195359 |
| HMDB0000875 | Trigonelline | 271.9538593 | 318.8394309 | 448.4085697 | 251.1920806 | 151.0101578 | 247.5283922 | 346.40062 | 216.5768769 | 1.599434921 | 0.677562292 | 0.104894435 | 0.979247551 | 1.630001107 |
| HMDB0255970 | Oxazine | 199.2265979 | 339.6753938 | 322.4127675 | 171.6655697 | 60.13257282 | 222.5767115 | 287.1049197 | 151.4582847 | 1.895603931 | 0.922657558 | 0.106128958 | 0.9741661 | 1.451004224 |
| HMDB0041792 | 1,3-Butadiene | 69.20596647 | 90.49029173 | 102.3362379 | 81.69910616 | 26.68709431 | 24.31387787 | 87.34416538 | 44.23335945 | 1.974622015 | 0.981576517 | 0.110580361 | 0.956321998 | 1.597534199 |
| HMDB0012234 | Histidinal | 20.39174125 | 135.4527928 | 86.16377146 | 25.27208212 | 6.968111821 | 10.14169631 | 80.66943516 | 14.12729675 | 5.710181968 | 2.513536721 | 0.120378172 | 0.919452256 | 1.706534415 |
| HMDB0013299 | Pentadecanol | 675.4235934 | 503.3009328 | 569.0659728 | 517.9767619 | 301.0642569 | 454.6550236 | 582.596833 | 424.5653475 | 1.372219463 | 0.456511234 | 0.124925077 | 0.903350375 | 1.48576711 |
| HMDB0000824 | Propionylcarnitine | 110.7004358 | 168.270181 | 86.77941206 | 95.88241884 | 62.83758467 | 22.33731483 | 121.9166763 | 60.35243945 | 2.020078681 | 1.014411486 | 0.128499162 | 0.891099705 | 1.460193814 |

*Supplementary Table 1*
**Positive Mode**

| HMDB_ID | Compound_name | DB]a,l]P-#1 | DB]a,l]P-#2 | DB]a,l]P-#3 | DMSO-#1 | DMSO-#2 | DMSO-#3 | AVE (DB[a,l]P) | AVE (DMSO) | FC (DB[a,l]P/ DMSO) | Log2 (FC) | T-Test | Log10 (P_value) | VIP |
|---|---|---|---|---|---|---|---|---|---|---|---|---|---|---|
| HMDB0253033 | Val-Gly-Val-Ala-Pro-Gly | 245.7325182 | 209.6586123 | 84.31662488 | 128.5435854 | 53.76286602 | 28.51446977 | 179.9025852 | 70.2736404 | 2.560029395 | 1.356160376 | 0.128751368 | 0.890248148 | 1.507473111 |
| HMDB0302463 | Cyclopentanol | 19009.08522 | 18345.52818 | 16287.51202 | 17276.46413 | 12234.06062 | 7732.146267 | 17880.70847 | 12414.22367 | 1.440340447 | 0.526409855 | 0.130115633 | 0.885670523 | 1.462851516 |
| HMDB0011179 | Prolylphenylalanine | 14.89512157 | 21.51026154 | 14.48028347 | 15.94325904 | 4.517560252 | 6.782014778 | 16.96188886 | 9.08094469 | 1.867855101 | 0.901382542 | 0.131742995 | 0.880272468 | 1.530462086 |
| HMDB0094708 | Tetraethylene glycol | 81.99185434 | 38.60883603 | 255.9137827 | 131.9948428 | 740.9122788 | 602.0692015 | 125.5048243 | 491.6587743 | 0.255268147 | -1.96991457 | 0.134888711 | 0.870024394 | 1.491299336 |
| HMDB0029819 | 2-Phenylethyl betaglucopyranoside | 22.77638062 | 95.44251991 | 71.53552323 | 236.5330232 | 189.7516172 | 535.5912537 | 63.25147459 | 320.625298 | 0.197275371 | -2.34171724 | 0.135908476 | 0.866753458 | 1.863312228 |
| HMDB0246748 | 5-Aminoimidazole-4-carboxyamide | 46.2327006 | 144.5037557 | 109.2735047 | 72.4213894 | 9.355423208 | 27.33232192 | 100.0033203 | 36.36971151 | 2.749631937 | 1.459238514 | 0.137362723 | 0.862131108 | 1.486942018 |
| HMDB0059809 | 4-Hydroxy-benzenepropanedioate | 92.65146918 | 243.6255068 | 130.0530912 | 115.2959766 | 18.84031089 | 46.10839078 | 155.4433557 | 60.08155943 | 2.587205745 | 1.371394788 | 0.150469924 | 0.822550299 | 1.525388277 |
| HMDB0000062 | Carnitine | 4717.191409 | 6705.20444 | 7020.859983 | 6078.060846 | 3047.497076 | 2225.527165 | 6147.751944 | 3783.695029 | 1.624801126 | 0.700263144 | 0.160818811 | 0.793663154 | 1.471590269 |
| HMDB0028958 | Lysylphenylalanine | 40.13294771 | 206.7425142 | 158.274317 | 40.89540196 | 24.44981545 | 76.40800937 | 135.0499263 | 47.25107559 | 2.858134436 | 1.515073777 | 0.165322966 | 0.781666812 | 1.376216243 |
| HMDB0039228 | (3-Aminopropoxy)guanidine | 259.5366335 | 2669.384196 | 1951.379759 | 523.2838211 | 66.09743951 | 568.6161604 | 1626.766863 | 385.9991403 | 4.21443131 | 2.075337969 | 0.165381637 | 0.781512713 | 1.274803424 |
| HMDB0014832 | Daunorubicin | 61.47577319 | 166.7288929 | 136.7325876 | 103.1552502 | 12.64627785 | 39.72501026 | 121.6457512 | 51.84217943 | 2.34646291 | 1.230487656 | 0.165658717 | 0.780785708 | 1.401931789 |
| HMDB0032931 | 2-Benzoxazolol | 54.05307371 | 126.058152 | 103.2702369 | 67.44204379 | 41.71012588 | 59.68432904 | 94.46048754 | 56.2788329 | 1.678437215 | 0.747118571 | 0.166005803 | 0.779876731 | 1.410583119 |
| HMDB0245091 | 2-Cyclooctyl-2-hydroxyethylamine | 848.3097974 | 958.3488993 | 908.7858074 | 728.6423276 | 361.7858069 | 861.3710188 | 905.148168 | 650.5997177 | 1.391252015 | 0.476383777 | 0.170963452 | 0.767096722 | 1.30755971 |
| HMDB0037543 | 2-(5-Tetradecenyl)cyclobutanone | 11895.37184 | 6620.246098 | 8147.557223 | 5737.88328 | 3053.300552 | 7630.489916 | 8887.725054 | 5473.891249 | 1.623657586 | 0.699247414 | 0.171850213 | 0.764849924 | 1.392518342 |
| HMDB0000201 | Acetylcarnitine | 344.1551424 | 433.8387727 | 158.9820954 | 195.2937896 | 186.7743321 | 145.3577937 | 312.3253368 | 175.8086384 | 1.776507341 | 0.82904365 | 0.172832919 | 0.762373536 | 1.349042882 |
| HMDB0304540 | betaribosylnicotinate | 130.0328471 | 322.5435721 | 261.4562229 | 220.7428477 | 14.38075437 | 62.95494421 | 238.0108807 | 99.35951542 | 2.395451303 | 1.260297485 | 0.175354762 | 0.756082437 | 1.388926864 |
| HMDB0001458 | Biotin amide | 33.43273838 | 30.72035933 | 45.22486217 | 32.89550745 | 26.76237121 | 11.38835812 | 36.45931996 | 23.68207892 | 1.539532069 | 0.622491919 | 0.176462318 | 0.75334802 | 1.338806782 |
| HMDB0003252 | Thromboxane B2 | 183.2243423 | 365.3445507 | 300.575179 | 274.8480454 | 63.16757449 | 96.57660554 | 283.048024 | 144.8640751 | 1.953886937 | 0.966346987 | 0.177741064 | 0.750212225 | 1.451579116 |
| HMDB0250013 | Ser-Leu-Ile-Gly-Lys-Val | 19.33253071 | 174.9129279 | 173.5342759 | 52.1069381 | 1.337819709 | 51.13770051 | 122.5932449 | 34.86081944 | 3.516648398 | 1.814201099 | 0.181369056 | 0.741436807 | 1.145927931 |
| HMDB0249012 | Benzodiazepine | 18.74406967 | 33.04114721 | 34.63334726 | 20.45924322 | 17.80904464 | 18.05801597 | 28.80618805 | 18.77543461 | 1.534248801 | 0.617532456 | 0.182513015 | 0.73870616 | 1.567389596 |
| HMDB0028726 | Asparaginylasparagine | 112.606406 | 156.3866168 | 140.1894973 | 140.0613705 | 73.66244073 | 28.06414686 | 136.3941734 | 80.59598603 | 1.692319681 | 0.759002121 | 0.185484504 | 0.731692366 | 1.340641393 |
| HMDB0028989 | Phenylalanylarginine | 1140.198877 | 1285.639762 | 1639.403318 | 1440.464397 | 496.4055526 | 380.9399145 | 1355.080652 | 772.6032881 | 1.753915202 | 0.810578998 | 0.187548428 | 0.726886572 | 1.463284931 |
| HMDB0252011 | Ethenoadenosine | 768.027202 | 1994.467945 | 1538.759348 | 1337.972312 | 141.870143 | 450.3984546 | 1433.751498 | 643.4136364 | 2.228351122 | 1.155976576 | 0.193752116 | 0.712753545 | 1.370708543 |
| HMDB0001850 | Verapamil | 6.950851884 | 10.23764369 | 8.18860213 | 8.374686777 | 5.540647091 | 3.762978153 | 8.459032567 | 5.892770673 | 1.435493257 | 0.521546554 | 0.194807127 | 0.710395159 | 1.375098987 |
| HMDB0059907 | Tetradecane | 55.38396627 | 59.53535522 | 68.96358701 | 39.92814235 | 26.06173936 | 63.61105864 | 61.29430284 | 43.20031345 | 1.418839308 | 0.504711205 | 0.19612944 | 0.707457213 | 1.358059125 |
| HMDB0000583 | Docosanamide | 0.835113906 | 2.051507688 | 5.347063978 | 22.36349145 | 2.53387868 | 56.5791229 | 2.744561857 | 27.15883101 | 0.101055964 | -3.30677362 | 0.198145209 | 0.703016423 | 1.491571512 |
| HMDB0036232 | Maleic acid homopolymer | 84.60149002 | 161.284558 | 139.1284075 | 133.7290835 | 19.21902419 | 37.50348355 | 128.3381518 | 63.48386374 | 2.021586972 | 1.015488272 | 0.199120452 | 0.700884132 | 1.418858162 |
| HMDB0041370 | Na-p-Hydroxycoumaroyltryptophan | 26.80819431 | 2.963062453 | 2.095986537 | 129.2116032 | 133.1015259 | 4.491232873 | 10.62241443 | 88.93478734 | 0.119440488 | -3.06563613 | 0.201491642 | 0.695742964 | 1.327274896 |
| HMDB0029225 | Coutaric acid | 33.84326946 | 58.7879098 | 55.43397941 | 44.10761976 | 35.73487042 | 18.22514984 | 49.35505289 | 32.68921334 | 1.509826877 | 0.594383134 | 0.2016505 | 0.695400697 | 1.293461828 |
| HMDB0000252 | Sphingosine | 49.98181835 | 8.006506195 | 19.47724779 | 8.537791905 | 3.601606418 | 8.254150803 | 25.82185744 | 6.797849709 | 3.798533146 | 1.92544241 | 0.206393009 | 0.685305016 | 1.524736252 |
| HMDB0255245 | N-VINYL-2-PYRROLIDONE | 186.5717857 | 67.13754092 | 54.29078264 | 249.0376969 | 240.5502461 | 103.6087717 | 102.6667031 | 197.7322382 | 0.519220862 | -0.94557974 | 0.206985636 | 0.684059792 | 1.369237475 |
| HMDB0000517 | Arginine | 1389.623322 | 1275.568021 | 1067.725485 | 1277.584483 | 849.9755245 | 449.1963345 | 1244.30561 | 858.9187806 | 1.448688325 | 0.534747243 | 0.208208047 | 0.681502489 | 1.297521635 |
| HMDB0028990 | Phenylalanylasparagine | 901.669922 | 2234.296493 | 2024.704373 | 1540.499158 | 798.0395692 | 522.2389348 | 1720.223596 | 953.5925538 | 1.803939837 | 0.851151224 | 0.209697048 | 0.678407683 | 1.317121596 |
| HMDB0253472 | Indoline | 170.6488539 | 136.5759859 | 206.4850048 | 160.6917421 | 135.0333584 | 85.87608563 | 171.2366149 | 127.2003954 | 1.346195618 | 0.428888066 | 0.213780136 | 0.670032652 | 1.30935656 |
| HMDB0040215 | 3-Hexenyl acetate | 368.1451517 | 534.2253069 | 385.1573937 | 430.6051093 | 278.8936022 | 180.2026336 | 429.1759508 | 296.567115 | 1.447146123 | 0.533210603 | 0.214315081 | 0.668947268 | 1.338306182 |
| HMDB0245773 | 3-(2-Hydroxyethyl)histidine | 19.05928248 | 41.18659809 | 32.03971206 | 17.18466132 | 11.47629936 | 28.30492045 | 30.76186421 | 18.98862704 | 1.620015188 | 0.696007339 | 0.2197942 | 0.657983773 | 1.303331968 |
| HMDB0001587 | Phenylglyoxylic acid | 11.91885447 | 94.73248161 | 74.81757424 | 29.92992721 | 1.898075369 | 33.55618388 | 60.48963677 | 21.79472882 | 2.775425071 | 1.472708745 | 0.223503002 | 0.650716638 | 1.078093515 |
| HMDB0028886 | Histidylhydroxyproline | 1043.474509 | 1844.846924 | 1707.814657 | 1585.336069 | 782.5710729 | 274.0214687 | 1532.045363 | 880.6428702 | 1.739689737 | 0.798830033 | 0.225423335 | 0.647001129 | 1.286308878 |
| HMDB0000413 | 3-Hydroxydodecanedioic acid | 7.156449118 | 10.71486255 | 15.04119851 | 81.90756259 | 18.41299746 | 32.26479405 | 10.97083673 | 44.19511803 | 0.248236394 | -2.01021345 | 0.225675661 | 0.646515277 | 1.740929547 |
| HMDB0242048 | N-Myristoyl Aspartic acid | 29.50486099 | 34.77540089 | 73.25309444 | 13.96545932 | 20.6952459 | 36.93710004 | 45.8444521 | 23.86593509 | 1.920915813 | 0.941794292 | 0.226255588 | 0.645400685 | 1.420159805 |
| HMDB0243900 | 1-Hydroxyisoquinoline | 73.75597355 | 84.53008183 | 72.77056516 | 78.56729203 | 30.77825846 | 60.03287124 | 77.01887351 | 56.45947391 | 1.364144371 | 0.447996337 | 0.226856079 | 0.644249578 | 1.23307206 |
| HMDB0001123 | 2-Aminobenzoic acid | 403.3818194 | 1050.178525 | 769.8476477 | 735.5243976 | 65.32381528 | 271.6835442 | 741.1359973 | 357.5105857 | 2.073046301 | 1.051752339 | 0.232170971 | 0.634192081 | 1.286076593 |
| HMDB0006294 | 16-Hydroxyhexadecanoic acid | 1298.806143 | 1123.809965 | 1161.239004 | 982.8715142 | 443.8917348 | 1191.569783 | 1194.618371 | 872.7776774 | 1.368754497 | 0.452863705 | 0.23262397 | 0.633345538 | 1.194658927 |
| HMDB0013316 | Tridecanol | 214.337464 | 80.68211747 | 75.50357895 | 58.72920871 | 35.32770428 | 79.11583558 | 123.5077201 | 57.72424952 | 2.139615866 | 1.097351807 | 0.235578865 | 0.627863676 | 1.407851409 |
| HMDB0254648 | Methylglutarylcarnitine | 31.00818942 | 62.39588204 | 17.45340928 | 30.29789309 | 12.9603502 | 5.532232803 | 36.95249358 | 16.26349203 | 2.272113117 | 1.184034661 | 0.245068714 | 0.610712128 | 1.318641034 |
| HMDB0010350 | 2-Phenylethanol glucuronide | 13.91110953 | 24.60396169 | 28.4162911 | 19.47857013 | 6.484473771 | 16.95371673 | 22.31045411 | 14.30558688 | 1.559562309 | 0.641141194 | 0.245592866 | 0.609784252 | 1.175073767 |
| HMDB0249273 | Bisnortilidin | 21.25261548 | 19.62796586 | 18.8682222 | 8.744928773 | 99.78632542 | 141.1220006 | 19.91626785 | 83.21775158 | 0.239327156 | -2.06294399 | 0.24686197 | 0.60754581 | 1.03658987 |
| HMDB0039539 | 13-Tetradecene-1,3-diyne-6,7-diol | 74.29031113 | 119.0919466 | 121.9889171 | 86.50752606 | 41.8181019 | 95.79559769 | 105.123725 | 74.70707522 | 1.407145503 | 0.492771515 | 0.251587388 | 0.599311134 | 1.171461475 |

## *Supplementary Table 1*
## Positive Mode

| HMDB_ID | Compound_name | DB]a,l]P-#1 | DB]a,l]P-#2 | DB]a,l]P-#3 | DMSO-#1 | DMSO-#2 | DMSO-#3 | AVE (DB[a,l]P) | AVE (DMSO) | FC (DB[a,l]P/ DMSO) | Log2 (FC) | T-Test | Log10 (P_value) | VIP |
|---|---|---|---|---|---|---|---|---|---|---|---|---|---|---|
| HMDB0013034 | Palmitoylglycine | 40.35216966 | 10.79079254 | 13.55573991 | 6.795592542 | 4.210976901 | 9.021879187 | 21.56623404 | 6.676149543 | 3.230340168 | 1.691686094 | 0.253484475 | 0.596048634 | 1.644789098 |
| HMDB0251974 | Estrane | 1972.532977 | 2087.743523 | 2481.436009 | 1438.387778 | 906.5068421 | 2378.766082 | 2180.570836 | 1574.553567 | 1.384881964 | 0.469763018 | 0.255612421 | 0.592418046 | 1.262645965 |
| HMDB0028791 | Glutaminylarginine | 734.9294211 | 1228.828741 | 1041.268056 | 1080.847157 | 573.1313289 | 164.2460878 | 1001.675406 | 606.0748577 | 1.652725556 | 0.724847177 | 0.259940639 | 0.585125817 | 1.215925799 |
| HMDB0062602 | 1-docosene | 11.42403889 | 118.0960632 | 733.3338276 | 561.4809421 | 402.1342397 | 1520.889244 | 287.6179766 | 828.1681418 | 0.347294181 | -1.52576986 | 0.263217054 | 0.579685977 | 1.328906944 |
| HMDB0303832 | 1,3,5-octatriene | 215.2261554 | 169.0257475 | 214.17701 | 167.3858234 | 93.40080416 | 201.0571837 | 199.4763043 | 153.9479371 | 1.295738729 | 0.373774844 | 0.266129399 | 0.574907147 | 1.165088135 |
| HMDB0011175 | Leucylproline | 19.9800675 | 41.56813816 | 15.2966385 | 19.10429339 | 15.38651025 | 9.411270752 | 25.61494806 | 14.6340248 | 1.750369321 | 0.807659358 | 0.269263577 | 0.569822389 | 1.257769778 |
| HMDB0000600 | Galactosylhydroxylysine | 147.4034463 | 268.5102843 | 583.2147135 | 276.2547779 | 59.88096205 | 102.8038976 | 333.0428147 | 146.3132125 | 2.276231989 | 1.186647602 | 0.26937244 | 0.56964684 | 1.324246273 |
| HMDB0028709 | Arginylglycine | 49.56509983 | 66.25082272 | 49.15418689 | 62.80423105 | 28.43473347 | 14.67174272 | 54.99003648 | 35.30356908 | 1.557633914 | 0.639356201 | 0.269760607 | 0.569021469 | 1.280217932 |
| HMDB0000870 | Histamine | 34.37328087 | 67.72102536 | 39.38648815 | 48.42921371 | 21.3520549 | 17.23568909 | 47.1602648 | 29.00565257 | 1.625899114 | 0.701237742 | 0.272100043 | 0.56527139 | 1.303961576 |
| HMDB0000269 | Sphinganine | 5180.826216 | 5156.040848 | 5721.119361 | 4697.063255 | 2548.172923 | 5436.763516 | 5352.662142 | 4227.333231 | 1.266203029 | 0.340508752 | 0.272762492 | 0.564215351 | 1.149366228 |
| HMDB0000687 | Leucine | 148.7512229 | 78.45301189 | 57.74612597 | 206.9769731 | 244.6643792 | 64.88205818 | 94.9834536 | 172.1744702 | 0.551669789 | -0.85812312 | 0.276258344 | 0.558684596 | 1.040381802 |
| HMDB0040833 | 3-[4-Hydroxy-3-(3-methyl-2-butenyl)phenyl]-2-propenal | 0.774621819 | 0.9038341 | 2.977353516 | 2.022904478 | 44.80450903 | 14.34502359 | 1.551936478 | 20.39081236 | 0.076109595 | -3.71577784 | 0.276418363 | 0.55843311 | 1.613513873 |
| HMDB0000895 | Acetylcholine | 76.51768074 | 119.1655905 | 90.65982786 | 104.6262304 | 57.58822277 | 27.93049764 | 95.44769972 | 63.38165027 | 1.505920078 | 0.590645205 | 0.278754788 | 0.554777665 | 1.228527348 |
| HMDB0032797 | Jasmonic acid | 42.55615354 | 38.75688228 | 45.70523528 | 35.79638775 | 14.42940288 | 43.44585066 | 42.3394237 | 31.22388043 | 1.355994935 | 0.439351789 | 0.280346434 | 0.552304964 | 1.122825511 |
| HMDB0303903 | 2-dodecen-1-ol | 43.12326802 | 49.90718811 | 58.93705747 | 40.63937088 | 23.34951915 | 52.27484438 | 50.65583787 | 38.75457814 | 1.307093002 | 0.386361795 | 0.281564964 | 0.550421387 | 1.166112522 |
| HMDB0032460 | 4-Pentenyl acetate | 5.416289193 | 13.39514907 | 16.13207273 | 20.2266604 | 9.664332132 | 48.52245337 | 11.647837 | 26.1378153 | 0.445631621 | -1.16607649 | 0.295048775 | 0.530106184 | 1.143247938 |
| HMDB0253028 | Pro-leu | 76.72513862 | 14.11419155 | 21.38495813 | 51.05446878 | 217.5185879 | 54.37732692 | 37.4080961 | 107.6501279 | 0.347496996 | -1.52492759 | 0.295326561 | 0.529697493 | 1.373189877 |
| HMDB0032397 | Methyl-delta-ionone | 57.98932858 | 34.32788254 | 43.95219814 | 41.77785677 | 16.76593298 | 39.65572878 | 45.42313642 | 32.73317284 | 1.387678996 | 0.472673876 | 0.295378927 | 0.529620492 | 1.099725259 |
| HMDB0034368 | Hydroxyminaline | 184.2080736 | 152.803588 | 121.4502981 | 177.3410047 | 73.75764194 | 57.30464782 | 152.8206532 | 102.8010981 | 1.48656635 | 0.571983856 | 0.296633415 | 0.527779928 | 1.265152455 |
| HMDB0013243 | Leucylphenylalanine | 88.71575058 | 305.7150478 | 710.0481532 | 191.367304 | 88.58105254 | 161.661041 | 368.1596506 | 147.2031325 | 2.501031359 | 1.322523147 | 0.297423795 | 0.526624289 | 1.022289841 |
| HMDB0039884 | 2-Cyclotetradecen-1-one | 50.64276687 | 17.19789139 | 14.61472515 | 12.93144092 | 7.354666184 | 19.01576097 | 27.4851278 | 13.10062269 | 2.098001633 | 1.069015801 | 0.299630941 | 0.523413341 | 1.226199508 |
| HMDB0029121 | Valylarginine | 49.66542147 | 70.4754861 | 60.2004122 | 72.99281326 | 18.531616 | 23.67371397 | 60.11377326 | 38.39938108 | 1.565488077 | 0.646612521 | 0.302660297 | 0.519044547 | 1.305492594 |
| HMDB0302964 | 24-Hydroxytetracosanoic acid | 155.7691232 | 314.8633698 | 578.9146116 | 285.8739418 | 66.74422905 | 204.5309998 | 349.8490348 | 185.7163902 | 1.883781148 | 0.913631367 | 0.303051187 | 0.518484011 | 1.115841961 |
| HMDB0029033 | Serylarginine | 36.83595986 | 86.75042651 | 79.01031147 | 63.30261953 | 43.8063381 | 32.53885495 | 67.53223261 | 46.54927086 | 1.45076886 | 0.536817684 | 0.306742051 | 0.513226683 | 1.036881661 |
| HMDB0038594 | Calystegine B2 | 1.491232447 | 1.627106873 | 1.209072906 | 1.110951872 | 1.549787114 | 0.766079041 | 1.442470742 | 1.142272675 | 1.26280771 | 0.336634975 | 0.309347436 | 0.50955348 | 1.139082721 |
| HMDB0004085 | Tuberculostearic acid | 60.42225003 | 74.14642512 | 77.92436054 | 39.99222645 | 25.33214282 | 83.56113099 | 70.8310119 | 49.62850009 | 1.427224514 | 0.5132123 | 0.310540601 | 0.507881611 | 1.222403382 |
| HMDB0092959 | DG(10:0/8:0/0:0) | 3.011428997 | 5.838410744 | 2.457671824 | 4.15216066 | 4.961687785 | 6.588330103 | 3.769170522 | 5.234059516 | 0.720123741 | -0.47368326 | 0.312459531 | 0.505206223 | 1.229187839 |
| HMDB0001852 | all-Retinoic acid | 38.06872359 | 46.22528511 | 41.58539669 | 37.26921292 | 19.15206157 | 43.27697211 | 41.9598018 | 33.23274887 | 1.262604004 | 0.336402232 | 0.316282129 | 0.499925347 | 1.078347564 |
| HMDB0061784 | 3-Allyl-1-cyclohexene | 71.13775096 | 17.79202011 | 13.01246237 | 8.887568908 | 6.142122766 | 13.96435819 | 33.98074448 | 9.664683289 | 3.515970825 | 1.813923099 | 0.321286299 | 0.493107794 | 1.455675785 |
| HMDB0034240 | Styrene | 131.7854894 | 103.1474498 | 135.2509695 | 109.4141282 | 66.73480183 | 125.372206 | 123.3946362 | 100.5070453 | 1.227721259 | 0.295983049 | 0.321441932 | 0.49289747 | 1.07420184 |
| HMDB0012996 | Lipoyllysine | 53.59232243 | 5.050356728 | 7.623577262 | 72.24973023 | 315.6718937 | 1.957885264 | 22.08875214 | 129.9598364 | 0.169965989 | -2.55668201 | 0.325563756 | 0.48736395 | 0.64670066 |
| HMDB0288899 | PC(PGF1alpha/2:0) | 28.62475276 | 11.90082333 | 8.611293228 | 20.94301346 | 62.67565681 | 18.0348537 | 16.37895644 | 33.88450799 | 0.483375956 | -1.04878238 | 0.32717895 | 0.485214646 | 1.22806385 |
| HMDB0031265 | 1-Nonanol | 650.5993411 | 133.3906264 | 141.0949302 | 97.33635443 | 52.80564151 | 119.0562761 | 308.3616326 | 89.73275734 | 3.436444412 | 1.780916623 | 0.329289127 | 0.482422609 | 1.438877277 |
| HMDB0040186 | 3-(Methylthio)hexanal | 8.733421567 | 20.55779033 | 26.52839207 | 14.82746576 | 4.918040121 | 15.34180894 | 18.60653466 | 11.69577161 | 1.590877052 | 0.669822344 | 0.329694981 | 0.481887664 | 0.974904818 |
| HMDB0028940 | Leucyl-Tryptophan | 18.95266431 | 13.37527185 | 13.2956337 | 30.03734899 | 104.0088042 | 16.03495143 | 15.20785662 | 50.02703487 | 0.303992764 | -1.71789111 | 0.330013856 | 0.481467826 | 1.384344 |
| HMDB0035289 | 2,5-Dimethylpyrazine | 114.4849408 | 87.95771369 | 70.25125259 | 101.7522239 | 40.64319361 | 57.18716916 | 90.89796901 | 66.52752889 | 1.366321138 | 0.450296613 | 0.33626265 | 0.473321369 | 1.133846927 |
| HMDB0242009 | N-Docosahexaenoyl Asparagine | 101.1925344 | 61.68797596 | 64.74956996 | 94.345375 | 26.52540475 | 18.70172145 | 75.87669342 | 46.52416707 | 1.630909229 | 0.705676488 | 0.340671022 | 0.467664807 | 1.268735943 |
| HMDB0013209 | Alanyltryptophan | 88.77144441 | 21.35415336 | 1.26593241 | 1.754414203 | 19.5976671 | 2.696684226 | 37.13051006 | 8.016255176 | 4.631902209 | 2.211604795 | 0.343024395 | 0.464674994 | 0.747339764 |
| HMDB0001199 | N2-Succinylornithine | 36.49593229 | 3.321978674 | 6.464019554 | 2.354546691 | 1.643411138 | 3.461323464 | 15.42731017 | 2.486427098 | 6.204609894 | 2.633340506 | 0.345538437 | 0.461503636 | 1.480350303 |
| HMDB0243997 | 1-Phytene | 548.5488366 | 745.996147 | 604.6216398 | 439.2212058 | 269.7953742 | 725.0803009 | 633.0555411 | 478.0322936 | 1.324294508 | 0.405223997 | 0.34601276 | 0.460907885 | 1.116695355 |
| HMDB0000851 | Pyridinoline | 46.2756966 | 7.1769399 | 4.631181485 | 50.86815847 | 225.5226491 | 2.643267841 | 19.36127266 | 93.01135847 | 0.208160304 | -2.26423312 | 0.346124145 | 0.460768104 | 0.707854058 |
| HMDB0038108 | Quinceoxepane | 143.9232012 | 38.7046689 | 25.09542421 | 20.28545075 | 10.35205882 | 40.26721195 | 69.24109812 | 23.63490718 | 2.929611595 | 1.550709406 | 0.348152368 | 0.458230647 | 1.264256444 |
| HMDB0259275 | Tris(2-butoxyethyl) phosphate | 0.958613486 | 1.733774104 | 1.75681582 | 8.239195726 | 0.745463085 | 44.30069644 | 1.483067803 | 17.76178508 | 0.083497678 | -3.58212012 | 0.3496295 | 0.45639193 | 1.185730681 |
| HMDB0094680 | Octaethylene glycol | 20.87258938 | 14.98637656 | 16.60349973 | 25.82006208 | 218.5589864 | 35.07625434 | 17.48748856 | 93.15176761 | 0.187731151 | -2.41326003 | 0.351235446 | 0.454401663 | 1.483108193 |
| HMDB0012252 | Linoleoyl ethanolamide | 34.56489795 | 7.8150813 | 1.997916933 | 4.621303805 | 1.507966716 | 2.091172404 | 14.79263206 | 2.740147641 | 5.398479935 | 2.432553241 | 0.352241861 | 0.453159033 | 1.219309619 |
| HMDB0002215 | 4a-Carbinolamine tetrahydrobiopterin | 9.040908011 | 10.39728641 | 26.70626445 | 15.23036377 | 1.950949391 | 7.357236557 | 15.38148629 | 8.179516573 | 1.880488431 | 0.911107431 | 0.353122839 | 0.452074193 | 1.140722332 |
| HMDB0028941 | Leucyl-Tyrosine | 27.08089026 | 12.03242771 | 8.109090457 | 13.17083735 | 113.2804201 | 31.05914269 | 15.74080281 | 52.50346671 | 0.299805019 | -1.73790356 | 0.355032506 | 0.449731882 | 1.239699449 |
| HMDB0061818 | 2,4-Dodecadiene | 93.25256887 | 63.82709951 | 80.4905832 | 72.9000422 | 35.67188896 | 79.15716959 | 79.19008386 | 62.57636692 | 1.26549507 | 0.339701888 | 0.358418945 | 0.445609043 | 1.002530534 |
| HMDB0012138 | 1-Naphthol | 54.47121905 | 53.33463563 | 61.39092143 | 105.7196668 | 21.97843849 | 211.8997342 | 56.39892537 | 113.1992798 | 0.498226892 | -1.0051252 | 0.360159146 | 0.443505552 | 0.548165554 |
| HMDB0248371 | Amylamine | 573.3175387 | 1000.61594 | 1054.628708 | 1048.29289 | 496.4375414 | 147.1513632 | 876.1873957 | 563.9605983 | 1.55363229 | 0.63564509 | 0.361440705 | 0.441962939 | 1.115076651 |
| HMDB0094801 | 2-octenoylglycine | 1.645789207 | 3.000129141 | 1.860268468 | 2.313791041 | 0.995134118 | 1.44009342 | 2.168728939 | 1.583006193 | 1.370006604 | 0.454182848 | 0.363374246 | 0.439645856 | 1.075731859 |
| HMDB0265714 | PA(22:1/20:5-OH) | 1.219001641 | 1.983340178 | 3.350811986 | 3.799602832 | 148.564313 | 15.73711102 | 2.184384601 | 56.03367562 | 0.038983425 | -4.68099533 | 0.365573784 | 0.437024956 | 1.602831042 |
| HMDB0036345 | 3-Cyclohexyldodecane | 352.9322702 | 96.75145061 | 84.74759615 | 73.19111114 | 40.95216326 | 116.3848992 | 178.1437723 | 76.84272452 | 2.318290683 | 1.213061473 | 0.366723166 | 0.435661654 | 1.180829784 |

## *Supplementary Table 1*
## Positive Mode

| HMDB_ID | Compound_name | DB]a,l]P-#1 | DB]a,l]P-#2 | DB]a,l]P-#3 | DMSO-#1 | DMSO-#2 | DMSO-#3 | AVE (DB[a,l]P) | AVE (DMSO) | FC (DB[a,l]P/ DMSO) | Log2 (FC) | T-Test | Log10 (P_value) | VIP |
|---|---|---|---|---|---|---|---|---|---|---|---|---|---|---|
| HMDB0038051 | alpha-Terpinyl anthranilate | 72.03556051 | 0.466681075 | 8.759441053 | 2.242322072 | 0.96059637 | 0.533874744 | 27.08722755 | 1.245597729 | 21.74636877 | 4.442702613 | 0.371284716 | 0.430292928 | 1.154135452 |
| HMDB0040135 | Hydroxydestruxin B | 758.8241121 | 571.3753473 | 465.3958631 | 726.2359059 | 327.7064361 | 197.6760536 | 598.5317741 | 417.2061319 | 1.434618833 | 0.520667475 | 0.372356134 | 0.429041488 | 1.139277134 |
| HMDB0302622 | Dodecatrienoic acid | 646.1834447 | 47.65814298 | 90.68716887 | 29.51449393 | 34.08658815 | 62.24158768 | 261.5095855 | 41.94755659 | 6.234203058 | 2.640205147 | 0.372660702 | 0.428686403 | 1.344873139 |
| HMDB0032959 | 1-Octen-3-yl glucoside | 15.16188638 | 30.35101313 | 32.79062244 | 27.20285054 | 6.606035667 | 20.10780329 | 26.10117399 | 17.97222983 | 1.45230582 | 0.538345282 | 0.376566995 | 0.424157747 | 0.970814065 |
| HMDB0005060 | Eicosadienoic acid | 145.9519543 | 9.268972326 | 18.48018094 | 9.582303943 | 5.189809384 | 9.939967013 | 57.90036917 | 8.237360113 | 7.028995743 | 2.813318581 | 0.37711157 | 0.423530143 | 1.335317542 |
| HMDB0028942 | Leucyl-Valine | 37.13794224 | 24.35629471 | 21.29481455 | 43.8011994 | 217.5501192 | 25.9208929 | 27.5963505 | 95.75740384 | 0.288190254 | -1.79490655 | 0.380733376 | 0.419379051 | 1.199912105 |
| HMDB0031728 | 2-Hexylidenecyclopentanone | 73.64554847 | 6.708393584 | 11.11011507 | 4.745039961 | 3.914229805 | 10.633628 | 30.48801904 | 6.430965922 | 4.740814896 | 2.245135065 | 0.381546526 | 0.418452496 | 1.256817386 |
| HMDB0004701 | 9,10-Epoxyoctadecenoic acid | 75.46997486 | 16.09112871 | 13.95627023 | 13.51695643 | 8.850893272 | 15.9411328 | 35.17245793 | 12.76966083 | 2.754376831 | 1.46172595 | 0.382088849 | 0.417835636 | 1.174534964 |
| HMDB0094679 | Mono-(2-ethyl-5-hydroxyhexyl) phthalate | 37.41975397 | 103.1422425 | 107.4655794 | 130.0700055 | 60.84534615 | 175.4081081 | 82.67585864 | 122.1078199 | 0.677072596 | -0.56261757 | 0.38312459 | 0.416659973 | 0.866598564 |
| HMDB0062769 | Epsilon-caprolactam | 26.52729074 | 9.902093695 | 7.829766489 | 24.36751287 | 101.6380097 | 11.72162735 | 14.75305031 | 45.90904997 | 0.321353858 | -1.6377653 | 0.383392321 | 0.41635659 | 1.146771772 |
| HMDB0036759 | Pregeijerene | 94.04642066 | 103.234635 | 124.0635905 | 71.50724424 | 49.15669479 | 127.5953431 | 107.1148821 | 82.75309406 | 1.294391265 | 0.372273777 | 0.384377608 | 0.41524192 | 1.098388802 |
| HMDB0034146 | Octadecanamide | 38415.36401 | 16881.55797 | 27551.84613 | 13994.67588 | 12779.17972 | 31038.55464 | 27616.25604 | 19270.80341 | 1.433061998 | 0.519101026 | 0.385126328 | 0.414396791 | 1.026686589 |
| HMDB0059834 | Pentylbenzene | 129.0757802 | 135.8554397 | 166.3924459 | 101.8532445 | 60.20165915 | 169.956715 | 143.7745553 | 110.6705396 | 1.299122204 | 0.377537147 | 0.385179849 | 0.414336441 | 1.075994957 |
| HMDB0241928 | N-Palmitoyl Leucine | 622.263555 | 60.33129945 | 45.35582028 | 35.42393197 | 24.21238237 | 42.79526331 | 242.6502249 | 34.14385922 | 7.106701775 | 2.829180159 | 0.386651452 | 0.412680355 | 1.31587806 |
| HMDB0015168 | Cerulenin | 772.1028253 | 25.54334577 | 57.57396066 | 12.16572831 | 21.98538647 | 19.03021883 | 285.0733772 | 17.72711121 | 16.08120883 | 4.007303953 | 0.38705833 | 0.412223581 | 1.438304045 |
| HMDB0001932 | Metoprolol | 537.6938403 | 43.26431935 | 68.52933885 | 37.74791128 | 27.46003353 | 58.85131074 | 216.4958328 | 41.35308518 | 5.235300628 | 2.388272384 | 0.389729357 | 0.409236879 | 1.224769052 |
| HMDB0242112 | 2-Aminooctadeca-4,14-diene-1,3-diol | 1240.045916 | 126.0702827 | 177.5536051 | 127.9089537 | 83.51999162 | 146.9677814 | 514.5566012 | 119.4655756 | 4.307153744 | 2.106734822 | 0.390144606 | 0.408774393 | 1.197640752 |
| HMDB0035654 | Phytal | 119.1673256 | 90.85603355 | 77.5246799 | 23.61305533 | 38.80684543 | 126.217199 | 95.84934635 | 62.87903326 | 1.524345102 | 0.608189556 | 0.390215275 | 0.408695734 | 1.185968389 |
| HMDB0039500 | N-Malonyltryptophan | 59.84974982 | 46.53495329 | 146.2850248 | 170.3631956 | 35.65989048 | 244.9302147 | 84.22324265 | 150.3177669 | 0.560301316 | -0.83572521 | 0.390854866 | 0.407984477 | 0.65511092 |
| HMDB0240601 | LysoPG(16:0/0:0) | 18.12391674 | 16.58159507 | 22.4879735 | 38.1602192 | 10.80129502 | 32.9195787 | 19.0644951 | 27.29369764 | 0.698494405 | -0.51767953 | 0.391232819 | 0.407564721 | 0.603872983 |
| HMDB0004236 | Prostaglandin B2 | 269.6495744 | 8.616299387 | 21.01788544 | 10.62061486 | 6.140067952 | 5.862828598 | 99.76125307 | 7.541170469 | 13.22888184 | 3.725619219 | 0.391404753 | 0.407373905 | 1.3432983 |
| HMDB0032147 | Geranylcitronellol | 290.5822919 | 23.97273874 | 18.42894222 | 15.85692046 | 7.757138482 | 17.46814043 | 110.9946576 | 13.69406646 | 8.105310279 | 3.018867415 | 0.39185113 | 0.406878896 | 1.303892693 |
| HMDB0015447 | Penbutolol | 0.45800511 | 0.622384738 | 0.907218693 | 2.120746126 | 2.034186465 | 54.25436932 | 0.66253618 | 19.4697673 | 0.034028973 | -4.87709259 | 0.392586962 | 0.406064127 | 1.593064691 |
| HMDB0005051 | Tsuzuic acid | 0.901335217 | 2.063805316 | 1.924184357 | 6.491796151 | 4.873666604 | 157.9797409 | 1.629774964 | 56.44840123 | 0.028871942 | -5.11418804 | 0.393141054 | 0.405451603 | 1.61380792 |
| HMDB0014861 | Methoxamine | 579.1529563 | 25.61347768 | 35.16163907 | 17.75036724 | 11.00135662 | 21.09045881 | 213.3093577 | 16.61406089 | 12.83908607 | 3.682470605 | 0.394783764 | 0.403640716 | 1.368176949 |
| HMDB0000163 | Maltose | 2226.807735 | 4233.547193 | 2258.765248 | 2242.116576 | 4834.496661 | 4706.255025 | 2906.373392 | 3927.622754 | 0.739982828 | -0.4344363 | 0.39524114 | 0.403137856 | 0.910179702 |
| HMDB0259807 | viloxazine | 54.36621358 | 0.937170708 | 3.029746043 | 0.95510662 | 0.756672404 | 0.34642539 | 19.44437678 | 0.686068138 | 28.34175748 | 4.824857318 | 0.395324578 | 0.403046184 | 1.43881447 |
| HMDB0000511 | Capric acid | 290.6288804 | 5.272826871 | 17.74974061 | 6.277334295 | 4.689987867 | 3.543778873 | 104.5504826 | 4.837033678 | 21.61458646 | 4.433933329 | 0.396300045 | 0.401975879 | 1.348373633 |
| HMDB0302287 | Nonadecenoic acid | 0.665198887 | 1.114728959 | 1.169226714 | 4.015047618 | 3.368613072 | 123.1208628 | 0.98305152 | 43.50150783 | 0.022598102 | -5.46765457 | 0.397341315 | 0.400836275 | 1.621731502 |
| HMDB0031046 | Heptadecenoic acid | 0.373496716 | 2.086957299 | 2.24733559 | 9.564155364 | 5.082117288 | 267.8287898 | 1.569263202 | 94.15835415 | 0.016666213 | -5.90692985 | 0.398020386 | 0.400094683 | 1.635464223 |
| HMDB0002080 | Petroselinic acid | 0.378080266 | 1.18945885 | 1.518526895 | 5.502679442 | 4.609427984 | 191.3913485 | 1.028688671 | 67.16781863 | 0.015315201 | -6.02889185 | 0.398493098 | 0.399579196 | 1.674746115 |
| HMDB0000383 | 3a,17a-Dihydroxy-5b-androstane | 72.82308763 | 0.652968161 | 4.387859964 | 1.69048798 | 0.948686435 | 0.446131671 | 25.95463859 | 1.028435362 | 25.23701493 | 4.657469371 | 0.399319655 | 0.398679313 | 1.223623447 |
| HMDB0304813 | Xestoaminol C | 501.9868954 | 670.9738053 | 851.5350539 | 585.5375823 | 363.0997715 | 684.8165904 | 674.8319182 | 544.4846481 | 1.239395676 | 0.30963684 | 0.400478766 | 0.397420506 | 0.945148752 |
| HMDB0242035 | N-Lauroyl Leucine | 598.2371767 | 106.068422 | 119.9814303 | 107.8698142 | 61.57701901 | 140.7538456 | 274.762343 | 103.4002262 | 2.657270231 | 1.40994495 | 0.400711445 | 0.397168253 | 1.085603789 |
| HMDB0030039 | Jasmolone | 693.1619265 | 23.26849763 | 51.63200488 | 25.72833394 | 19.08717222 | 29.34805736 | 256.0208097 | 24.72118784 | 10.35633123 | 3.372441108 | 0.401147969 | 0.396695403 | 1.201310445 |
| HMDB0062477 | 2-Deoxyglucose | 2.79562735 | 7.146643608 | 2.493332831 | 5.689369169 | 402.6178723 | 17.47097196 | 4.145201263 | 141.9260712 | 0.029206764 | -5.09755367 | 0.401444086 | 0.396374935 | 1.434710181 |
| HMDB0255916 | Octylamine | 146.7402143 | 5.479850247 | 11.34311411 | 5.396317686 | 4.38117264 | 7.545299752 | 54.52105957 | 5.774263359 | 9.442080518 | 3.239104786 | 0.401516854 | 0.39629622 | 1.193614522 |
| HMDB0116637 | PG(a-13:0/18:2) | 2.41385408 | 5.079147975 | 11.84314524 | 8.571932234 | 936.2293743 | 38.00721139 | 6.445382432 | 327.6028393 | 0.019674379 | -5.66753809 | 0.402077148 | 0.39569061 | 1.45017789 |
| HMDB0297036 | DG(18:3(9,11,15)-OH(13)/2:0/0:0) | 3.600467994 | 7.50112679 | 5.018379663 | 3.946812779 | 1084.261017 | 46.0191084 | 5.373324816 | 378.075646 | 0.014212301 | -6.13671606 | 0.402086858 | 0.395680122 | 1.319618009 |
| HMDB0000077 | Dehydroepiandrosterone | 0.569757771 | 1.005382686 | 1.392245795 | 2.865152577 | 1.486020754 | 67.35074532 | 0.989128751 | 23.90063955 | 0.041385033 | -4.59474709 | 0.40226119 | 0.395491865 | 1.403593112 |
| HMDB0029968 | Ethyl betaglucopyranoside | 6.788691105 | 9.444472239 | 5.919404695 | 14.00192423 | 626.8873647 | 22.63001134 | 7.384189346 | 221.1731001 | 0.033386471 | -4.90459257 | 0.402488929 | 0.395246061 | 1.452576595 |
| HMDB0037606 | 13-Nor-6-eremophilene-8,11-dione | 45.82057025 | 3.51517715 | 2.378668056 | 2.309912268 | 1.99947159 | 2.295618099 | 17.23813849 | 2.201667319 | 7.829583669 | 2.968935596 | 0.403196618 | 0.394483119 | 1.177948403 |
| HMDB0037326 | Traumatin | 402.8132299 | 10.11485706 | 24.07940595 | 9.574073579 | 8.312787481 | 13.45747078 | 145.6691643 | 10.44811061 | 13.94215372 | 3.801381535 | 0.403442884 | 0.394217941 | 1.222562967 |
| HMDB0034450 | Neocnidilide | 234.440487 | 3.603551088 | 15.99002574 | 4.644874219 | 6.345290901 | 6.773755355 | 84.67802129 | 5.921306825 | 14.300563 | 3.83800004 | 0.403676551 | 0.393966478 | 1.091897879 |
| HMDB0032638 | 5-Hexadecenoic acid | 1.841999801 | 13.74848156 | 4.755717654 | 16.18934422 | 10.23343177 | 395.9446291 | 6.782066337 | 140.789135 | 0.048171802 | -4.3756673 | 0.403774147 | 0.393861491 | 1.408878855 |
| HMDB0028980 | Methionyl-Phenylalanine | 15.34767267 | 11.90546506 | 9.050806092 | 27.51739768 | 228.7566096 | 4.251813161 | 12.10131461 | 86.84194016 | 0.139348736 | -2.84322818 | 0.404384818 | 0.393205157 | 0.81869685 |
| HMDB0040284 | 1,2-Dihydro-1,1,6-trimethylnaphthalene | 227.5939246 | 39.35758207 | 36.4799855 | 27.87920124 | 15.09648714 | 61.16902021 | 101.1438307 | 34.71490286 | 2.913556495 | 1.542781285 | 0.404491443 | 0.393090661 | 1.099837428 |
| HMDB0003128 | Cortolone | 3.35668162 | 3.184819217 | 6.365281158 | 3.397541804 | 692.4009542 | 26.42197486 | 4.302260665 | 240.7401569 | 0.017870972 | -5.80623806 | 0.40515714 | 0.392376504 | 1.298841132 |
| HMDB0028933 | Leucylleucine | 20.57058751 | 2.346222617 | 2.290125865 | 22.72536107 | 30.93769319 | 1.380354538 | 8.402311996 | 18.34780293 | 0.457946492 | -1.12674905 | 0.405476774 | 0.392034017 | 0.632172184 |
| HMDB0028929 | Leucyl-Glycine | 35.97106603 | 3.67211153 | 4.631723938 | 30.34241044 | 102.2379957 | 0.701392496 | 14.7583005 | 44.42726621 | 0.332190156 | -1.58991877 | 0.405761725 | 0.391728922 | 0.278223955 |
| HMDB0303184 | Acetolein | 25.35396585 | 35.79481879 | 42.27592551 | 36.93606879 | 5114.836414 | 181.6609739 | 34.47490339 | 1777.811152 | 0.019391769 | -5.68841176 | 0.405892684 | 0.391588776 | 1.355678962 |

## *Supplementary Table 1*
## Positive Mode

| HMDB_ID | Compound_name | DB]a,l]P-#1 | DB]a,l]P-#2 | DB]a,l]P-#3 | DMSO-#1 | DMSO-#2 | DMSO-#3 | AVE (DB[a,l]P) | AVE (DMSO) | FC (DB[a,l]P/ DMSO) | Log2 (FC) | T-Test | Log10 (P_value) | VIP |
|---|---|---|---|---|---|---|---|---|---|---|---|---|---|---|
| HMDB0041081 | 1,5-Tridecadiene | 32.19693503 | 34.89014641 | 49.13289702 | 28.52871263 | 14.98443354 | 44.68808723 | 38.73999282 | 29.40041113 | 1.317668404 | 0.397987357 | 0.406008659 | 0.391464704 | 1.023053989 |
| HMDB0000865 | Muricholic acid | 4.04779382 | 6.966640616 | 10.94892104 | 5.917763106 | 2.712894204 | 6.797326457 | 7.321118492 | 5.142661256 | 1.423605042 | 0.509548947 | 0.407094491 | 0.390304774 | 0.890256969 |
| HMDB0253021 | Isoleucyl-prolyl-proline | 81.02476296 | 6.699542343 | 5.718333474 | 62.47675243 | 213.7050999 | 3.995478639 | 31.14754626 | 93.39244365 | 0.333512488 | -1.58418731 | 0.407206544 | 0.390185251 | 0.692399839 |
| HMDB0039827 | Serratol | 55.12348056 | 1.287686973 | 1.875088468 | 0.558583966 | 0.978083488 | 1.052999871 | 19.428752 | 0.863222442 | 22.50723691 | 4.492317051 | 0.407488196 | 0.389884967 | 1.313172969 |
| HMDB0263012 | PA(14:0/18:1-O(12,13)) | 2.738545178 | 7.315451345 | 15.8264533 | 9.817120898 | 1931.218304 | 57.82545965 | 8.626816609 | 666.2869617 | 0.012947599 | -6.27117156 | 0.407712261 | 0.389646228 | 1.409360027 |
| HMDB0060055 | Tetranor 12-HETE | 38.37687245 | 1.373861035 | 2.164961357 | 0.875269398 | 1.429633817 | 1.608960954 | 13.97189828 | 1.30462139 | 10.70954255 | 3.420824952 | 0.408335196 | 0.388983185 | 1.172781196 |
| HMDB0030197 | 3-Oxo-2-(2-pentenyl)-1-cyclopenteneacetic acid | 164.1917415 | 2.447724887 | 5.777251206 | 2.01237619 | 1.737790431 | 2.632385412 | 57.47223918 | 2.127517344 | 27.01375824 | 4.755622461 | 0.408658147 | 0.388639839 | 1.276096614 |
| HMDB0296941 | DG(2:0/0:0/20:3-2OH(5,6)) | 3.050504548 | 5.344455422 | 7.021272146 | 3.537234781 | 1231.017157 | 33.89610946 | 5.138744039 | 422.8168339 | 0.012153594 | -6.36247321 | 0.410023046 | 0.387191733 | 1.259646229 |
| HMDB0028893 | Histidylproline | 3.376679957 | 7.640313845 | 3.968233846 | 3.157312255 | 524.4536245 | 18.1037549 | 4.995075883 | 181.9048972 | 0.027459821 | -5.18653398 | 0.410314567 | 0.386883065 | 1.171988456 |
| HMDB0001937 | Salbutamol | 328.4799068 | 4.337049755 | 11.58387755 | 3.48325767 | 4.555796029 | 5.387208888 | 114.800278 | 4.475420862 | 25.65128097 | 4.680958968 | 0.410371723 | 0.386822572 | 1.216438369 |
| HMDB0000910 | Tridecanoic acid | 901.6364907 | 30.41691551 | 49.43002107 | 36.38158279 | 12.56600234 | 42.94270633 | 327.1611424 | 30.63009715 | 10.68103509 | 3.416979559 | 0.410474479 | 0.386713839 | 1.16117958 |
| HMDB0040326 | 7-Hydroxy-2,5-dimethyl-4H-1-benzopyran-4-one | 2.868173756 | 6.409359759 | 6.742592354 | 5.31072457 | 460.8855288 | 14.46475666 | 5.340041957 | 160.2203367 | 0.033329364 | -4.90706239 | 0.411240996 | 0.385903597 | 1.244531693 |
| HMDB0060379 | 3-Polyprenyl-4-hydroxy-5-methoxybenzoate | 0.920574149 | 0.882819669 | 0.813782996 | 1.411671364 | 47.35583132 | 1.111264171 | 0.872392272 | 16.62625562 | 0.05247076 | -4.2523425 | 0.413031945 | 0.384016357 | 1.203167829 |
| HMDB0029066 | Threonyllysine | 10.91269498 | 1.464276171 | 0.606075997 | 9.028200055 | 74.64442662 | 0.931511409 | 4.327682384 | 28.20137936 | 0.153456408 | -2.7040992 | 0.414217948 | 0.382771087 | 0.927648814 |
| HMDB0243957 | 1-Naphthamide | 53.51543598 | 311.6563762 | 280.7099482 | 212.4293167 | 109.5903527 | 74.6259289 | 215.2939201 | 132.2151994 | 1.628359833 | 0.70341954 | 0.414259317 | 0.382727715 | 0.571874401 |
| HMDB0013286 | N-Undecanoylglycine | 2327.450811 | 454.0727362 | 626.0490624 | 212.8007 | 248.1601885 | 1123.135644 | 1135.857536 | 528.0321776 | 2.15111424 | 1.105084144 | 0.414267051 | 0.382719607 | 1.053418324 |
| HMDB0093783 | DG(a-17:0/0:0/i-16:0) | 6.196362182 | 7.089335974 | 4.616675295 | 10.57627889 | 3.182802864 | 11.69177403 | 5.967457817 | 8.483618594 | 0.703409489 | -0.5075633 | 0.41445579 | 0.382521788 | 0.559991932 |
| HMDB0015680 | Nonoxynol-9 | 3.322218091 | 9.846181503 | 20.31916131 | 14.29830023 | 3974.859035 | 60.47085807 | 11.1625203 | 1349.876065 | 0.008269293 | -6.91802035 | 0.415056317 | 0.381892972 | 1.382741257 |
| HMDB0041366 | 24,25-Diacetylvulgaroside | 1.195703313 | 2.021819347 | 3.91945249 | 1.569231755 | 522.9459473 | 9.527943127 | 2.378991717 | 178.0143741 | 0.013364043 | -6.22549968 | 0.415675073 | 0.381246018 | 1.187467896 |
| HMDB0247848 | Lysyl-aspartyl-glutamyl-leucine | 84.9993245 | 6.670934893 | 5.928424501 | 65.84363066 | 207.7107602 | 3.260841401 | 32.53289463 | 92.2717441 | 0.352576999 | -1.50398973 | 0.41610084 | 0.380801407 | 0.612721261 |
| HMDB0028868 | Hydroxyprolyl-Lysine | 119.1062209 | 453.8313325 | 491.0204035 | 393.1058615 | 203.3435447 | 46.19668586 | 354.6526523 | 214.215364 | 1.655589243 | 0.72734478 | 0.41632332 | 0.380569262 | 0.881638837 |
| HMDB0004437 | Diethanolamine | 13.56826093 | 13.42595372 | 12.21673223 | 14.66224404 | 1670.082989 | 27.8273665 | 13.07031563 | 570.8575331 | 0.022895933 | -5.44876486 | 0.416976678 | 0.379888235 | 1.197320667 |
| HMDB0248234 | alpha-Methyltyrosine methyl ester | 93.25579141 | 123.0696995 | 162.6678564 | 174.5793053 | 81.67150702 | 313.1381711 | 126.3311158 | 189.7963278 | 0.66561412 | -0.58724206 | 0.417033304 | 0.379829261 | 0.719796452 |
| HMDB0303805 | 3-Oxo-alpha-ionol | 1560.623916 | 309.4776625 | 342.0871924 | 140.0802564 | 143.6079839 | 702.4574896 | 737.3962569 | 328.7152433 | 2.243267606 | 1.165601734 | 0.417179 | 0.379677561 | 1.046267144 |
| HMDB0000238 | Sepiapterin | 24109.60587 | 24570.976 | 29300.79699 | 43180.04001 | 9386.108228 | 84351.21242 | 25993.79296 | 45639.12022 | 0.569550702 | -0.81210382 | 0.417259994 | 0.379593252 | 0.38323761 |
| HMDB0112100 | 5-Butyl-3,4-dimethyl-2-furannonanoic acid | 6.182674544 | 16.78102357 | 9.38606251 | 7.693974367 | 3999.979015 | 50.92065239 | 10.78325354 | 1352.864547 | 0.007970682 | -6.97108105 | 0.417317537 | 0.379533364 | 1.215756822 |
| HMDB0030180 | Crustecdysone | 0.376854221 | 0.483392341 | 0.509849477 | 0.362940435 | 602.2739182 | 6.053534611 | 0.45669868 | 202.8967977 | 0.002250892 | -8.79528776 | 0.417395582 | 0.379452152 | 1.314458717 |
| HMDB0248243 | 1-Tetralone | 14.46095115 | 18.76753452 | 21.48934579 | 16.53329503 | 342.6712903 | 22.66448826 | 18.23927715 | 127.2896912 | 0.143289507 | -2.80299512 | 0.417876642 | 0.378951904 | 1.034215749 |
| HMDB0029771 | 7,8-Dihydrovomifoliol 9-[apiosyl-(1->6)-glucoside] | 13.14877617 | 7.201748538 | 4.647190162 | 15.11311762 | 72.47205338 | 2.652404865 | 8.332571624 | 30.07919196 | 0.277021126 | -1.85193209 | 0.418448787 | 0.378357686 | 0.665285274 |
| HMDB0297205 | DG(8:0/0:0/20:5-3OH(5,6,15)) | 6.518808135 | 14.24599667 | 26.42926389 | 16.55081939 | 11022.85257 | 86.23666109 | 15.73135623 | 3708.546683 | 0.004241919 | -7.88106707 | 0.418920523 | 0.377868363 | 1.304085198 |
| HMDB0032591 | Dehydrozingerone | 4.527599769 | 1.49863127 | 1.221466669 | 6.344222183 | 399.284304 | 0.8503733 | 2.415899236 | 135.4929665 | 0.01783044 | -5.80951387 | 0.419246091 | 0.377530978 | 0.989096456 |
| HMDB0011569 | MG(18:3/0:0/0:0) | 58.54970949 | 64.37671297 | 51.84886221 | 38.37617531 | 47307.38306 | 316.7757063 | 58.25842823 | 15887.51165 | 0.003666932 | -8.09121069 | 0.41974252 | 0.377017035 | 1.182246538 |
| HMDB0033910 | Acetophenone | 39.37202522 | 37.45854765 | 41.33594825 | 32.42596433 | 5971.517529 | 68.70741056 | 39.38884037 | 2024.216968 | 0.019458804 | -5.68343318 | 0.420481422 | 0.376253188 | 1.067150188 |
| HMDB0010697 | Cer(t18:0/16:0) | 26.96972337 | 43.3432381 | 40.44020549 | 38.48772631 | 10.19100876 | 34.64477922 | 36.91772232 | 27.77450476 | 1.329194621 | 0.410552359 | 0.42056779 | 0.376163992 | 0.929352636 |
| HMDB0014597 | Meperidine | 162.2444358 | 2.19006057 | 2.135319905 | 1.690542653 | 1.407192696 | 2.527051571 | 55.5232721 | 1.874928973 | 29.61353357 | 4.888184742 | 0.420580118 | 0.376151262 | 1.056938568 |
| HMDB0244162 | 1,3-Di-o-tolylguanidine | 0.403124991 | 71.96967612 | 0.617647026 | 0.389924272 | 0.272452417 | 0.517686126 | 24.33014938 | 0.393354271 | 61.85301939 | 5.950772118 | 0.420764675 | 0.375960728 | 1.097753602 |
| HMDB0242008 | N-Docosahexaenoyl Arginine | 16.69341645 | 23.51810765 | 36.39744785 | 24.66945247 | 33287.0805 | 131.245777 | 25.53632398 | 11147.66524 | 0.002290733 | -8.76997501 | 0.420832813 | 0.375890405 | 1.241524374 |
| HMDB0260512 | MG(20:3(6,8,11)-OH(5)/0:0/0:0) | 40.52973984 | 57.08851026 | 47.14939069 | 38.33031188 | 70618.27852 | 263.8731328 | 48.25588026 | 23640.16066 | 0.002041267 | -8.93631937 | 0.420969627 | 0.375749238 | 1.205396646 |
| HMDB0302461 | 5-Hydroxycapsanthin | 0.369250732 | 0.497850828 | 0.506478202 | 0.38237971 | 120.8359198 | 0.794488426 | 0.457859921 | 40.67092931 | 0.01125767 | -6.47294786 | 0.421399617 | 0.375305863 | 1.063961199 |
| HMDB0002157 | 27-Norcholestanehexol | 0.726188136 | 0.987971276 | 1.508229284 | 0.758822018 | 2544.988702 | 5.505485775 | 1.074129565 | 850.41767 | 0.001263061 | -9.62885974 | 0.421716462 | 0.374979446 | 1.170344999 |
| HMDB0303749 | 7-Epi-12-hydroxyjasmonic acid | 1.206347293 | 1.074343444 | 1.035375163 | 0.850987749 | 1896.875716 | 3.314512361 | 1.1053553 | 633.6804054 | 0.001744342 | -9.16310142 | 0.422054804 | 0.374631152 | 1.112659957 |
| HMDB0062290 | Hydroxy--eicosatrienoic acid | 0.649269917 | 0.832820988 | 0.892486415 | 0.625298305 | 523.4271649 | 1.452642038 | 0.791525773 | 175.1683684 | 0.004518657 | -7.78989024 | 0.422103526 | 0.37458102 | 1.052530239 |
| HMDB0000546 | Epietiocholanolone | 1.642715734 | 2.261815672 | 1.627834965 | 2.347857096 | 483.6967827 | 1.795591034 | 1.844122124 | 162.6134103 | 0.011340529 | -6.46236823 | 0.42210465 | 0.374579863 | 1.036744792 |
| HMDB0242645 | 24,24-Difluoro-1alpha,25-dihydroxyvitamin D3 | 0.596103893 | 0.818489618 | 0.984014951 | 0.633137707 | 77.0710287 | 1.029440146 | 0.799536154 | 26.24453552 | 0.030464862 | -5.03670998 | 0.422173253 | 0.374509285 | 0.994236951 |
| HMDB0251534 | Dmap-ethyl-paf | 1.048468493 | 1.207317463 | 1.507598122 | 1.122241106 | 548.6172161 | 1.82547111 | 1.254461359 | 183.8549761 | 0.006823103 | -7.19535638 | 0.42218724 | 0.374494897 | 1.039219982 |
| HMDB0010318 | Pregnanediol 3-O-glucuronide | 0.353468769 | 0.508435815 | 0.542189304 | 0.416551162 | 126.4420263 | 0.607530151 | 0.468031296 | 42.48870255 | 0.011015429 | -6.50433048 | 0.422246759 | 0.374433675 | 1.02051839 |

## *Supplementary Table 1*
## Positive Mode

| HMDB_ID | Compound_name | DB]a,l]P-#1 | DB]a,l]P-#2 | DB]a,l]P-#3 | DMSO-#1 | DMSO-#2 | DMSO-#3 | AVE (DB[a,l]P) | AVE (DMSO) | FC (DB[a,l]P/ DMSO) | Log2 (FC) | T-Test | Log10 (P_value) | VIP |
|---|---|---|---|---|---|---|---|---|---|---|---|---|---|---|
| HMDB0029849 | Carveol glucoside | 0.914041958 | 0.917808785 | 0.884518285 | 0.726996601 | 984.2012686 | 1.238873774 | 0.905456343 | 328.7223796 | 0.002754471 | -8.50400888 | 0.422558789 | 0.374112861 | 1.002432415 |
| HMDB0260463 | MG(20:3-O(8,9)/0:0/0:0) | 0.332156257 | 0.401724287 | 0.424947526 | 0.324442623 | 138.3908595 | 0.465349815 | 0.386276023 | 46.39355066 | 0.008326072 | -6.90814832 | 0.422577762 | 0.374093361 | 0.98971909 |
| HMDB0092961 | DG(10:0/10:0/0:0) | 1.05101706 | 0.936010131 | 0.902059436 | 0.741413892 | 491.3196373 | 1.166937837 | 0.963028876 | 164.4093297 | 0.005857507 | -7.4154974 | 0.422670688 | 0.373997869 | 0.97431805 |
| HMDB0010400 | LysoPC(22:2/0:0) | 0.481401933 | 0.541149901 | 0.640991822 | 0.477678423 | 105.1712091 | 0.626425074 | 0.554514552 | 35.42510419 | 0.015653152 | -5.99740296 | 0.422677228 | 0.373991149 | 0.981489354 |
| HMDB0248604 | Arlacel A | 0.602929865 | 0.773380429 | 0.815709256 | 0.580669169 | 220.7736656 | 0.867227354 | 0.730673183 | 74.07385404 | 0.009864117 | -6.66359433 | 0.422685276 | 0.373982881 | 0.975622253 |
| HMDB0242012 | N-Docosahexaenoyl Glutamine | 0.497071261 | 0.679542354 | 0.937410143 | 0.541156656 | 196.6492755 | 0.851910841 | 0.704674586 | 66.01411435 | 0.010674605 | -6.54967352 | 0.422698116 | 0.373969688 | 0.977719654 |
| HMDB0258186 | Scymnol | 1.057656895 | 0.941923406 | 0.907758227 | 0.746097799 | 724.7739112 | 1.114974488 | 0.969112842 | 242.2116611 | 0.004001099 | -7.96538795 | 0.422711313 | 0.373956129 | 0.966081583 |
| HMDB0030012 | Scutigeral | 0.47462569 | 0.56629982 | 0.617201373 | 0.473364557 | 83.77532272 | 0.619148263 | 0.552708961 | 28.28927851 | 0.019537754 | -5.67759157 | 0.422739733 | 0.373926931 | 0.977730177 |
| HMDB0260464 | MG(20:3-O(5,6)/0:0/0:0) | 0.485970793 | 0.579836235 | 0.6319545 | 0.484679515 | 76.53515085 | 0.633947927 | 0.565920509 | 25.88459276 | 0.021863219 | -5.51535039 | 0.422750748 | 0.373915615 | 0.977468566 |
| HMDB0041376 | exo,exo-1,8-Epoxy-p-menthane-2,6-diol | 1.181116767 | 1.051873753 | 1.013720486 | 0.8331895 | 276.062395 | 1.245125019 | 1.082237002 | 92.71356982 | 0.011672908 | -6.42069213 | 0.422830974 | 0.373833207 | 0.961689483 |
| HMDB0251082 | Dexamethasone palmitate | 0.553344117 | 0.709776601 | 0.748624249 | 0.53291417 | 40.51041615 | 0.795905431 | 0.670581656 | 13.94641192 | 0.048082737 | -4.37833717 | 0.422835166 | 0.373828901 | 0.969788355 |
| HMDB0297258 | DG(PGF1alpha/0:0/8:0) | 0.454169912 | 0.497196175 | 0.587667992 | 0.384495021 | 214.52273 | 0.570757827 | 0.513011359 | 71.82599429 | 0.007142419 | -7.12937148 | 0.422840781 | 0.373823134 | 0.95132986 |
| HMDB0296999 | DG(2:0/20:4-2OH/0:0) | 0.620631704 | 0.621647663 | 0.610635458 | 0.509303335 | 107.6942232 | 0.68793699 | 0.617638275 | 36.29715451 | 0.017016162 | -5.87695048 | 0.42285524 | 0.373808284 | 0.967301199 |
| HMDB0262749 | PA(20:3-O(5,6)/12:0) | 0.362332674 | 0.481247114 | 0.514470713 | 0.363388543 | 66.29214651 | 0.515338524 | 0.452683501 | 22.39029119 | 0.020217848 | -5.62822676 | 0.422884185 | 0.373778556 | 0.967075983 |
| HMDB0011566 | MG(18:1/0:0/0:0) | 1.261899293 | 1.123816698 | 1.083053938 | 0.890175528 | 181.5765115 | 1.330285393 | 1.156256643 | 61.26565747 | 0.018872835 | -5.72754504 | 0.422945207 | 0.373715892 | 0.959058496 |
| HMDB0037961 | gamma-Eudesmol rhamnoside | 0.672230155 | 0.923589848 | 1.379467359 | 0.784890696 | 241.8964757 | 1.02911858 | 0.991762454 | 81.23682831 | 0.012208286 | -6.3559955 | 0.423056294 | 0.37360184 | 0.954895953 |
| HMDB0263424 | PA(18:1-2OH(9,10)/16:1) | 0.846356128 | 1.01340751 | 1.216979736 | 0.80043211 | 175.6028218 | 1.121636486 | 1.025581125 | 59.17496348 | 0.017331335 | -5.85047339 | 0.42307727 | 0.373580306 | 0.951793588 |
| HMDB0263363 | PA(PGF1alpha/16:0) | 0.725295064 | 0.870123486 | 0.910124982 | 0.687260328 | 123.7087627 | 0.888261455 | 0.835181178 | 41.76142816 | 0.019998865 | -5.64393804 | 0.423095857 | 0.373561227 | 0.955779489 |
| HMDB0037325 | Achimilic acid | 0.684946156 | 0.717800242 | 0.754860523 | 0.692073222 | 155.517708 | 0.618465846 | 0.719202307 | 52.27608235 | 0.01375777 | -6.18360957 | 0.423126762 | 0.373529505 | 0.944895645 |
| HMDB0010222 | 9-HETE | 0.622262938 | 0.638617173 | 0.861092232 | 0.499945867 | 232.7225982 | 0.712667238 | 0.707324115 | 77.97840378 | 0.009070769 | -6.78455937 | 0.423152721 | 0.373502862 | 0.91905816 |
| HMDB0244855 | 2-Nitrophenyl agalactopyranoside | 1.540892314 | 1.325090328 | 1.074834996 | 1.661681737 | 238.1883509 | 0.746681735 | 1.313605879 | 80.19890478 | 0.016379349 | -5.93197814 | 0.423185455 | 0.373469267 | 0.910258586 |
| HMDB0000404 | 2b,3a,7a-Trihydroxy-5b-cholanoic acid | 0.934835014 | 1.320310337 | 1.517563157 | 0.981562913 | 117.9263063 | 1.424169359 | 1.257569503 | 40.11067954 | 0.031352486 | -4.99527637 | 0.42319359 | 0.373460919 | 0.958265307 |
| HMDB0036873 | Heteroxanthin | 0.420659579 | 0.527455622 | 0.568571687 | 0.407895455 | 72.208535 | 0.535117512 | 0.505562296 | 24.38384932 | 0.02073349 | -5.59189321 | 0.423198791 | 0.373455582 | 0.950354504 |
| HMDB0031111 | 5,10-Pentadecadien-1-ol | 1.507021126 | 1.275315548 | 2.551732366 | 1.01017781 | 527.3491596 | 2.022874113 | 1.778023013 | 176.7940705 | 0.010057029 | -6.63565208 | 0.423224992 | 0.373428695 | 0.90525704 |
| HMDB0250270 | Ciprostene | 1.404663207 | 1.219671632 | 1.782882709 | 1.210747536 | 150.4411653 | 1.567723638 | 1.469072516 | 51.07321217 | 0.028764052 | -5.11958928 | 0.423269691 | 0.373382829 | 0.956254405 |
| HMDB0039148 | Ganodosterone | 0.49755874 | 0.429634706 | 0.421994321 | 0.340254641 | 138.7590292 | 0.405976721 | 0.449729256 | 46.50175353 | 0.009671232 | -6.69208457 | 0.423289566 | 0.373362436 | 0.90988312 |
| HMDB0297052 | DG(20:3(6,8,11)-OH(5)/2:0/0:0) | 0.521845565 | 0.450606025 | 0.442592697 | 0.356863142 | 110.8372858 | 0.425793246 | 0.471681429 | 37.20664739 | 0.012677343 | -6.30160379 | 0.423490795 | 0.373156025 | 0.906017909 |
| HMDB0033709 | Desglucocoroloside | 0.628927602 | 0.712686196 | 0.908300869 | 0.569013204 | 78.03410286 | 0.807083535 | 0.749971555 | 26.47006653 | 0.028332817 | -5.14138213 | 0.423576793 | 0.373067842 | 0.936513917 |
| HMDB0002823 | Docosatrienoic acid | 114.74134 | 10.50547569 | 11.81348047 | 12.10844658 | 5.829570029 | 15.7986442 | 45.68676537 | 11.24555361 | 4.062651513 | 2.022421618 | 0.423738602 | 0.37290197 | 0.986256061 |
| HMDB0253942 | Lactamide | 2.90869248 | 3.60077353 | 3.806269831 | 2.942642574 | 509.0356542 | 2.977163619 | 3.438578614 | 171.6518201 | 0.020032288 | -5.64152902 | 0.423743497 | 0.372896953 | 0.917137076 |
| HMDB0255591 | Nicotyrine | 35.46110073 | 3.23054098 | 5.837375708 | 47.17921127 | 32.61854295 | 6.684682175 | 14.84300581 | 28.8274788 | 0.514890876 | -0.95766139 | 0.423922176 | 0.372713864 | 0.974334498 |
| HMDB0262991 | PA(20:4-2OH/14:0) | 0.531856745 | 0.527860051 | 0.544138719 | 0.381595724 | 47.67965234 | 0.573964936 | 0.534618505 | 16.21173767 | 0.032977249 | -4.92238515 | 0.424045076 | 0.372587975 | 0.915256447 |
| HMDB0010355 | Cholestane-3,7,12,25-tetrol-3-glucuronide | 0.287516587 | 0.399485349 | 0.417660825 | 0.273739735 | 12.2364726 | 0.427784778 | 0.36822092 | 4.312665703 | 0.085381281 | -3.54993639 | 0.424375214 | 0.372249989 | 0.937190828 |
| HMDB0038540 | Goshonoside F2 | 0.662312478 | 0.684769355 | 0.859989755 | 0.523355063 | 76.59819042 | 0.683006444 | 0.735690529 | 25.93485064 | 0.02836687 | -5.13964923 | 0.42466913 | 0.371949307 | 0.882011333 |
| HMDB0241823 | 10-Nitrooctadec-9-enoylcarnitine | 0.820297439 | 0.864455284 | 0.989094876 | 0.701578029 | 25.57686552 | 0.985124132 | 0.891282533 | 9.087855895 | 0.098074017 | -3.34998522 | 0.424913425 | 0.371699548 | 0.935656698 |
| HMDB0029668 | Eremopetasinorol | 230.7691026 | 31.95860887 | 36.60911994 | 27.09277743 | 15.19468631 | 61.14118731 | 99.7789438 | 34.47621702 | 2.894138407 | 1.533133918 | 0.424953521 | 0.371658568 | 1.008736391 |
| HMDB0302460 | 5-Hydroxycapsanthin-5,6-epoxide | 0.527600504 | 1.586702347 | 0.741702218 | 0.486586337 | 93.82782161 | 0.686541042 | 0.95200169 | 31.666983 | 0.030062911 | -5.05587148 | 0.427204203 | 0.369364483 | 0.779992853 |
| HMDB0094711 | 9-Oxo-nonanoic acid | 13.99309874 | 25.30283802 | 18.50221384 | 16.55292193 | 13.15731796 | 18.4796037 | 19.2660502 | 16.0632812 | 1.199384482 | 0.262294212 | 0.428225582 | 0.368327392 | 0.840443792 |
| HMDB0015064 | Misoprostol | 0.464306443 | 0.606436215 | 52.97158439 | 1.09048757 | 2943.941032 | 4.190951387 | 18.01410902 | 983.074157 | 0.018324263 | -5.77010105 | 0.428737441 | 0.367808588 | 0.790088589 |
| HMDB0061122 | 4-Hydroxy-alprenolol | 62.34443825 | 10.83096189 | 8.386732818 | 3.814050617 | 4.570961835 | 23.7643468 | 27.18737765 | 10.71645308 | 2.536975382 | 1.34310952 | 0.429632709 | 0.366902662 | 0.980320293 |
| HMDB0301955 | Arctiol | 193.5931431 | 247.2348439 | 146.2253013 | 132.6862252 | 82.03549063 | 232.7646307 | 195.6844294 | 149.1621155 | 1.31189095 | 0.391647802 | 0.429886797 | 0.366645893 | 0.974706224 |
| HMDB0042008 | Ritalinic acid | 0.852453421 | 0.747891095 | 1.512650942 | 0.640146618 | 44.15531585 | 0.812149681 | 1.037665152 | 15.20253738 | 0.06825605 | -3.87289926 | 0.431028533 | 0.365493979 | 0.778207214 |
| HMDB0032532 | 2-DECENOL | 0.385416843 | 0.824101011 | 13.3786387 | 3.545618164 | 3.051777841 | 205.9644241 | 4.86271885 | 70.85394004 | 0.068630183 | -3.86501299 | 0.431733639 | 0.364784112 | 1.123943009 |
| HMDB0013059 | (9-hydroxy-PGA1)-glutathione | 527.6453411 | 37.33588959 | 11.41620519 | 391.6032292 | 1188.253107 | 2.545021352 | 192.1324786 | 527.4671193 | 0.364254892 | -1.45697975 | 0.435386141 | 0.3611254 | 0.282829032 |
| HMDB0242381 | Cholylaspartic acid | 16.56176092 | 3.619051377 | 2.92648562 | 9.987749548 | 71.57813271 | 4.165699531 | 7.702432641 | 28.57719393 | 0.269530754 | -1.8914782 | 0.436388058 | 0.360127143 | 0.95195915 |
| HMDB0031126 | 3,4-Dimethyl-5-pentyl-2-furanundecanoic acid | 76.55026412 | 8.652143772 | 8.214162441 | 6.538497953 | 5.903927272 | 16.55401609 | 31.13885678 | 9.665480437 | 3.221656386 | 1.687802628 | 0.444740108 | 0.351893703 | 0.874641079 |
| HMDB0001337 | Leukotriene A4 | 203.3385077 | 3.697659524 | 4.044757817 | 5.183801163 | 7.207153505 | 11.72903332 | 70.36030834 | 8.039995997 | 8.751286488 | 3.129495117 | 0.447568737 | 0.349140257 | 0.495178271 |

## *Supplementary Table 1*
## Positive Mode

| HMDB_ID | Compound_name | DB]a,l]P-#1 | DB]a,l]P-#2 | DB]a,l]P-#3 | DMSO-#1 | DMSO-#2 | DMSO-#3 | AVE (DB[a,l]P) | AVE (DMSO) | FC (DB[a,l]P/ DMSO) | Log2 (FC) | T-Test | Log10 (P_value) | VIP |
|---|---|---|---|---|---|---|---|---|---|---|---|---|---|---|
| HMDB0001551 | Palmitaldehyde | 135.149174 | 146.6349688 | 209.9252278 | 149.221137 | 95.00645081 | 167.2410929 | 163.9031236 | 137.1562269 | 1.195010444 | 0.257023226 | 0.447733943 | 0.34897998 | 0.888165931 |
| HMDB0002259 | Heptadecanoic acid | 276.9252384 | 380.994737 | 544.0057246 | 366.138205 | 219.6897546 | 382.0109327 | 400.6419 | 322.6129641 | 1.241865469 | 0.312508895 | 0.450150166 | 0.346642585 | 0.835378376 |
| HMDB0251322 | Dihydroouabain | 70.10459485 | 15.48954299 | 18.70932881 | 36.24077367 | 14.51134504 | 3.073138381 | 34.76782222 | 17.94175237 | 1.937816413 | 0.954431898 | 0.451506369 | 0.345336119 | 0.973326321 |
| HMDB0265702 | PA(22:1/20:4+=O(15)) | 0.666297288 | 0.799344912 | 0.836092561 | 0.631356417 | 2.542036859 | 0.816007481 | 0.76724492 | 1.329800252 | 0.576962532 | -0.79345046 | 0.452986592 | 0.343914653 | 0.842268564 |
| HMDB0031935 | Blumenol C O-[rhamnosyl-(1->6)-glucoside] | 307.1068849 | 8.138757236 | 6.319769213 | 364.742498 | 319.8662501 | 9.82484744 | 107.1884704 | 231.4778652 | 0.463061426 | -1.11072451 | 0.453375387 | 0.34354206 | 0.858530529 |
| HMDB0012553 | 12-Oxo-20-trihydroxy-leukotriene B4 | 26.84011507 | 6.497423244 | 8.626075808 | 12.26158838 | 105.595288 | 10.63363364 | 13.98787137 | 42.83017002 | 0.32658921 | -1.61445097 | 0.456622434 | 0.340442755 | 0.878317332 |
| HMDB0040709 | 10-Eicosene | 3.744828363 | 43.81773812 | 630.8410827 | 104.4884607 | 188.9781141 | 3089.313026 | 226.1345497 | 1127.5932 | 0.200546216 | -2.31799335 | 0.456717937 | 0.340351932 | 1.10371169 |
| HMDB0038334 | Dihydromelilotoside | 48.2986812 | 452.454377 | 485.0751696 | 316.3508787 | 47.57069596 | 225.2390321 | 328.6062065 | 196.3868702 | 1.673259552 | 0.74266125 | 0.457962093 | 0.339170469 | 0.449134404 |
| HMDB0251354 | Diisopropanolamine | 51.96226996 | 68.27824301 | 85.81761294 | 69.7229792 | 52.20107584 | 269.7078133 | 68.68604197 | 130.5439561 | 0.5261526 | -0.92644681 | 0.469483947 | 0.328379253 | 0.772593412 |
| HMDB0001190 | Indoleacetaldehyde | 75.5831576 | 339.8972589 | 201.6679116 | 233.6323684 | 103.0268895 | 60.44633419 | 205.7161094 | 132.3685307 | 1.554116437 | 0.636094597 | 0.47181056 | 0.326232343 | 0.763002745 |
| HMDB0303680 | Citrusin I | 2.641161761 | 6.456032309 | 8.372299664 | 1.293635542 | 5.456700432 | 60.90214223 | 5.823164578 | 22.55082607 | 0.258224003 | -1.95330498 | 0.476011984 | 0.322382113 | 0.344054173 |
| HMDB0266244 | PA(20:5-3OH(5,6,15)/22:6) | 36.29177235 | 1.441061903 | 2.203596513 | 19.98033369 | 81.63255344 | 1.463819184 | 13.31214359 | 34.35890211 | 0.3874438 | -1.36794104 | 0.476482237 | 0.321953285 | 0.690742288 |
| HMDB0029015 | Prolyl-Glutamine | 286.7703573 | 7.33240384 | 8.459403654 | 127.455302 | 729.713033 | 12.21272291 | 100.8540549 | 289.793686 | 0.348020194 | -1.52275707 | 0.477048395 | 0.321437561 | 0.851508209 |
| HMDB0302194 | Methylajoene | 682.3726507 | 470.3116198 | 472.0006855 | 805.8678404 | 801.1267913 | 384.4595454 | 541.561652 | 663.818059 | 0.815828441 | -0.29366229 | 0.478147515 | 0.320438097 | 0.636285251 |
| HMDB0304349 | ethylsulfenate | 838.2286812 | 1697.894635 | 728.6773486 | 1381.593035 | 251.718634 | 7399.565628 | 1088.266888 | 3010.959099 | 0.361435294 | -1.4681907 | 0.478178361 | 0.320410081 | 0.343649161 |
| HMDB0244231 | 1,5-Naphthalenediamine | 34.60276315 | 11.92763817 | 11.15040159 | 31.47710134 | 41.02598246 | 12.00353699 | 19.22693431 | 28.1688736 | 0.682559572 | -0.55097313 | 0.479992087 | 0.318765922 | 0.809200681 |
| HMDB0000220 | Palmitic acid | 16200.06523 | 19366.69232 | 22366.2109 | 16744.37749 | 10828.31231 | 21874.43824 | 19310.98948 | 16482.37602 | 1.171614424 | 0.22849786 | 0.48211463 | 0.316849689 | 0.868961997 |
| HMDB0036123 | [6]-Gingerdiol 5-O-betaglucopyranoside | 268.1867826 | 73.63218656 | 71.09586541 | 282.8887148 | 464.1620479 | 8.322147709 | 137.6382782 | 251.7909701 | 0.54663707 | -0.8713448 | 0.482765482 | 0.31626379 | 0.072272754 |
| HMDB0301945 | Hastatoside | 2.596746446 | 2.058750215 | 2.047509105 | 2.32913991 | 10.21227315 | 1.207514806 | 2.234335255 | 4.582975955 | 0.487529343 | -1.03643904 | 0.494462773 | 0.3058664 | 0.538414691 |
| HMDB0028691 | Alanylleucine | 296.7870873 | 211.5012857 | 242.9736356 | 376.4019494 | 835.5122995 | 49.65011009 | 250.4206695 | 420.521453 | 0.595500343 | -0.74782576 | 0.499359613 | 0.301586585 | 0.000161932 |
| HMDB0005085 | Leukotriene B4 dimethylamide | 10.12937821 | 14.05773535 | 6.983582076 | 1.437557473 | 8.927731201 | 87.01415652 | 10.39023188 | 32.45981507 | 0.320095227 | -1.64342693 | 0.504819556 | 0.296863829 | 0.051320483 |
| HMDB0011587 | MG(22:6/0:0/0:0) | 11.45353248 | 28.64092515 | 21.03015731 | 3.741582368 | 14.93319499 | 175.4893349 | 20.37487165 | 64.72137074 | 0.314809025 | -1.66745119 | 0.508282951 | 0.293894457 | 0.12013549 |
| HMDB0252084 | Ethylphenidate | 280.9419371 | 55.32806953 | 34.4380238 | 326.5384196 | 327.0928389 | 5.932293653 | 123.5693435 | 219.8545174 | 0.562050509 | -0.83122831 | 0.508941039 | 0.293332528 | 0.044214047 |
| HMDB0036820 | beta-Ionol | 37.38453906 | 38.35008515 | 56.34693669 | 31.94896741 | 19.76876496 | 54.54411415 | 44.02718697 | 35.4206155 | 1.242981985 | 0.313805387 | 0.509845527 | 0.292561386 | 0.902107354 |
| HMDB0251566 | Dodecanamide | 6808.490267 | 13716.63672 | 12359.90456 | 10140.85487 | 5681.391083 | 11231.01902 | 10961.67718 | 9017.754991 | 1.215566091 | 0.281628335 | 0.512930444 | 0.289941523 | 0.678887611 |
| HMDB0002350 | Octadecanol | 4303.566278 | 3424.789607 | 2958.669068 | 3039.830977 | 1175.021968 | 4346.265993 | 3562.341651 | 2853.706313 | 1.248321046 | 0.319989018 | 0.518096566 | 0.285589286 | 0.851998862 |
| HMDB0032476 | Polyoxyethylene (600) monoricinoleate | 10.83380695 | 17.11869697 | 39.07798414 | 18.65770136 | 8.069779509 | 20.49120576 | 22.34349602 | 15.73956221 | 1.419575445 | 0.505459524 | 0.52091269 | 0.283235062 | 0.657336578 |
| HMDB0060725 | 2,6-Pipecoloxylidide | 142.8868808 | 77.52824595 | 75.90443164 | 248.2812659 | 110.9509876 | 62.23034067 | 98.77318613 | 140.4875314 | 0.703074395 | -0.50825074 | 0.524601937 | 0.28017011 | 0.566687246 |
| HMDB0001257 | Spermidine | 1480.570794 | 1883.595776 | 2588.750788 | 2755.990111 | 1275.627864 | 275.599628 | 1984.305786 | 1435.739201 | 1.382079548 | 0.466840655 | 0.525621585 | 0.279326808 | 0.979522067 |
| HMDB0034496 | 6,10,14-Trimethyl-2-methylenepentadecanal | 116.4779391 | 59.80540708 | 76.38155885 | 67.19262177 | 26.26456758 | 102.3528405 | 84.22163501 | 65.27000995 | 1.290357318 | 0.367770624 | 0.531217986 | 0.274727229 | 0.818016904 |
| HMDB0000716 | Pipecolic acid | 898.7812231 | 755.1832376 | 687.7472491 | 1407.814423 | 2059.538927 | 73.6754041 | 780.5705699 | 1180.342918 | 0.661308301 | -0.59660508 | 0.533722306 | 0.272684646 | 0.281039031 |
| HMDB0003355 | 5-Aminopentanoic acid | 2245.552027 | 2561.807036 | 2290.537449 | 2279.733411 | 2792.587684 | 796.3130061 | 2365.965504 | 1956.211367 | 1.20946312 | 0.274366778 | 0.536427479 | 0.270488983 | 0.846600693 |
| HMDB0010396 | LysoPC(20:4/0:0) | 1369.818059 | 242.8875104 | 297.6926112 | 894.2300455 | 2651.237689 | 43.03140688 | 636.7993934 | 1196.16638 | 0.532366905 | -0.90950721 | 0.546900783 | 0.262091455 | 0.005938794 |
| HMDB0244120 | 1,2,3,4-Tetrahydronaphthalene | 232.5408973 | 228.9972762 | 327.4810634 | 259.1695894 | 123.1404424 | 288.3821869 | 263.0064123 | 223.5640729 | 1.176425214 | 0.234409611 | 0.548564219 | 0.260772524 | 0.768795064 |
| HMDB0011758 | Cer(d18:0/12:0) | 121.0979887 | 198.6629515 | 206.11276 | 195.9298952 | 72.38755069 | 167.0982951 | 175.2912334 | 145.1385803 | 1.207750779 | 0.272322783 | 0.549307779 | 0.260184251 | 0.738266454 |
| HMDB0028707 | Arginylglutamine | 24.18406236 | 47.12188903 | 30.54187535 | 50.05780975 | 91.84365102 | 8.672663396 | 33.94927558 | 50.19137472 | 0.676396607 | -0.56405867 | 0.550773511 | 0.259026955 | 0.057842462 |
| HMDB0039540 | Cerebronic acid | 185.9766564 | 212.6872034 | 306.8881982 | 276.3858522 | 125.2277832 | 192.8533119 | 235.1840193 | 198.1556491 | 1.186865075 | 0.247155936 | 0.551759669 | 0.258250048 | 0.779250007 |
| HMDB0011626 | Dodecanol | 4790.608918 | 4231.055181 | 4412.946443 | 3947.71682 | 1552.017213 | 5621.294819 | 4478.203514 | 3707.009617 | 1.208036659 | 0.272664236 | 0.552994829 | 0.257278929 | 0.837554979 |
| HMDB0000394 | 3-Hydroxytetradecanedioic acid | 27.08458807 | 58.1549752 | 51.79069351 | 50.76451281 | 15.89961408 | 42.93961682 | 45.67675226 | 36.53458124 | 1.25023336 | 0.322197404 | 0.554476305 | 0.256117008 | 0.700500842 |
| HMDB0060890 | Hydroxyterbinafine | 83.22892484 | 16.29249643 | 8.267131856 | 95.56221537 | 83.87443729 | 1.555639192 | 35.92951771 | 60.33076395 | 0.59554223 | -0.74772428 | 0.555172017 | 0.255572432 | 0.029619798 |
| HMDB0011177 | Phenylalanylproline | 502.655995 | 63.14212162 | 171.1012626 | 413.5488242 | 830.8823704 | 12.36354261 | 245.6331264 | 418.9315791 | 0.586332324 | -0.7702095 | 0.557000711 | 0.25414425 | 0.071287741 |
| HMDB0031336 | 9-Cycloheptadecen-1-one | 4.801587027 | 3.739308521 | 3.805006808 | 3.407770205 | 1.141446528 | 5.365423144 | 4.115300785 | 3.304879959 | 1.245219444 | 0.31640001 | 0.557473191 | 0.253776013 | 0.854859152 |
| HMDB0061797 | 2-Menthene | 78.87343464 | 83.30461175 | 94.9744858 | 86.90731896 | 44.25479784 | 94.60048861 | 85.71751073 | 75.2542018 | 1.139039531 | 0.187817818 | 0.557654827 | 0.253634534 | 0.76203019 |
| HMDB0012642 | 20-Oxo-leukotriene E4 | 1332.720243 | 124.0727163 | 183.8729595 | 1243.935387 | 1479.396091 | 44.43015606 | 546.8886395 | 922.5872113 | 0.592777174 | -0.7544382 | 0.561022976 | 0.251019352 | 0.26792915 |
| HMDB0003555 | Vitamin K1 | 6.466226043 | 8.053199262 | 27.27906418 | 10.53987495 | 1.928276949 | 14.7547747 | 13.93282983 | 9.074308864 | 1.535414987 | 0.618628636 | 0.561356294 | 0.250761404 | 0.721319141 |
| HMDB0001984 | Finasteride | 237.7680534 | 59.1321846 | 61.17976389 | 113.973867 | 107.1556298 | 7.267873458 | 119.3600006 | 76.13245674 | 1.567793892 | 0.64873591 | 0.562370063 | 0.249977806 | 0.774545923 |
| HMDB0032142 | 2,4-Dimethylbenzaldehyde | 348.5216574 | 14.62268643 | 31.92518254 | 14.46658898 | 103.7045538 | 52.79464788 | 131.6898421 | 56.98859687 | 2.310810396 | 1.20839889 | 0.565882569 | 0.247273683 | 0.222745047 |
| HMDB0004610 | Phytosphingosine | 6177.555939 | 8055.542988 | 8827.734699 | 7156.991161 | 4008.367408 | 8863.252814 | 7686.944542 | 6676.203794 | 1.151394532 | 0.203382266 | 0.567684164 | 0.24589322 | 0.73854076 |
| HMDB0000638 | Dodecanoic acid | 1981.06863 | 2112.795599 | 2573.269531 | 1981.924809 | 969.4386659 | 2718.750789 | 2222.37792 | 1890.038088 | 1.175837637 | 0.233688863 | 0.570122763 | 0.244031619 | 0.79466204 |
| HMDB0033210 | Lyciumoside VIII | 1052.941107 | 329.6862851 | 220.9994032 | 1338.966488 | 1121.752899 | 33.02668587 | 534.542265 | 831.2486909 | 0.643059377 | -0.63697614 | 0.570733567 | 0.243566585 | 0.124471228 |

## *Supplementary Table 1*
## Positive Mode

| HMDB_ID | Compound_name | DB]a,l]P-#1 | DB]a,l]P-#2 | DB]a,l]P-#3 | DMSO-#1 | DMSO-#2 | DMSO-#3 | AVE (DB[a,l]P) | AVE (DMSO) | FC (DB[a,l]P/ DMSO) | Log2 (FC) | T-Test | Log10 (P_value) | VIP |
|---|---|---|---|---|---|---|---|---|---|---|---|---|---|---|
| HMDB0034933 | 11-Eicosen-1-ol | 889.6262059 | 732.0052532 | 564.6512026 | 522.6029426 | 289.0992653 | 969.4783303 | 728.7608872 | 593.7268461 | 1.227434623 | 0.295646184 | 0.573467001 | 0.241491568 | 0.822750586 |
| HMDB0000251 | Taurine | 109.1159807 | 130.0812868 | 91.72028665 | 148.9843745 | 163.4283651 | 73.14172641 | 110.3058514 | 128.5181553 | 0.85829003 | -0.22046285 | 0.577987601 | 0.238081478 | 0.416774458 |
| HMDB0038213 | Humulol | 33.33520158 | 38.49217065 | 37.90367528 | 24.38606039 | 16.09384424 | 50.32325823 | 36.57701584 | 30.26772095 | 1.208449618 | 0.273157327 | 0.578136234 | 0.237969811 | 0.897872453 |
| HMDB0258433 | Spisulosine | 103.9025275 | 122.2160766 | 131.1445267 | 120.9335832 | 60.60432015 | 134.2894771 | 119.0877103 | 105.2757935 | 1.131197461 | 0.177850787 | 0.596406272 | 0.224457798 | 0.713202189 |
| HMDB0032472 | Polyethylene, oxidized | 33.49078822 | 70.32884207 | 50.14021123 | 54.34116209 | 19.74208724 | 53.14272979 | 51.31994717 | 42.40865971 | 1.210128958 | 0.275160797 | 0.597414444 | 0.223724281 | 0.648442348 |
| HMDB0014581 | Allopurinol | 496.1683303 | 632.2171295 | 309.4543337 | 678.3076553 | 379.0956824 | 17.43885785 | 479.2799312 | 358.2807318 | 1.33772176 | 0.419778073 | 0.599921277 | 0.221905735 | 0.904860208 |
| HMDB0037519 | 2-(5,8-Tetradecadienyl)cyclobutanone | 3262.715603 | 3673.2736 | 4405.755552 | 3929.429422 | 1916.270041 | 4181.322366 | 3780.581585 | 3342.34061 | 1.131117988 | 0.177749426 | 0.609008425 | 0.2153767 | 0.691708475 |
| HMDB0011563 | MG(15:0/0:0/0:0) | 195.8474994 | 228.0166256 | 212.3699707 | 201.4381252 | 99.8320685 | 257.152601 | 212.0780319 | 186.1409316 | 1.139341198 | 0.188199854 | 0.610291561 | 0.214462635 | 0.730636478 |
| HMDB0032995 | 2-Undecyl-4(1H)-quinolinone N-oxide | 32.45671818 | 37.85658603 | 55.4954398 | 41.07589395 | 18.65763542 | 47.79053986 | 41.93624801 | 35.84135641 | 1.170051923 | 0.226572554 | 0.615946157 | 0.21045725 | 0.671719944 |
| HMDB0041944 | N-Acetylprocainamide | 9.412716878 | 22.780165 | 11.84910722 | 15.1675145 | 16.26435165 | 2.966866531 | 14.68066304 | 11.46624423 | 1.280337549 | 0.356524214 | 0.616029694 | 0.210398353 | 0.716533239 |
| HMDB0061864 | Dihomolinoleic acid | 35.02947146 | 13.61312543 | 15.65503024 | 12.05889341 | 11.08025 | 27.22170295 | 21.43254237 | 16.78694879 | 1.276738414 | 0.352462967 | 0.617527506 | 0.209343693 | 0.60021522 |
| HMDB0304805 | Leu-Pro-Ile | 97.71084664 | 51.91043365 | 90.07615587 | 122.0806933 | 45.94777428 | 18.46177291 | 79.89914539 | 62.16341349 | 1.285308205 | 0.362114345 | 0.630213296 | 0.200512438 | 0.88201505 |
| HMDB0252688 | Gentamicin C | 30.70538145 | 3.407163871 | 6.343045327 | 22.55567367 | 39.00793023 | 0.800279463 | 13.48519688 | 20.78796112 | 0.648702237 | -0.62437168 | 0.630564197 | 0.200270692 | 0.012482353 |
| HMDB0000927 | Valerylglycine | 2923.412292 | 2228.544946 | 2133.460549 | 3359.878714 | 2179.740249 | 386.7864149 | 2428.472596 | 1975.468459 | 1.229314791 | 0.297854394 | 0.640987276 | 0.193150591 | 0.825816825 |
| HMDB0000806 | Myristic acid | 2116.682866 | 2393.835914 | 2248.076671 | 2141.107925 | 1061.207118 | 2793.775504 | 2252.86515 | 1998.696849 | 1.127167009 | 0.172701292 | 0.645321321 | 0.190223986 | 0.697712911 |
| HMDB0255868 | O-Succinylhomoserine | 18.3599959 | 25.95805693 | 24.10823162 | 46.60116728 | 4.638858175 | 36.22275869 | 22.80876149 | 29.15426138 | 0.782347431 | -0.35411866 | 0.6466824 | 0.189308959 | 0.189192075 |
| HMDB0302269 | Pentenylbenzene | 62.38731584 | 74.12017939 | 83.26297914 | 76.47479736 | 38.4776249 | 82.54206105 | 73.25682479 | 65.83149443 | 1.112792979 | 0.154185222 | 0.647695514 | 0.188629111 | 0.640372226 |
| HMDB0294958 | DG(22:6-2OH/13:0/0:0) | 0.489892366 | 1.51858238 | 18.34995638 | 0.434232084 | 41.02117078 | 0.59487274 | 6.786143707 | 14.01675854 | 0.48414501 | -1.04648887 | 0.648385355 | 0.188166803 | 0.077983997 |
| HMDB0013219 | Behenoylglycine | 21.95335017 | 27.01290061 | 27.3812716 | 25.43996939 | 12.69874859 | 30.11226586 | 25.44917413 | 22.75032795 | 1.118628891 | 0.161731497 | 0.64878374 | 0.187900043 | 0.660220384 |
| HMDB0304804 | Leu-Leu-Tyr | 12.84485059 | 45.28525007 | 47.83304404 | 11.43838044 | 35.00745841 | 103.4552246 | 35.32104823 | 49.96702115 | 0.706887211 | -0.50044805 | 0.648897822 | 0.187823684 | 0.185843689 |
| HMDB0003119 | Benzyl alcohol | 339.8920699 | 83.76742752 | 51.47369013 | 336.5064551 | 255.0041536 | 63.28821692 | 158.3777292 | 218.2662752 | 0.725617043 | -0.46271975 | 0.649194699 | 0.187625035 | 0.621971128 |
| HMDB0028879 | Histidylarginine | 62.994162 | 367.2511805 | 255.7183851 | 179.5445735 | 260.156983 | 98.75353347 | 228.6545759 | 179.48503 | 1.273947894 | 0.349306271 | 0.649779492 | 0.187234 | 0.171517801 |
| HMDB0059875 | Carvotanacetone | 79.13411264 | 85.58153034 | 115.3112753 | 96.20459787 | 53.24384526 | 102.555931 | 93.34230609 | 84.00145803 | 1.111198642 | 0.152116741 | 0.650054309 | 0.187050359 | 0.605626228 |
| HMDB0040449 | 2-thoxymethyl)phenol | 179.8039261 | 13.99582066 | 47.18789182 | 54.86657744 | 32.84377095 | 78.77502709 | 80.32921286 | 55.49512516 | 1.447500346 | 0.533563693 | 0.660018561 | 0.180443851 | 0.093706994 |
| HMDB0061659 | 2-hydroxyicosanoic acid | 485.5567933 | 598.1134915 | 722.3132413 | 781.5652093 | 168.1376278 | 581.609892 | 601.9945087 | 510.4375764 | 1.179369499 | 0.238015789 | 0.660168719 | 0.180345058 | 0.732517686 |
| HMDB0030935 | 9,10,13-Trihydroxystearic acid | 16.66400131 | 9.951530497 | 7.022513111 | 21.5510351 | 4.030711754 | 16.50009879 | 11.21268164 | 14.02728188 | 0.799348137 | -0.32310412 | 0.660185587 | 0.180333961 | 0.151120004 |
| HMDB0094648 | Leu-Leu-Leu | 88.73474165 | 25.38677936 | 41.04114994 | 82.96244513 | 25.99624631 | 3.427006577 | 51.72089032 | 37.46189934 | 1.380626483 | 0.465323063 | 0.663258518 | 0.178317164 | 0.86723592 |
| HMDB0244121 | Tetrahydropyridine | 697.3483666 | 673.1246797 | 568.2992347 | 1759.254812 | 764.4566363 | 93.54977608 | 646.257427 | 872.420408 | 0.740763766 | -0.43291456 | 0.665559448 | 0.176813147 | 0.322875112 |
| HMDB0000734 | Indoleacrylic acid | 1826.664444 | 731.894395 | 570.0212116 | 1409.551453 | 2175.985922 | 432.5317441 | 1042.860017 | 1339.356373 | 0.778627733 | -0.36099436 | 0.667513861 | 0.175539712 | 0.346627944 |
| HMDB0031923 | 10,20-Dihydroxyeicosanoic acid | 1166.468928 | 1600.928375 | 1705.431743 | 1405.498095 | 786.2991523 | 1809.813419 | 1490.943016 | 1333.870222 | 1.117757178 | 0.160606811 | 0.668384028 | 0.174973937 | 0.609796444 |
| HMDB0072871 | MG(21:0/0:0/0:0) | 125.4408399 | 152.5215519 | 196.7512357 | 157.6642828 | 89.95703636 | 180.9285818 | 158.2378758 | 142.849967 | 1.107720773 | 0.147594262 | 0.676925906 | 0.169458865 | 0.560839521 |
| HMDB0061664 | 12-hydroxyicosanoic acid | 2148.131775 | 2120.074549 | 2350.125232 | 2221.093996 | 1142.401334 | 2648.521947 | 2206.110519 | 2004.005759 | 1.100850389 | 0.138618412 | 0.679193744 | 0.168006323 | 0.635913218 |
| HMDB0257422 | S-oxocysteine | 370.8183701 | 394.9505689 | 422.5352682 | 358.5980639 | 204.0518052 | 507.3541945 | 396.1014024 | 356.6680212 | 1.110560462 | 0.151287939 | 0.680046458 | 0.167461417 | 0.668038918 |
| HMDB0011111 | Malonic semialdehyde | 210.5934915 | 298.9522766 | 164.6831009 | 290.1686861 | 150.0516342 | 321.559083 | 224.7429563 | 253.9264678 | 0.885071014 | -0.17613488 | 0.680405142 | 0.167232413 | 0.393042866 |
| HMDB0000015 | Cortexolone | 7.203596495 | 40.17828885 | 55.64158586 | 28.67735328 | 13.27018357 | 101.5252901 | 34.34115707 | 47.82427566 | 0.71806957 | -0.47780447 | 0.683560641 | 0.165222951 | 0.354850066 |
| HMDB0000562 | Creatinine | 62.15149879 | 71.72237885 | 56.00390224 | 97.46332516 | 67.96372178 | 45.2237899 | 63.29259329 | 70.21694561 | 0.901386307 | -0.14978256 | 0.683809047 | 0.165065158 | 0.285441495 |
| HMDB0302880 | N-Isobutyloctadeca-2-4-dienamide | 2739.688338 | 2218.920106 | 1953.310403 | 1814.004746 | 964.9285883 | 3240.957354 | 2303.972949 | 2006.63023 | 1.148180126 | 0.199348989 | 0.69410005 | 0.158577924 | 0.694305129 |
| HMDB0259938 | Xylazine | 9.256413635 | 11.32676841 | 19.80856104 | 10.22036369 | 5.11174734 | 18.61391058 | 13.46391436 | 11.31534054 | 1.189881499 | 0.250817902 | 0.694666554 | 0.158223611 | 0.627515658 |
| HMDB0012273 | Palmitic amide | 55130.07888 | 57607.71878 | 48867.51206 | 49144.00931 | 27854.64935 | 69530.0337 | 53868.43657 | 48842.89745 | 1.102891912 | 0.141291408 | 0.70398791 | 0.152434799 | 0.624030925 |
| HMDB0033469 | N1,N10-Dicoumaroylspermidine | 28.45055689 | 52.8060747 | 32.42907516 | 18.11220652 | 50.08442233 | 31.14158488 | 37.89523558 | 33.11273791 | 1.144430753 | 0.194630171 | 0.709699124 | 0.148925731 | 0.554956077 |
| HMDB0246585 | 4-Tetradecanamidobenzylphosphonic acid | 414.5453017 | 553.7442171 | 843.5288945 | 565.9913094 | 299.5788978 | 733.6533106 | 603.9394711 | 533.0745059 | 1.132936324 | 0.180066777 | 0.711957885 | 0.147545695 | 0.490497521 |
| HMDB0036156 | Deoxynivalenol | 20.34741806 | 72.0512528 | 65.09862936 | 52.1972053 | 16.6380144 | 63.19972104 | 52.49910007 | 44.01164691 | 1.192845616 | 0.254407334 | 0.712468116 | 0.147234566 | 0.357278099 |
| HMDB0034301 | Piperidine | 9979.485459 | 2907.300772 | 1979.908831 | 7823.928203 | 9460.493582 | 1487.967296 | 4955.565021 | 6257.463027 | 0.791944755 | -0.3365283 | 0.729218372 | 0.137142398 | 0.322111119 |
| HMDB0254679 | Metomidate | 4.953825122 | 9.511054885 | 2.924765028 | 9.904509227 | 2.844880581 | 0.915493643 | 5.796548345 | 4.55496115 | 1.272579096 | 0.347755329 | 0.730103696 | 0.136615453 | 0.755243547 |
| HMDB0000826 | Pentadecanoic acid | 40.6375657 | 37.25002206 | 38.05994113 | 39.36314655 | 17.63211022 | 49.24081699 | 38.6491763 | 35.41202458 | 1.091413912 | 0.126198339 | 0.747694934 | 0.126275562 | 0.586055992 |
| HMDB0012639 | 20-Hydroxy-leukotriene E4 | 195.2527784 | 20.83309987 | 11.47790737 | 85.76078428 | 71.50204399 | 3.136713079 | 75.85459523 | 53.46651378 | 1.4187309 | 0.50460097 | 0.747768027 | 0.126233108 | 0.227725173 |
| HMDB0031008 | 15,16-Dihydroxyoctadecanoic acid | 64.70523029 | 54.58107743 | 46.45181286 | 71.50342166 | 16.25389916 | 59.91791797 | 55.24604019 | 49.2250796 | 1.122314898 | 0.166477523 | 0.749908047 | 0.124991986 | 0.625632573 |
| HMDB0029069 | Threonylproline | 147.8659306 | 99.19476527 | 63.68600442 | 297.8775136 | 93.45282262 | 10.40484346 | 103.5822334 | 133.9117266 | 0.773511298 | -0.37050573 | 0.749967588 | 0.124957506 | 0.421836453 |

## *Supplementary Table 1*
## Positive Mode

| HMDB_ID | Compound_name | DB]a,l]P-#1 | DB]a,l]P-#2 | DB]a,l]P-#3 | DMSO-#1 | DMSO-#2 | DMSO-#3 | AVE (DB[a,l]P) | AVE (DMSO) | FC (DB[a,l]P/ DMSO) | Log2 (FC) | T-Test | Log10 (P_value) | VIP |
|---|---|---|---|---|---|---|---|---|---|---|---|---|---|---|
| HMDB0028813 | Glutamylarginine | 331.1319856 | 433.8877607 | 278.6524986 | 619.1899877 | 211.7088608 | 31.34055939 | 347.8907483 | 287.413136 | 1.210420488 | 0.275508312 | 0.753432922 | 0.122955407 | 0.883489086 |
| HMDB0000086 | Glycerophosphocholine | 465.6865699 | 375.8575012 | 372.6602521 | 492.4628392 | 455.0508964 | 156.0159574 | 404.7347744 | 367.843231 | 1.100291484 | 0.137885766 | 0.755766844 | 0.121612165 | 0.595194188 |
| HMDB0033608 | 2-Heptadecylfuran | 11.51710922 | 5.886981528 | 5.168121008 | 5.930579995 | 3.706320912 | 10.23318809 | 7.524070584 | 6.623362999 | 1.135989464 | 0.183949454 | 0.761742775 | 0.118191656 | 0.393413527 |
| HMDB0003040 | Arabinosylhypoxanthine | 102.0082417 | 67.7098854 | 65.12636564 | 144.4182424 | 128.0859568 | 5.684602538 | 78.28149758 | 92.72960057 | 0.844191036 | -0.24435858 | 0.766038873 | 0.115749191 | 0.498609153 |
| HMDB0029632 | Oleyl alcohol | 53.62412073 | 59.65290776 | 124.5907042 | 76.51857018 | 44.76785655 | 91.50416367 | 79.28924423 | 70.9301968 | 1.117848925 | 0.160725224 | 0.768780908 | 0.11419741 | 0.283190808 |
| HMDB0041107 | 5-Eicosyne | 117.3932724 | 98.98814605 | 106.3634729 | 99.13640103 | 38.7836321 | 153.251 | 107.5816305 | 97.05701104 | 1.108437498 | 0.148527422 | 0.76903073 | 0.114056306 | 0.632962855 |
| HMDB0246022 | 3,4-Diaminopyridine | 46.89955375 | 74.28111567 | 40.33292801 | 50.00966882 | 72.26886327 | 22.78681639 | 53.83786581 | 48.35511616 | 1.113385099 | 0.154952681 | 0.772038255 | 0.112361179 | 0.497025679 |
| HMDB0013279 | N-Nonanoylglycine | 191.6843308 | 122.6962024 | 96.18198212 | 262.5545091 | 40.25635793 | 33.06905422 | 136.8541718 | 111.9599738 | 1.222349088 | 0.28965636 | 0.772640098 | 0.112022757 | 0.909274463 |
| HMDB0248559 | Arachidoyl Ethanolamide | 919.3457665 | 1132.748485 | 1401.809154 | 1265.9462 | 630.8260948 | 1317.159673 | 1151.301135 | 1071.310656 | 1.074665998 | 0.103888345 | 0.774671799 | 0.110882254 | 0.453174005 |
| HMDB0028934 | Leucyl-Lysine | 2111.861557 | 1741.271683 | 1496.191988 | 3848.287081 | 2315.686846 | 175.4099159 | 1783.108409 | 2113.128188 | 0.843824061 | -0.24498587 | 0.775176087 | 0.110599633 | 0.480895097 |
| HMDB0000267 | Pyroglutamic acid | 125.3355364 | 74.24669427 | 37.51851131 | 127.7932577 | 53.74655597 | 17.95372373 | 79.03358064 | 66.4978458 | 1.188513398 | 0.249158166 | 0.775906104 | 0.110190831 | 0.549057121 |
| HMDB0004207 | Glutamyllysine | 185.7935209 | 97.20607467 | 66.89331636 | 296.8189426 | 123.0038438 | 11.82206067 | 116.6309707 | 143.8816157 | 0.810603704 | -0.30293133 | 0.777806212 | 0.109128593 | 0.373767616 |
| HMDB0034297 | Ricinoleic acid | 8.801495077 | 7.845652911 | 5.179948024 | 12.0090961 | 4.950607606 | 6.980289194 | 7.275698671 | 7.979997634 | 0.911741958 | -0.13330252 | 0.780353768 | 0.107708468 | 0.198913307 |
| HMDB0060021 | isoeugenol-O-glucuronide | 13.05292428 | 67.77438366 | 25.14866015 | 46.57944216 | 14.98860397 | 63.55375024 | 35.3253227 | 41.70726545 | 0.84698247 | -0.23959599 | 0.784854301 | 0.105210958 | 0.391554026 |
| HMDB0031039 | Heptadecanal | 23.15522269 | 17.81230389 | 35.9949325 | 27.44079802 | 15.93370621 | 27.77966214 | 25.65415303 | 23.71805545 | 1.081629692 | 0.113206662 | 0.785527414 | 0.104838654 | 0.288594318 |
| HMDB0038691 | Kiwiionoside | 2493.640018 | 1374.083833 | 707.772102 | 2504.298032 | 1099.182835 | 243.1598483 | 1525.165318 | 1282.213572 | 1.189478377 | 0.250329045 | 0.786824829 | 0.104121944 | 0.586194789 |
| HMDB0302963 | 22-Hydroxydocosanoic acid | 656.8274397 | 774.4008499 | 1045.145441 | 841.1419159 | 523.6994612 | 967.3623398 | 825.4579101 | 777.4012389 | 1.061817076 | 0.086535247 | 0.797252296 | 0.098404221 | 0.366591694 |
| HMDB0031445 | Undecane | 43.0421576 | 48.97753591 | 68.81594627 | 57.26646082 | 28.88664036 | 63.79311647 | 53.61187993 | 49.98207255 | 1.072622186 | 0.101142 | 0.797671766 | 0.09817578 | 0.418688325 |
| HMDB0003352 | Menthol | 18.18665164 | 15.69680844 | 12.71985183 | 15.22717072 | 8.993836775 | 19.60637407 | 15.5344373 | 14.60912719 | 1.06333781 | 0.088599997 | 0.802412303 | 0.095602421 | 0.411942533 |
| HMDB0039947 | Pisumionoside | 59.68611896 | 128.3613027 | 115.8672326 | 92.42627175 | 139.5849131 | 44.19018051 | 101.3048847 | 92.06712177 | 1.100337263 | 0.13794579 | 0.803256716 | 0.095145634 | 0.410165713 |
| HMDB0072839 | MG(19:0/0:0/0:0) | 368.9252227 | 420.977143 | 439.5374737 | 414.2808826 | 217.9813432 | 524.9512936 | 409.8132798 | 385.7378398 | 1.062413996 | 0.087346058 | 0.80691423 | 0.093172626 | 0.489068756 |
| HMDB0029349 | Neryl rhamnosyl-glucoside | 297.8416771 | 174.7618755 | 213.5965542 | 398.4331642 | 166.176409 | 38.37451779 | 228.7333689 | 200.994697 | 1.138006984 | 0.186509412 | 0.81574108 | 0.088447666 | 0.743547262 |
| HMDB0029074 | Threonylvaline | 38.42870197 | 53.2421797 | 40.00712278 | 84.0830489 | 23.92326486 | 5.763456073 | 43.89266815 | 37.92325661 | 1.157407672 | 0.210897112 | 0.816784081 | 0.087892735 | 0.862633138 |
| HMDB0000162 | Proline | 644.4940265 | 512.5834721 | 383.1324146 | 548.571146 | 626.3129268 | 267.5334234 | 513.4033044 | 480.8058321 | 1.067797581 | 0.094638186 | 0.817833601 | 0.08733505 | 0.375415756 |
| HMDB0033529 | 2,3-Dihydro-3-methyl-1H-pyrrole | 675.8086178 | 957.5826218 | 835.9669737 | 1448.001537 | 634.4664874 | 104.8333958 | 823.1194045 | 729.1004734 | 1.128951954 | 0.17498409 | 0.825307619 | 0.083384145 | 0.777978466 |
| HMDB0032860 | 1-Methylnaphthalene | 73.18634711 | 89.24286265 | 96.48897322 | 93.12633379 | 28.5102649 | 118.3865282 | 86.30606099 | 80.00770897 | 1.078721815 | 0.109322864 | 0.830878216 | 0.080462627 | 0.554955058 |
| HMDB0303271 | Polyglycerol esters of fatty acids | 11.58882696 | 11.48095471 | 23.38737822 | 17.62375187 | 6.608592552 | 27.01579612 | 15.48571997 | 17.08271351 | 0.906514059 | -0.1415987 | 0.833022118 | 0.079343467 | 0.002676314 |
| HMDB0029007 | Phenylalanyltyrosine | 16.98412282 | 50.86660596 | 51.71171318 | 34.67823532 | 64.21710611 | 7.935018758 | 39.85414732 | 35.61012006 | 1.119180369 | 0.162442563 | 0.841350077 | 0.075023261 | 0.486334893 |
| HMDB0255618 | Nipecotic acid | 996.6967611 | 852.2486618 | 948.2840016 | 1707.815308 | 1177.095217 | 194.4032279 | 932.4098082 | 1026.437917 | 0.908393769 | -0.13861029 | 0.843106715 | 0.074117452 | 0.403120876 |
| HMDB0029022 | Prolyl-Lysine | 102.9600659 | 3.205679288 | 4.667849335 | 81.580873 | 50.34027255 | 4.141474417 | 36.94453152 | 45.35420666 | 0.814577836 | -0.29587553 | 0.843544752 | 0.073891872 | 0.603838355 |
| HMDB0002109 | Hydroxysepiapterin | 330.2304306 | 404.8876627 | 426.3722187 | 293.2451064 | 120.441227 | 652.3676406 | 387.1634373 | 355.3513246 | 1.089522989 | 0.123696638 | 0.851501318 | 0.069814676 | 0.643527176 |
| HMDB0011759 | Cer(d18:0/14:0) | 317.4011577 | 405.6023506 | 187.4069225 | 382.78198 | 181.5372953 | 401.9877784 | 303.4701436 | 322.1023513 | 0.942154388 | -0.08596461 | 0.853761967 | 0.068663196 | 0.184823891 |
| HMDB0028923 | Leucyl-Arginine | 120.5191452 | 193.3497117 | 142.3051998 | 147.4960556 | 279.6505716 | 64.62760679 | 152.0580189 | 163.9247446 | 0.927608698 | -0.10841175 | 0.866506985 | 0.062227932 | 0.190018398 |
| HMDB0000391 | 7-Ketodeoxycholic acid | 4.305813422 | 24.12401767 | 60.76286544 | 18.83671041 | 33.45212806 | 27.89371274 | 29.73089884 | 26.72751707 | 1.1123704 | 0.15363726 | 0.868936864 | 0.061011778 | 0.435886477 |
| HMDB0112193 | 6-Hydroxyoctadecanoic acid | 1943.950896 | 1497.093144 | 1181.283353 | 2170.097789 | 546.5352045 | 1632.406246 | 1540.775798 | 1449.679747 | 1.062838742 | 0.087922722 | 0.870980655 | 0.059991491 | 0.452866357 |
| HMDB0002212 | Arachidic acid | 1515.237275 | 1666.487967 | 2852.19755 | 2106.715426 | 1247.890055 | 2399.227538 | 2011.307597 | 1917.94434 | 1.048678815 | 0.068572883 | 0.872515901 | 0.05922665 | 0.195851187 |
| HMDB0000721 | Glycylproline | 56.08001067 | 102.9365312 | 55.41745971 | 89.97575465 | 126.2983924 | 16.57621848 | 71.47800051 | 77.6167885 | 0.920909018 | -0.11886946 | 0.872530985 | 0.059219142 | 0.300302326 |
| HMDB0037798 | 10,16-Dihydroxyhexadecanoic acid | 85.65697321 | 77.49697593 | 64.9897123 | 109.8670577 | 21.29041167 | 113.2091542 | 76.04788715 | 81.4555412 | 0.933612201 | -0.09910468 | 0.875300645 | 0.057842751 | 0.309040149 |
| HMDB0001406 | Niacinamide | 640.6439483 | 1927.646896 | 1038.463744 | 1205.776508 | 669.576877 | 2003.813957 | 1202.251529 | 1293.055781 | 0.929775457 | -0.10504575 | 0.875334904 | 0.057825754 | 0.195544827 |
| HMDB0244507 | 13-Docosenamide | 2458.992414 | 8472.889045 | 10556.33233 | 8667.888341 | 3843.370606 | 10550.88431 | 7162.737929 | 7687.381086 | 0.93175268 | -0.10198103 | 0.875554127 | 0.057717001 | 0.294411515 |
| HMDB0033122 | Glycosminine | 23.8681937 | 20.45268097 | 33.91028791 | 35.15463203 | 9.807763658 | 38.20218423 | 26.07705419 | 27.72152664 | 0.940678864 | -0.08822581 | 0.87569119 | 0.05764902 | 0.186480103 |
| HMDB0059850 | Ethyl 3-hydroxydodecanoate | 959.370613 | 1255.409447 | 1227.28122 | 1370.190634 | 550.660876 | 1686.025836 | 1147.35376 | 1202.292449 | 0.954305054 | -0.06747758 | 0.883283673 | 0.053899797 | 0.154260279 |
| HMDB0031078 | Pentadecanal | 38.9981571 | 44.71247727 | 39.53092388 | 40.70504741 | 27.25513943 | 52.00224815 | 41.08051942 | 39.98747833 | 1.027334584 | 0.038906117 | 0.889444153 | 0.050881315 | 0.325352386 |
| HMDB0010386 | LysoPC(18:2/0:0) | 3.904302946 | 9.875114455 | 14.9055683 | 9.139287529 | 9.430176891 | 8.751536292 | 9.561661901 | 9.107000237 | 1.049924416 | 0.070285471 | 0.893413043 | 0.048947711 | 0.219892985 |
| HMDB0013302 | Phenylalanylphenylalanine | 269.7711131 | 303.5575295 | 510.1100734 | 576.44522 | 389.4962719 | 175.0660053 | 361.1462387 | 380.3358324 | 0.949545659 | -0.07469072 | 0.896248534 | 0.047571542 | 0.084503651 |
| HMDB0006462 | Homocysteinesulfinic acid | 61.30461248 | 130.4264514 | 55.48602951 | 83.15890956 | 35.97315983 | 114.2887511 | 82.40569779 | 77.80694017 | 1.059104723 | 0.082845248 | 0.896308444 | 0.047542512 | 0.201472274 |
| HMDB0011760 | Cer(d18:0/16:0) | 136.1783542 | 212.2770275 | 216.0557134 | 245.5479025 | 92.90966527 | 204.4635791 | 188.1703651 | 180.9737156 | 1.039766269 | 0.056259258 | 0.897595116 | 0.046919519 | 0.327292025 |
| HMDB0061714 | Docosadienoate (22:2n6) | 53.09984878 | 53.59616027 | 76.45514138 | 67.6092171 | 32.22330951 | 76.95233685 | 61.05038348 | 58.92828782 | 1.036011494 | 0.051040008 | 0.898687375 | 0.04639136 | 0.33061638 |
| HMDB0010382 | LysoPC(16:0/0:0) | 20.14246991 | 25.72617051 | 94.80567906 | 22.10280675 | 81.10379741 | 25.46287852 | 46.89143983 | 42.88982756 | 1.093299798 | 0.128689063 | 0.902588796 | 0.044510062 | 0.075123966 |
| HMDB0001713 | m-Coumaric acid | 338.00049 | 78.96073635 | 60.33115417 | 259.1105634 | 183.2733085 | 75.32393839 | 159.0974602 | 172.5692701 | 0.9219339 | -0.11726478 | 0.903442113 | 0.044099669 | 0.46139187 |
| HMDB0000292 | Xanthine | 88.78123139 | 139.8423578 | 53.28066778 | 169.6388814 | 86.06073576 | 5.583359576 | 93.96808564 | 87.09432559 | 1.078923168 | 0.109592131 | 0.904168223 | 0.04375076 | 0.685349487 |
| HMDB0036187 | 2-[(2-Furanylmethyl)thio]-6-methylpyrazine | 10492.87312 | 12786.17727 | 13801.59886 | 13767.81015 | 4010.614792 | 17712.63398 | 12360.21642 | 11830.35297 | 1.044788473 | 0.063210885 | 0.905431426 | 0.043144436 | 0.492064404 |
| HMDB0000177 | Histidine | 90.83637261 | 51.73308511 | 38.30915645 | 70.98637348 | 87.10961905 | 30.658697 | 60.29287139 | 62.91822984 | 0.958273485 | -0.06149064 | 0.91470829 | 0.038717385 | 0.058102015 |
| HMDB0000660 | Fructose | 1411.242321 | 1645.973339 | 1196.105294 | 1624.087474 | 1879.996293 | 625.9259885 | 1417.773651 | 1376.669919 | 1.029857362 | 0.042444535 | 0.923862631 | 0.034392599 | 0.385240754 |

## *Supplementary Table 1*
**Positive Mode**

| HMDB_ID | Compound_name | DB]a,l]P-#1 | DB]a,l]P-#2 | DB]a,l]P-#3 | DMSO-#1 | DMSO-#2 | DMSO-#3 | AVE (DB[a,l]P) | AVE (DMSO) | FC (DB[a,l]P/ DMSO) | Log2 (FC) | T-Test | Log10 (P_value) | VIP |
|---|---|---|---|---|---|---|---|---|---|---|---|---|---|---|
| HMDB0248328 | Aminopentol | 139.4196651 | 117.1466425 | 87.57577334 | 194.5115534 | 35.61181473 | 128.3145014 | 114.714027 | 119.4792898 | 0.960116411 | -0.05871876 | 0.926408651 | 0.033197398 | 0.316737419 |
| HMDB0011619 | Arachidyl alcohol | 202.4569521 | 584.2676409 | 704.9123147 | 517.0821558 | 246.6973363 | 671.5483768 | 497.2123026 | 478.442623 | 1.039230785 | 0.055516073 | 0.928250753 | 0.03233469 | 0.00346297 |
| HMDB0003157 | Guanidinosuccinic acid | 113.4907169 | 29.64233201 | 19.58616696 | 79.93569473 | 74.12423498 | 16.6328179 | 54.23973864 | 56.89758254 | 0.953287226 | -0.06901713 | 0.944653192 | 0.024727604 | 0.214601829 |
| HMDB0031502 | 4-Hexen-1-ol | 1192.367169 | 2335.080341 | 1935.056718 | 1447.444939 | 1039.822824 | 3132.393061 | 1820.834743 | 1873.220275 | 0.972034505 | -0.04092057 | 0.945695437 | 0.024248707 | 0.126671543 |
| HMDB0060484 | Indolepyruvate | 13.18124939 | 53.55984177 | 52.73527109 | 73.98875053 | 25.44596218 | 15.28243206 | 39.82545408 | 38.23904826 | 1.041486541 | 0.058644195 | 0.947143416 | 0.023584255 | 0.167477605 |
| HMDB0246913 | Tetraethylene glycol monododecyl ether | 7.717076092 | 12.17974311 | 39.94557912 | 13.69319583 | 7.545146196 | 35.82318579 | 19.94746611 | 19.02050927 | 1.048734596 | 0.068649621 | 0.947555938 | 0.023395142 | 0.028491071 |
| HMDB0244644 | 2-Ethylsulfanyl-1H-benzoimidazole | 1377.918667 | 1655.286892 | 1739.9022 | 1841.281417 | 531.6406853 | 2301.72749 | 1591.03592 | 1558.216531 | 1.021062149 | 0.030070682 | 0.954567796 | 0.020193221 | 0.442117957 |
| HMDB0059611 | Thiomorpholine 3-carboxylate | 296.2784827 | 74.49893594 | 50.90183292 | 221.2914274 | 202.0532719 | 13.36870125 | 140.5597505 | 145.5711335 | 0.965574335 | -0.05054076 | 0.963353854 | 0.016214161 | 0.213574384 |
| HMDB0029113 | Tyrosyl-Proline | 163.8418681 | 7.591908831 | 5.946159135 | 79.14339882 | 79.13874412 | 26.25811245 | 59.12664534 | 61.51341846 | 0.961199147 | -0.05709273 | 0.967611454 | 0.014298999 | 0.94265101 |
| HMDB0031537 | 3-Methylcyclohexanethiol | 376.9972688 | 442.9439312 | 489.2073797 | 507.1754851 | 143.3782484 | 639.3252246 | 436.3828599 | 429.9596527 | 1.014939093 | 0.021393153 | 0.968279578 | 0.013999228 | 0.432721514 |
| HMDB0030669 | 3-Feruloylquinic acid | 12.44903933 | 15.37187328 | 13.80338655 | 19.83452398 | 16.93475191 | 5.384286903 | 13.87476639 | 14.0511876 | 0.987444391 | -0.01822859 | 0.970564229 | 0.012975719 | 0.345680585 |
| HMDB0244061 | 1,2-Cyclohexanediol | 145.2279819 | 67.22261313 | 60.63382681 | 193.3106726 | 38.31628638 | 48.05163982 | 91.02814061 | 93.2261996 | 0.976422304 | -0.03442284 | 0.971092226 | 0.012739522 | 0.314507875 |
| HMDB0254667 | Methylthiouracil | 544.8222264 | 661.2725035 | 720.655874 | 750.5507471 | 217.2917561 | 935.6391969 | 642.2502013 | 634.4939001 | 1.012224391 | 0.017529143 | 0.973736849 | 0.011558395 | 0.416703481 |
| HMDB0034295 | Floionolic acid | 30.97983397 | 34.26788465 | 44.37308564 | 41.88440448 | 17.54918336 | 50.84835411 | 36.54026809 | 36.76064732 | 0.994005023 | -0.00867495 | 0.984602241 | 0.00673918 | 0.251893939 |
| HMDB0000064 | Creatine | 1313.293732 | 1054.941386 | 1004.986965 | 1473.527706 | 1355.94764 | 527.5460844 | 1124.407361 | 1119.007144 | 1.004825901 | 0.006945557 | 0.98704558 | 0.005662792 | 0.304898331 |
| HMDB0240648 | Stearoylcholine | 41.56600171 | 62.94573182 | 52.18005632 | 55.98534905 | 29.17847697 | 72.17013262 | 52.23059662 | 52.44465288 | 0.995918435 | -0.0059005 | 0.98851124 | 0.005018388 | 0.178646872 |
